# Structure and conformational analysis of spiroketals from 6-*O*-methyl-9(*E*)-hydroxyiminoerythronolide A

**DOI:** 10.3762/bjoc.11.157

**Published:** 2015-08-19

**Authors:** Ana Čikoš, Irena Ćaleta, Dinko Žiher, Mark B Vine, Ivaylo J Elenkov, Marko Dukši, Dubravka Gembarovski, Marina Ilijaš, Snježana Dragojević, Ivica Malnar, Sulejman Alihodžić

**Affiliations:** 1 GlaxoSmithKline Research Centre Zagreb Ltd, Prilaz baruna Filipovića 29, 10000 Zagreb, Croatia; 2 GlaxoSmithKline, New Frontiers Science Park, Harlow, CM19 5AW, United Kingdom

**Keywords:** configuration, conformation, 6-*O*-methyl-9(*E*)-hydroxyiminoerythronolide A, reaction mechanism, spiroketal

## Abstract

Three novel spiroketals were prepared by a one-pot transformation of 6-*O*-methyl-9(*E*)-hydroxyiminoerythronolide A. We present the formation of a [4.5]spiroketal moiety within the macrolide lactone ring, but also the unexpected formation of a 10-C=11-C double bond and spontaneous change of stereochemistry at position 8-C. As a result, a thermodynamically stable structure was obtained. The structures of two new diastereomeric, unsaturated spiroketals, their configurations and conformations, were determined by means of NMR spectroscopy and molecular modelling. The reaction kinetics and mechanistic aspects of this transformation are discussed. These rearrangements provide a facile synthesis of novel macrolide scaffolds.

## Introduction

Macrolide antibiotics are natural or semi-synthetic products of polyketide origin, containing one or more desoxy sugars attached to a macrocyclic lactone aglycon. This large and structurally diverse category of compounds has traditionally been divided into classes based on the aglycon size, principally 12-, 14-, or 16-membered ring macrolides [1]. The most extensively explored naturally occurring 14-membered macrolide erythromycin A was discovered more than 50 years ago. Subsequently it has become the focus of numerous structural modifications, with the primary aim of increasing its antibacterial spectrum, acid stability, masking the foul taste, improving the pharmacodynamic properties and reducing the associated side effects. The two most successful semisynthetic macrolide antibiotics derived from erythromycin A are azithromycin [2–3] and clarithromycin [4]. Recent investigations have shown that beside their antibacterial activity, some macrolides exhibit anti-inflammatory/immunomodulatory [5–10], antitumor [11–13], antiviral [10] or antimalarial [14–15] activity. These discoveries have sparked a new interest in the structural modification of macrolides.

As a structural subunit, spiroketal ring systems [16–17] are present in a wide range of natural compounds. Their rigidity makes them useful for conformational control in heterocycles, but also for stabilizing a highly specific conformation within otherwise flexible larger natural compounds. For example, in cases of calyculin and okadaic acid it has been proposed [18–19] that the spiroketal unit acts as a β-turn mimic.

Naturally occurring spiroketals exhibit a wide spectrum of biological activity: anticancer [20–22], antibiotic [23–24], antifungal [25], anthelmintic [26] and anti-HIV [27]. A previously mentioned example of a protein phosphatase inhibitor okadaic acid is a toxin associated with diarrheic shellfish poisoning [28]. Another example are structurally complex tubulin polymerization-inhibiting macrolides such as spongistatins, a family of compounds isolated from marine sponges, which display extraordinary antitumor activity [29]. At the same time, the spiroketal-containing integramycin acts as an HIV-1 protease inhibitor [27].

The studies of Hayashi et al. have shown that the integrity of the spiroketal subunit is essential for the inhibition of telomerase by rubromycins [30] and it seems that simplified but characteristic spiroketals derived from the parent natural products can retain biological activity [31]. Therefore, the spiroketal unit can be regarded as a biologically validated framework and having a macrolide with spiroketal moiety can lead to new compounds with potentially interesting biological profiles.

## Results and Discussion

**Scheme 1:**
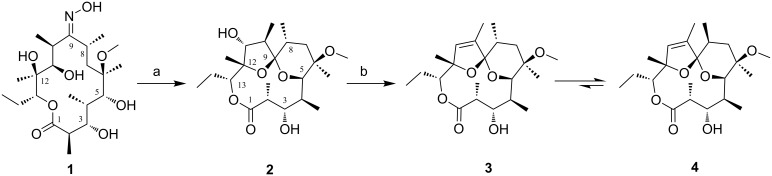
Synthetic route to spiroketals **2**–**4**. Reaction conditions: a) Na_2_S_2_O_5_/HCOOH/EtOH/water/70 °C, b) DCl/CDCl_3_/rt.

For several decades now, our continuing primary interest lay in 14-membered macrolides and their semi-synthetic 15-membered derivatives targeting therapeutic areas of bacterial [32–34] and parasitic [35] infections, as well as inflammation [36–38]. In the search for novel scaffolds to be used as a fresh starting point for further derivatisation [39], we explored modifications to the macrocyclic ring. Oxidative deoximation of **1** in mild acidic media, shown in Scheme 1, led us to an unexpected macrolide-spiroketal **2** which sparked our interest, not only because the spiroketal is a biologically relevant subunit whose incorporation into macrolide could yield interesting biological activity, but also because this unit provides unique rigidity to the 6-C to 12-C region of the macrocycle allowing for specific orientations of the functional groups.

The spiroketal unit in erythromycin derivatives is not unknown in the literature, though. As the principal product of erythromycin A acid degradation, anhydroerythromycin A has been studied [40–41] since erythromycin A started being used in human medicine as an antibiotic. This 14-membered macrolide contains a [4.4]spiroketal unit connecting 6-C–O–9-C–O–12-C. Stereochemistry at position 9-C of this spiroketal was determined by combination of NMR spectroscopy and molecular modelling [42]. The kinetics of its formation has also been extensively studied [43–46]. Most recently, in the latest revision of the reaction pathway Hassanzadeh et al. showed that this macrolide spiroketal in acid media exists in equilibrium with the 9,12-hemiacetal of erythromycin A [47]. The closely related antibiotic clarithromycin (structurally related to our starting aglycon **1**) in acid medium does not degrade to the spiroketal but instead loses the cladinose sugar [48–49]. The degradation process terminates with decladinosyl clarithromycin in equilibrium with the 9,12-hemiacetal decladinosyl clarithromycin [50].

Unlike the acid degradation product anhydroerythromycin A, the spiroketal **2** exhibits the much less common [4.5]spiroketal unit connecting 5-C–O–9-C–O–12-C; its existence is made possible by the free 5-OH functionality of the starting aglycon **1** [39,51]. Native macrolide aglycons are rarely found in nature [52], most likely because they are quickly glycosylated even before macrolactonisation [53]. The [4.5]ring system identified in the spiroketal **2** has been rarely explored in the literature: one example being the corresponding derivative of erythronolide B [54] unexpectedly formed during the acid-catalysed removal of a 3,5-acetonide protecting group, with the structure and stereochemistry confirmed by X-ray crystallography. The two others were obtained during a total synthesis of erythronolide Al [55–56], as a result of deprotection of the 3,5-benzylidene acetal of erythronolide A (with or without a triethylsilyl protecting group on 6-O). However, no additional data was presented to confirm the stereochemistry at 8-C and 9-C.

While characterising spiroketal **2** we noticed that, though stable in DMSO-*d*_6,_ it degrades in CDCl_3_ (results not shown). Unstabilised CDCl_3_ slowly decomposes to produce acidic byproducts, ultimately producing traces of hydrochloric acid which we assumed was the cause of further transformations of spiroketal **2**. Since the erythromycin A acid degradation ends the with formation of the spiroketal, we were interested to characterise these unexpected products derived from spiroketal **2**. Erythronolides as polyhydroxyketones could undergo skeletal transformations such as epimerisations, spiroketalisation, translactonisation, dehydroxylations, etc. [57]. Grover et al. [58] have previously shown that during the oximation of erythromycin A, unexpected side reactions led to the formation of a 6,9-intramolecular enol ether, translactonisation or formation of anhydroerythromycin A, perhaps resulting from acetic acid traces generated during the reaction acting upon either the starting material, or deoximation of the intended product. We therefore repeated the experiment in fresh, stabilised chloroform to study the reaction kinetics and to isolate and fully characterise the products.

### Structure elucidation of compound **2**

Analysis of HMBC spectra, as well as chemical shift comparison with the parent compound **1** [39], showed that the 9-C-signal of **2** exhibits a large upfield shift to 107.1 ppm (168.1 ppm in **1**), as well as intense HMBC cross-peaks with 4-H and 5-H. At the same time, the carbon signal of 12-C in **2** is found at 81.8 ppm (73.4 ppm in **1**). These suggest their participation in a [4.5]spiroketal ring system. Such types of spiroketals usually appear in two conformations – typically in a more stable anomeric and most often in a less stable nonanomeric form [23]. To determine the configuration at 9-C and elucidate the conformation of **2**, nOe analysis was performed (Tables SI1 and SI2, in Supporting Information File 1), followed by molecular modelling. Very strong nOe correlations between 2-H, 5-H and 10-H observed in both DMSO-*d*_6_ and CDCl_3_ attest to their close spatial proximity (2.0–2.6 Å). Other characteristic observed nOe interactions are: very strong 4-H↔6-Me, 8-Me↔10-Me, 3-H↔4-Me, 5-H↔7-Hax, 5-H↔6-Me, 5-H↔10-Me correlations and a weak interaction between 4-Me and 5-H.

**Figure 1:**
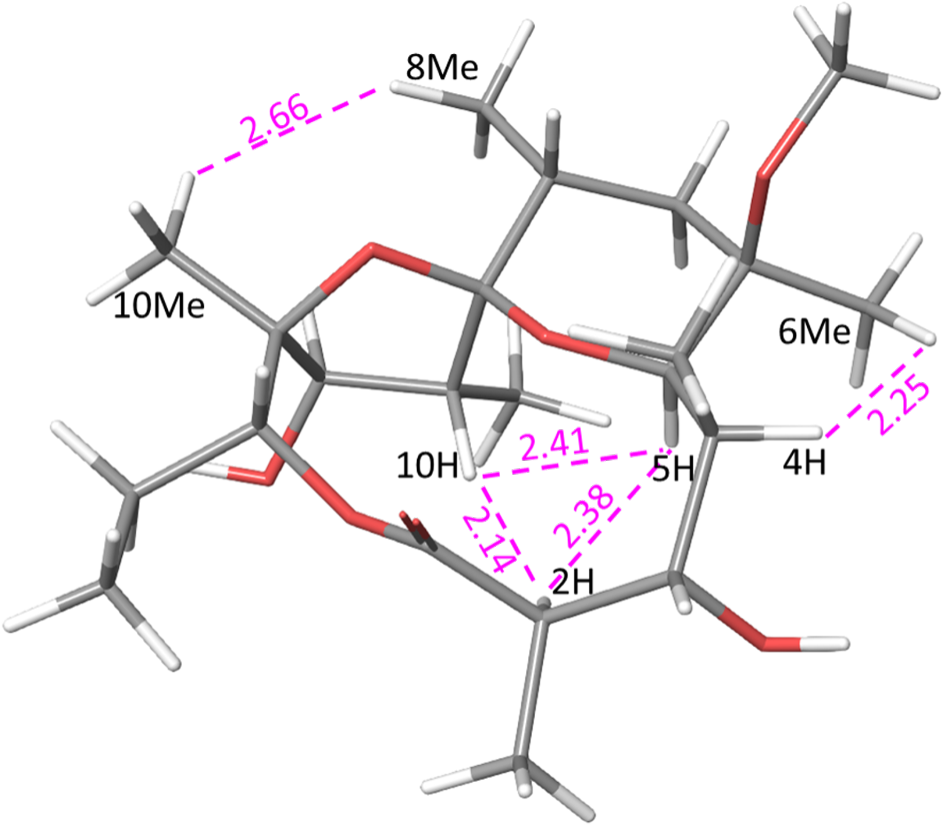
Modelling-derived structure of **2** showing key nOe interactions (calculated distances in Å).

To simplify the generation of the three-dimensional structure and avoid time-consuming molecular dynamic simulations, the starting model of **2** was created from the X-ray single crystal structure of tricyclic spiroketal (CSD entry: ERYTHR) [55], with replacement of the 6-hydroxy group by 6-methoxy. A two-step minimization process consisted of adding energy constraints to the X-ray structure followed by unconstrained minimization. Significant conformational changes were not expected due to the rigidity of the tricyclic system. A low energy conformation of **2** is presented in Figure 1.

Analogous to pyranoside conformations, the highly substituted tetrahydropyran ring is in a ^5^*C*_8_ conformation [59] and it is not anomerically stabilised. The bulky 6-OMe group and 10-C are axially oriented while 4-C, 6-Me, 8-Me and 12-O are equatorial. Experimental evidences for such ^5^*C*_8_ conformation is the large ^3^*J*_7-H,8-H_ coupling constant (14 Hz) suggesting equatorial orientation of 8-Me. The strong 1,3-axial interactions between 5-H and axial 7-H confirms their proximity and equatorial orientation of 4-C. Strong correlation between 6-OMe and equatorial 7-H with absence of interactions with axial 7-H suggests that 6-OMe is axially oriented. The alternative ^8^*C*_5_ conformation would be unfavourable due to adverse 1,3-diaxial interactions between the four substituents.

Having determined the stereochemistry at position 9-C to be *R,* we compared the chemical shifts of **2** to the 6-OTES protected spiroketal reported by Carreira [56]. The close correspondence of the chemical shifts between the two suggests that both compounds share the same configuration.

### Reaction kinetics

**Figure 2:**
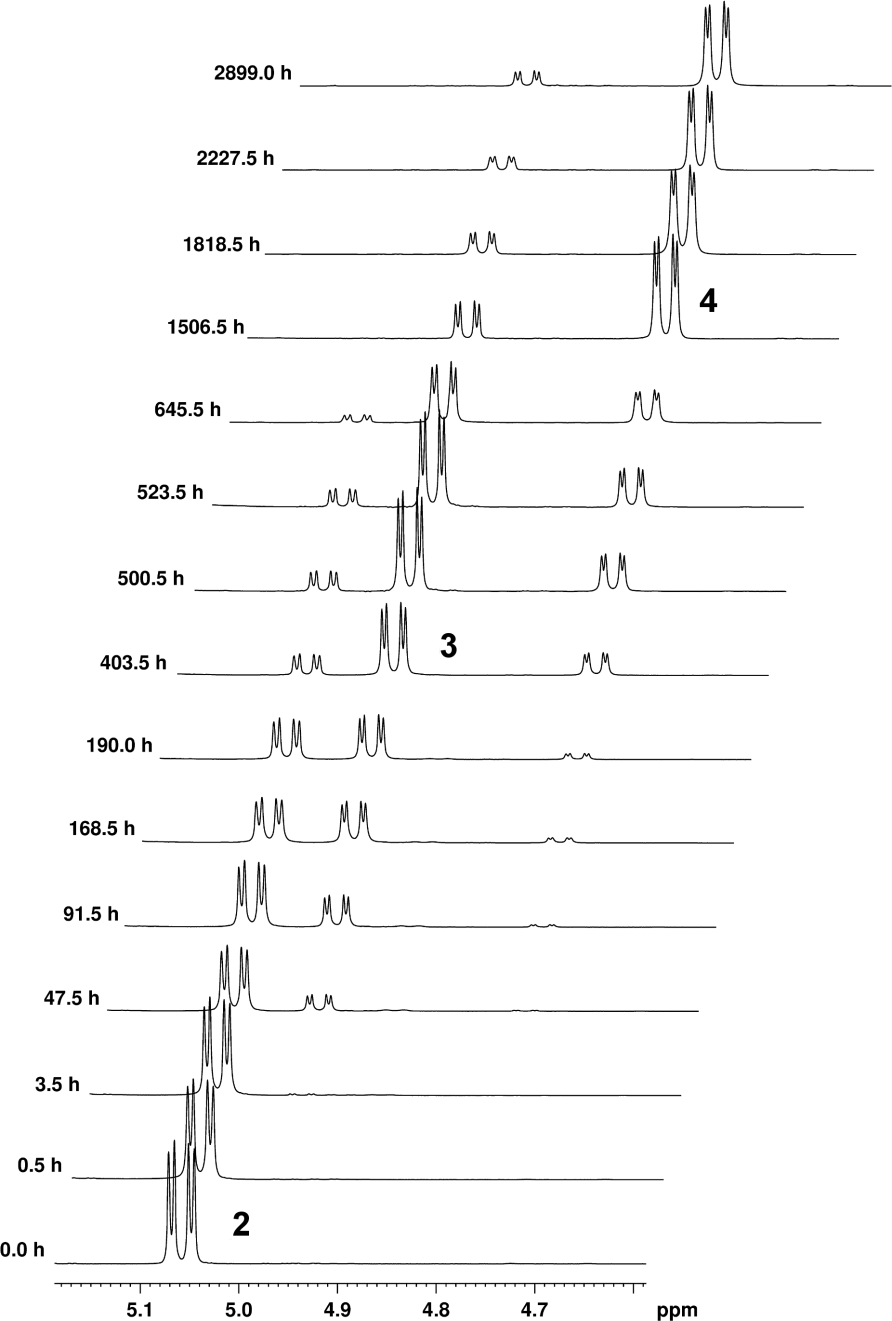
Time-dependent ^1^H NMR spectra of **2**, **3** and **4** (13-H multiplets region). The experiments were performed on 0.1 M chloroform-*d*_1_ solution of **2** at 25 °C. Spectra were recorded at appropriate time intervals after the initial addition of 10 μL of 1 M HCl.

Dissolution in fresh stabilised CDCl_3_ showed no evidence of change, suggesting that observed transformations were caused by acidic species arising from chloroform decomposition. It was shown [16,23,60] that acidic media can catalyse polyhydroxy ketone transformations to spiroketals. Therefore, we undertook the study of the acid-promoted transformation of **2** by accumulation of time-dependent ^1^H NMR spectra at constant temperature (Figure 2).

**Figure 3:**
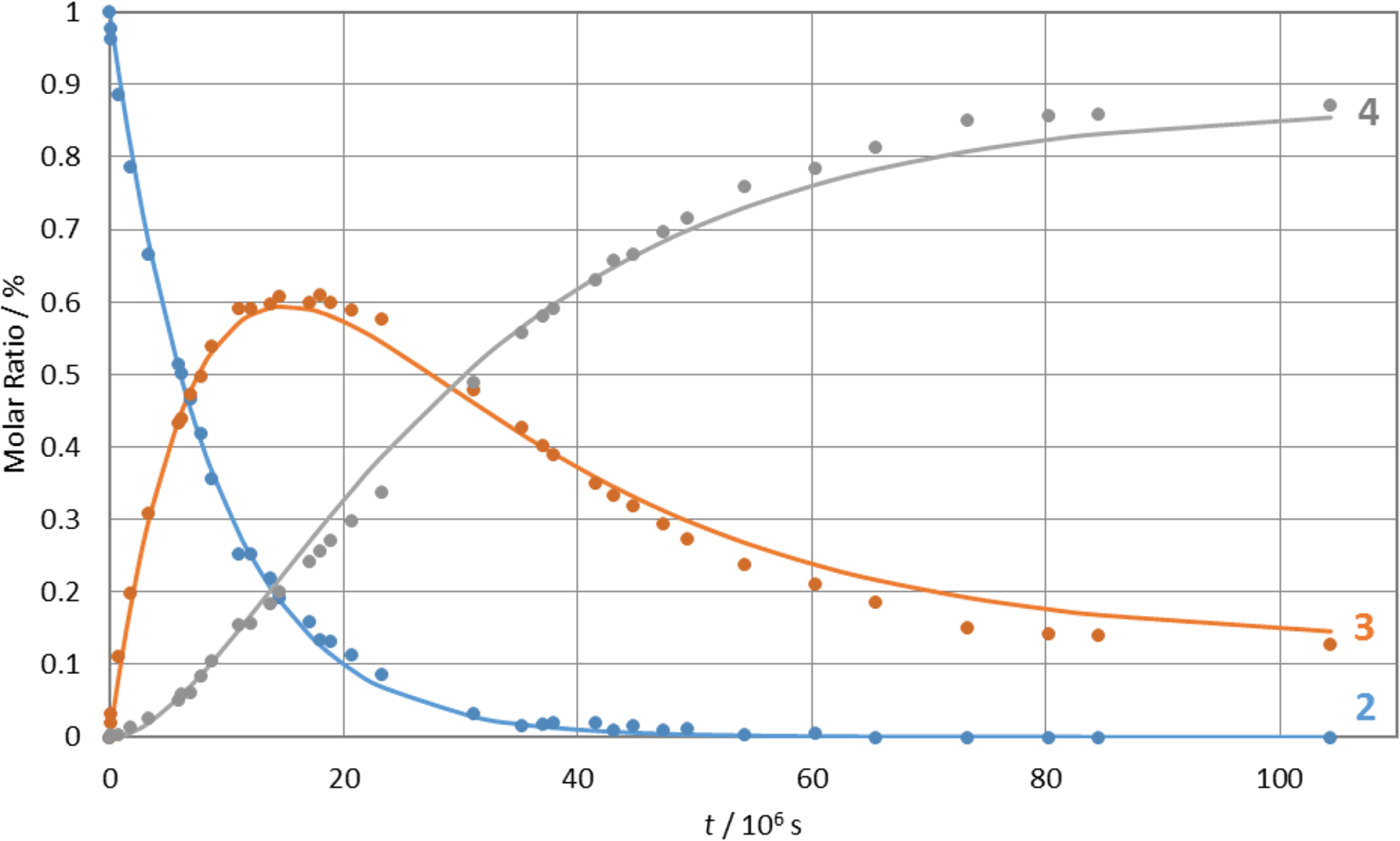
Interconversion kinetics of compounds **2** (blue), **3** (orange) and **4** (grey).

The mole fractions of the species in the mixture were calculated from the collected spectra using the 13-H integrals and used to examine the kinetics (Figure 3), as well as gain insight into the reaction mechanism. The model of consecutive first-order reactions with a reversible second step [61]:


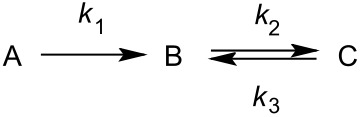


was used to describe the observed time-dependencies of each species in the mixture, giving calculated rate constants *k*_1_ of 1.15 × 10^−6^ s^−1^ for the first reaction step, with *k*_2_ and *k*_3_ of 3.63 × 10^−7^ s^−1^ and 5.28 × 10^−8^ s^−1^, respectively, for the second (equilibrium) reaction (*R*^2^ = 0.9978113).

Conversion of **2** into compounds **3** and **4** proceeds to completion after approximately 47 days ([2]/[2] _0_ = 1% at 1123 h). The concentration of compound **3** first increases reaching the maximum after 18 days and then decreases, while the concentration of compound **4** steadily increases throughout the experiment. The reaction ends in an apparent equilibrium with relative concentrations of 13% and 87%, for **3** and **4**, respectively. These findings indicate that **3** is the kinetic and **4** the thermodynamic product. In an effort to effect full conversion of **3** to **4**, after 120 days the mixture was heated in a microwave reactor for 30 minutes at 120 °C. However, the product ratio was found to be unchanged.

### Structure elucidation of compound **3**

**Figure 4:**
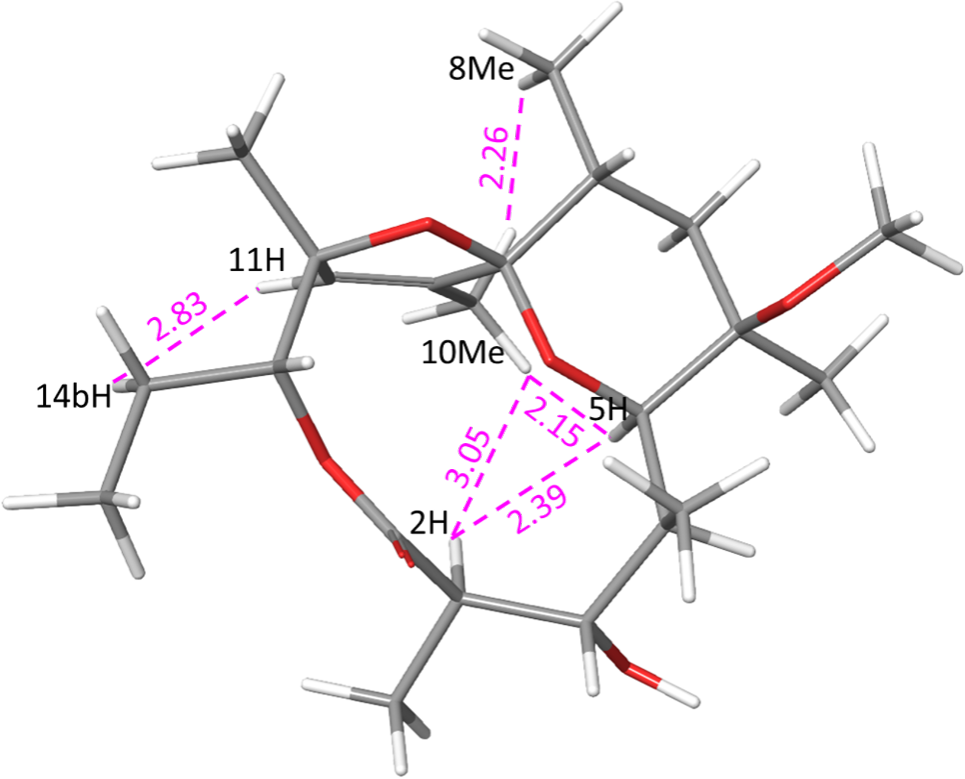
Modelling-derived structure of **3** showing key nOe interactions (calculated distances in Å).

The main feature of compound **3** is the appearance of a double bond between 10-C and 11-C, as evidenced by the signal in the ^1^H NMR spectrum at 5.65 ppm and relevant HMBC correlations. The initial 3D structure of the compound was generated from the previously modelled conformation of **2** by eliminating 10-H and 11-OH, forming an endocyclic double bond. Minimization of this structure resulted, as expected, in small changes at positions 10-C and 11-C (Figure 4), while all other atoms remained in almost identical positions. The tetrahydropyran ring in **3** also assumes ^5^*C*_8_ conformation which explains the observed system of nOe correlations between 2-H, 5-H and 10-Me (2.2–2.9 Å), as well as 8-Me↔10-Me (2.7 Å) and 11-H ↔14_b_-H (2.8 Å) interactions, all shown in Table SI3, in Supporting Information File 1. The coupling constants ^3^*J*_8-H,7-Ha_ and ^3^*J*_8-H,7-Hb_ (one strong, antiperiplanar and one weak, synclinal) suggest an axial orientation of 8-H and equatorial position of 8-Me. Similar to **2**, the six-membered tetrahydropyran assumes an anomerically non-stabilised chair conformation with the larger 6-methoxy substituent in the axial position.

### Structure elucidation of compound **4**

Determining the structure of the thermodynamically stable product **4** proved to be more challenging. Our initial hypothesis was that it was an anomerically stabilised *^8^C*_5_ conformer of **3** with the 6-methoxy group in the more stable equatorial position. Molecular mechanics calculations, however, showed that such a structure should be energetically unfavourable due to 1,3-repulsion between the axial 6-methyl and 8-methyl groups. Therefore, nOe interactions (Table SI4, Supporting Information File 1) and proton–proton coupling constants (Table SI5, Supporting Information File 1) were introduced into the modelling calculations as distance and angle constraints.

**Figure 5:**
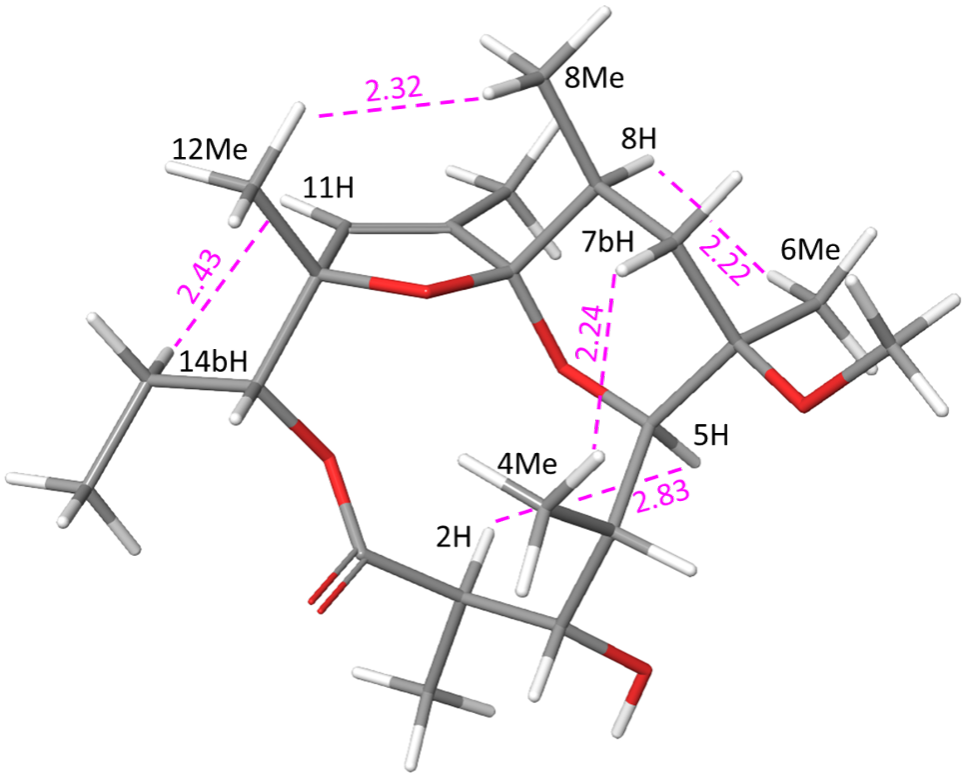
Modelling-derived structure of compound **4** showing key nOe interactions (calculated distances in Å).

Various conformations of the proposed structure were analysed by molecular mechanics calculations, but only one structure (Figure 5), with inverted stereochemistry at the 8-C position (8*S*) was consistent with all measured nOe contacts and vicinal coupling constants.

Numerous nOe interactions support the proposed structure. Correlations of 5-H with both substituents at position 6 suggest that this hydrogen atom is in equatorial position, placing 4-C axially. For comparison, only the strong interaction 5-H↔6-Me is observed in both **2** and **3**. Furthermore, the absence of nOe correlation between 5-H and either of the 7-Hs (contrary to both **2** and **3**) confirms its equatorial position. Strong interactions of 8-H with both 6-Me and 10-Me, as well as large ^3^*J*_7-H, 8-H_ suggest equatorial orientation for 8-Me.

### Structural comparisons

**Figure 6:**
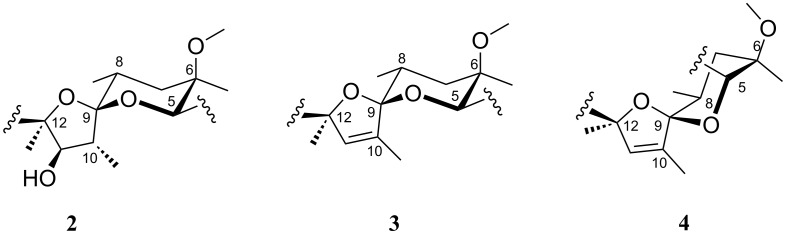
Comparison of the spiroketal ring system stereochemistry and conformations in compounds **2**–**4**.

The most significant structural changes between **3** and **4** are a consequence of the configurational change within the tetrahydropyran ring (Figure 6).

**Figure 7:**
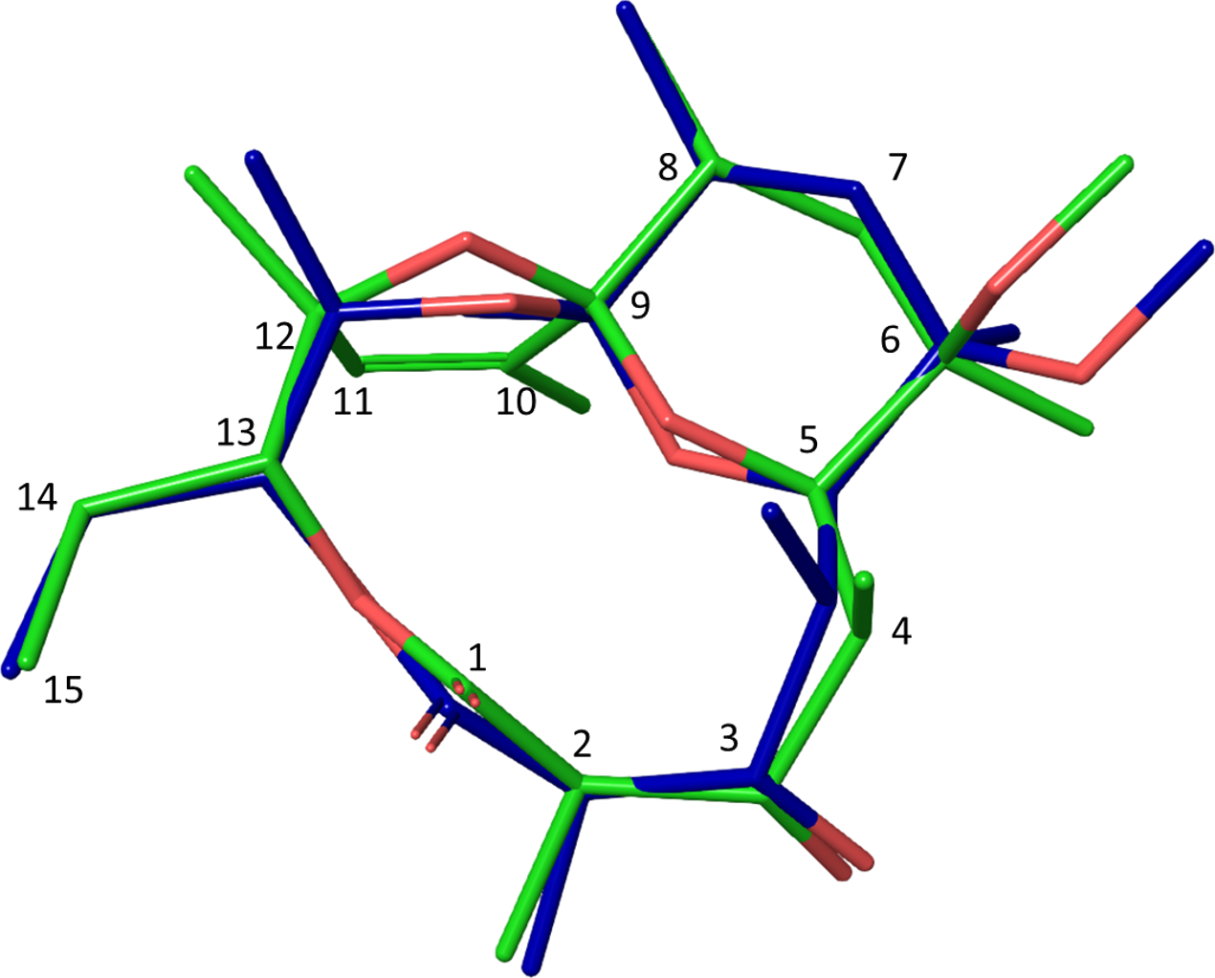
Overlay of the computed structures of **3** (green) and **4** (blue).

Compound **4** is approximately 0.9 kcal mol^−1^ more stable than **3** (calculated in CHCl_3_ using the MMFF94 force field). Obviously, the anomeric effect has a strong influence on the preferred conformation of the spiroketal ring. In **4** the tetrahydropyran ring adopts the *^8^C_5_*-conformation with the 6-methoxy group in the equatorial and the three substituents in the axial positions (12-O bridge, 4-C and 6-Me). Inversion of the 8-C configuration results in 8-Me adopting the equatorial position, removing unfavourable steric interactions (Figure 7).

### Reaction mechanism

**Scheme 2:**
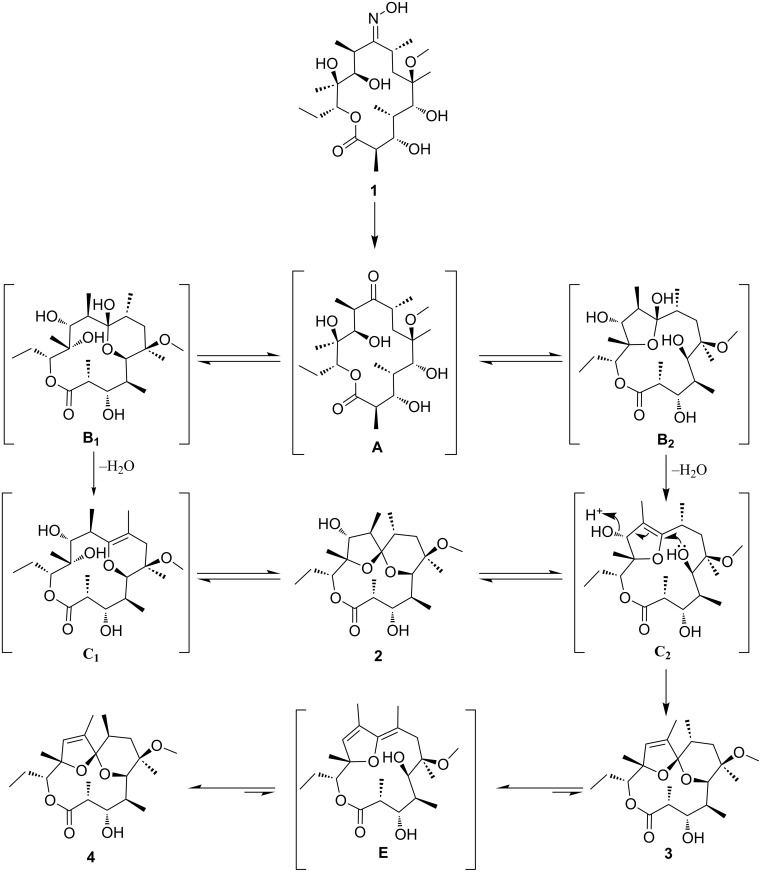
Postulated mechanism for the formation of compounds **2**–**4**.

Although uncommon, epimerisation at position 8-C was reported earlier [62] in structurally similar compounds when exposed to analogous reaction conditions. A plausible mechanism of the transformations as discussed is presented in Scheme 2.

The whole equilibrium process most likely consists of a series of hemiketal and enol ether transformations. Thus the elusive intermediate **A** in acidic media forms a hemiketal **B** (**B1** or **B2**). From our data and literature precedents it is not clear if the 6-membered (**B1**) or the 5-membered (**B2**) intermediate is formed first. Based on chemical calculations Hassanzadeh et al. suggested 12-*O*-enol ether formation (type **C2**) and final spiroketalisation [42] via the corresponding oxocarbenium type transition state. Carreira et al. on the other hand isolated a 6-membered enol ether (type **C1**) and further transformed it into a [4.5]spiroketal [56]. Whichever pathway is applicable, it transpires through loss and regain of chirality either at 8-C (**C1**) or 10-C (**C2**) and final formation of the spiroketal **2**. Loss and regain of chirality at 8-C proceeds with complete retention of the configuration with the methyl group in an equatorial position on the tetrahydropyran ring. Prolonged acidic treatment of **2** leads either back to **C1** or to its isomer **C2**. The latter loses a molecule of water via an intramolecular S_N_2’ mechanism forming the unsaturated spiroketal **3**. Repeated spiroketal opening leads to the conjugated enol ether **E** with loss of the chiral centre at 8-C. Reports on clarithromycin acid degradation suggest that enol ethers of this type exist as mixtures of 8*E*, 10*Z* and 8*Z*, 10*Z* isomers [49], that might even equilibrate [50]. It is not clear which step is C-8 stereo determining but we might assume that spiroketalisation of each of *E* isomers goes through an oxocarbenium type transition state that have different stereochemistry on C-8. That reflects in formation of the two isomeric products **3** and **4**, with the latter anomerically stabilized and thermodynamically more stable. In this case the complete epimerization to 8*S* occurs with the methyl group again occupying an equatorial position.

A structurally similar acid-catalyzed erythromycin A decomposition product, erythralosamine [40–41] containing a [4.4] rather than [4.5]spiroketal ring and double bond between 10-C and 11-C has been characterised by X-ray analysis [63]. Only one enantiomer at 8-C was isolated, showing retention of stereochemistry. This would support the hypothesis that the difference in energies of the 6-membered ring anomers relative to a 5-membered ring is sufficient to drive the conversion of **3** to **4**.

## Conclusion

In this paper we have presented the acid-promoted modification of 6-*O*-methyl-9(*E*)-hydroxyiminoerythronolide A into three rigid tricyclic spiroketal systems **2**, **3** and **4**. Compound **2** proved to be stable in non-acidic media. After acidic treatment two new dehydrated molecules are formed: compound **3** which with time spontaneously transforms into the thermodynamically more stable **4**. The most significant structural change is inversion of configuration at 8-C accompanied by a conformational change of the tetrahydropyran ring from one chair conformation ^5^*C*_8_ into another, anomerically stabilised, ^8^*C*_5_ conformation. These transformations give a facile route for the synthesis of novel macrolide spiroketals.

## Experimental

### Materials

Oxime **1** was synthesized according to the previously published procedure [39,51,64]. The structures of all compounds were confirmed by NMR spectroscopy, LC–HRMS and molecular modelling. Both NMR spectroscopy and LC-MS were used to ascertain purity (greater than 95% for all compounds).

### Reaction kinetics

30 mg of **2** was dissolved in 0.7 mL chloroform-*d*_1_ giving the final compound concentration of 0.1 M. Comparison of the proton spectrum taken immediately after dilution and the proton spectrum taken after 3 days showed no differences at all. Moments prior to the beginning of the kinetics experiment 10 μL of 1 M HCl was added into the NMR tube. Immediately the reaction started and was monitored by ^1^H NMR spectroscopy at 25 °C. The rate constants were calculated using Microsoft Excel 2013 (32 bit) for Microsoft Windows 8.1 (64 bit) with Solver add-in (see Supporting Information File 1).

### NMR Spectroscopy

One and two-dimensional NMR spectra (^1^H, APT, COSY, NOESY, ROESY, edited-HSQC and HMBC) were recorded on Bruker Avance III 600 and Bruker Avance DRX 500 spectrometers, both equipped with 5 mm diameter inverse detection probe with z-gradient accessory, as well as a Bruker Avance DPX 300 spectrometer using a 5 mm DUL ^1^H/^13^C probe. All spectra were recorded using standard Bruker pulse sequences on compounds dissolved in DMSO-*d*_6_ and CDCl_3_ at 25 °C, with TMS as the internal standard. NOESY spectra were obtained with a 400 ms mixing time. NMR analysis of compound **2** was performed in both DMSO-*d*_6_ and CDCl_3._ Initial full assignment and conformational analysis of compound **3** was performed at the point of maximum concentration of compound **3**. The final kinetics reaction mixture was evaporated to dryness and re-dissolved in DMSO-*d*_6_ for the structure elucidation of **4**, as signal broadening occurred in the NMR spectra recorded in CDCl_3_. The compounds **3** and **4** were later re-synthesised, isolated by preparative HPLC–MS and fully assigned as individual compounds.

### Molecular modelling

The model of **2** was generated from the single crystal X-ray structure of erythronolide A anhydro derivative (CSDC refcode: ERYTHR [55]) and minimized with constraints of 1000 kcal on all atoms with known X-ray coordinates. The model of **3** was generated from the model of compound **2.** The model of **4** was obtained by molecular modelling with NMR constraints: strong nOe correlations were converted to a distance constraint of 2.0–2.5 Å, medium as 2.5–3.5 Å and weak as 3.5–5.0 Å, while vicinal coupling constants were converted to angle constraints. All calculations were performed using the MMFF94 force field implemented in Schrodinger Maestro 2015-1 [65].

### Preparative LC–MS

Compounds were purified using a Waters Mass Directed AutoPurification System using a XBridge MS C18 column (19 × 100 mm, 5 µm) and isocratic technique (using mixture of eluents 40% 10 mM NH_4_HCO_3_/pH 10 and 60% CH_3_CN). The flow rate was maintained at 17 mL/min.

### LC–HRMS

Positive ion mass spectra were acquired on a Micromass Q-Tof-2 equipped with a Z-spray interface, operated in positive ion mode over a mass range of 100–1000 Da, with a scan time of 1.0 s and an interscan delay of 0.1 s. Reserpine was used as the external calibrant lock mass ([M + H]^+^ = 609.2812 Da). Ionization was achieved with a spray voltage of 3.5 kV, a cone voltage of 20 V, with cone and desolvation gas flows of 20–30 and 500 L/h, respectively. The source block and desolvation temperatures were maintained at 125 °C and 150 °C, respectively. The elemental composition was calculated using MassLynx v4.1 for the [M + H]^+^ (or other mentioned adducts) and the mass error quoted as ppm. Chromatography was performed using an Agilent 1100 HPLC instrument equipped with a XBridge 2.1 × 150 mm 3.5 μm column (Waters, Milford, USA). Gradient elution was carried out with the mobile phases as (A) 10 mM NH_4_HCO_3_, pH 10 and (B) CH_3_CN.

### Synthesis

#### Preparation of compound **2**

6-*O*-Methyl-9(*E*)-hydroxyiminoerythronolide A (**1**) (0.6 g, 1.341 mmol) was dissolved in EtOH (20 mL). Water was added (24 mL), followed by HCOOH (142.2 µL, 3.75 mmol) and Na_2_S_2_O_5_ (1.02 g, 5.37 mmol) with continuous stirring at room temperature. The reaction mixture was heated to about 70 °C and after 45 minutes the additional amount of Na_2_S_2_O_5_ (1.02 g, 6.38 mmol) was added. After 4 hours the heating was turned off and the reaction mixture was left to stir overnight at room temperature. The reaction mixture was concentrated and extracted with DCM (6 × 20 mL). Combined organics were washed with sat. aq. NaHCO_3_ (3 × 10 mL), brine (2 × 10 mL) and dried over Na_2_SO_4_. Evaporation of solvent afforded crude product **2** which was further purified by column chromatography on silica-gel (LC-Packing 60 mL, 20 g, Supelco) eluting with 100→95% CH_2_Cl_2_/(MeOH/NH_4_OH 9:1.5). The product was obtained as white powder. 0.197 g (33.9%).

^1^H NMR (600 MHz, DMSO-*d*_6_) δ 0.71 (t, *J* = 7.4 Hz, 3H, 15-H), 0.89 (d, *J* = 7.5 Hz, 3H, 8-Me), 0.98 (s, 3H, 6-Me), 1.04 (d, *J* = 7.5 Hz, 3H, 4-Me), 1.16 (s, 3H, 12-Me), 1.23 (d, *J* = 7.0 Hz, 3H, 2-Me), 1.27 (d, *J* = 7.3 Hz, 3H, 10-Me), 1.48–1.54 (m, 1H, 14-H), 1.54–1.60 (dd, *J* = 14.0 Hz, 1H, 7-H), 1.76–1.84 (m, 1H, 8-H), 1.88 (dd, *J* = 14.8, 3.5 Hz, 1H, 7-H), 1.93–1.99 (m, 1H, 14-H), 1.97–2.02 (m, 1H, 4-H), 2.34 (dq, *J* = 9.2, 6.9 Hz, 1H, 2-H), 2.97–3.05 (m, 1H, 10-H), 3.01 (s, 3H, 6-OMe), 3.27 (ddd, *J* = 9.2, 5.4, 1.3 Hz, 1H, 3-H), 3.36 (dd, *J* = 10.6, 6.0 Hz, 1H, 11-H), 3.52 (s, 1H, 5-H), 4.63 (dd, *J* = 12.0, 3.1 Hz, 1H, 13-H), 4.77 (d, *J* = 5.4 Hz, 1H, 3-OH), 5.26 (d, *J* = 6.1 Hz, 1H, 11-OH); ^13^C NMR (150 MHz, DMSO-*d*_6_) δ 10.6 (15-C), 14.8 (8-Me), 15.1 (4-Me), 15.4 (10-Me), 18.5 (2-Me), 20.5 (6-Me), 23.7 (12-Me), 23.8 (14-C), 34.1 (8-C), 34.8 (7-C), 41.5 (4-C), 44.9 (2-C), 47.0 (10-C), 47.9 (6-OMe), 74.4 (6-C), 76.0 (5-C), 77.9 (3-C), 80.1 (13-C), 81.8 (12-C), 85.8 (11-C), 107.1 (9-C), 173.3 (1-C); ^1^H NMR (500 MHz, CDCl_3_) δ 0.86 (t, *J* = 7.3 Hz, 3H, 15-H), 0.98 (d, *J* = 7.3 Hz, 3H, 8-Me), 1.07 (s, 3H, 6-Me), 1.26 (d, *J* = 7.5 Hz, 3H, 4-Me), 1.31 (s, 3H, 12-Me), 1.40 (d, *J* = 6.7 Hz, 3H, 2-Me), 1.41 (d, *J* = 7.3 Hz, 3H, 10-Me), 1.60 (dd, *J* = 14.8, 13.9 Hz, 1H, 7-H), 1.74 (dqd, *J* = 14.0, 7.4, 3.5 Hz, 1H, 14-H), 1.86 (dqd, *J* = 14.6, 11.9, 7.3 Hz, 1H, 14-H), 1.85 (br. s., 1H, 3-OH), 1.95 (dd, *J* = 14.9, 3.6 Hz, 1H, 7-H), 1.99 (d, *J* = 11.8 Hz, 1H, 11-OH), 2.08 (q, *J* = 7.6 Hz, 1H, 4-H), 2.12 (dqd, *J* = 10.1, 7.1, 3.5 Hz, 1H, 8-H), 2.46 (dq, *J* = 9.2, 6.9 Hz, 1H, 2-H), 2.87 (dq, *J* = 9.8, 7.5 Hz, 1H, 10-H), 3.09 (s, 3H, 6-OMe), 3.52 (dd, *J* = 11.5, 9.8 Hz, 1H, 11-H), 3.54 (s, 1H, 5-H), 3.69 (dd, *J* = 9.5, 1.5 Hz, 1H, 3-H), 5.06 (dd, *J* = 12.1, 3.5 Hz, 1H, 13-H); ^13^C NMR (126 MHz, CDCl_3_) δ 10.5 (15-C), 15.0 (4-Me), 15.1 (8-Me), 16.1 (10-Me), 18.4 (2-Me), 20.9 (6-Me), 23.9 (12-Me), 25.0 (14-C), 34.2 (8-C), 35.6 (7-C), 42.6 (4-C), 45.3 (2-C), 48.2 (6-OMe), 50.5 (10-C), 74.4 (6-C), 77.2 (5-C), 79.3 (3-C), 82.1 (13-C), 83.0 (12-C), 89.1 (11-C), 107.9 (9-C), 173.0 (1-C) ppm; HRMS: [M + H]^+^ measured 415.2679, calculated 415.2696, error −4.1 ppm

#### Preparation of compounds **3** and **4**

Two drops of 38% DCl solution in D_2_O were added to the solution of compound **2** (82.6 mg) in CDCl_3_ (5.5 mL). The reaction mixture was left to stir and its progress was monitored with ^1^H NMR, until the starting compound disappeared and the **3** to **4** ratio was approximately 50:50. The reaction mixture was then evaporated and purified by preparative LC–MS (Instrument: Waters purification system – ZQ) to give compounds **3** (16 mg, purity: 98.7%) and **4** (37 mg, purity: 98.5%) as white solids.

**Compound 3: **^1^H NMR (600 MHz, CDCl_3_) δ 0.85 (d, *J* = 7.3 Hz, 3H, 8-Me), 0.87 ( t, *J* = 7.3 Hz, 3H,15-H), 1.10 (s, 3H, 6-Me), 1.23 (d, *J* = 7.5 Hz, 3H, 4-Me), 1.27 (s, 3H, 12-Me), 1.32 (d, *J* = 7.2 Hz, 3H, 2-Me), 1.35 (ddq, *J* = 14.0, 11.9, 7.3 Hz, 1H, 14-H), 1.46 (dd, *J* = 14.1 Hz, 1H, 7-H), 1.66 (dqd, *J* = 14.0, 7.3, 3.0 Hz, 1H, 14-H), 1.95 (dd, *J* = 14.7, 3.1 Hz, 1H, 7-H), 2.02 (d, *J* = 1.4 Hz, 3H, 10-Me), 2.15–2.23 (m, 1H, 4-H), 2.15–2.21 (m, 1H, 8-H), 2.65 (dq, *J* = 7.3 Hz, 1H, 2-H), 3.10 (s, 3H, 6-OMe), 3.77 (dd, *J* = 7.4, 3.4 Hz, 1H, 3-H), 4.02 (d, *J* = 2.1 Hz, 1H, 5-H), 4.97 (dd, *J* = 11.5, 2.8 Hz, 1H, 13-H), 5.65 (d, *J* = 1.4 Hz, 1H, 11-H); ^13^C NMR (75 MHz, CDCl_3_) δ 10.4 (15-C), 14.0 (4-Me), 15.3 (8-Me), 16.3 (10-Me), 18.2 (2-Me), 21.4 (6-Me), 22.5 (12-Me), 24.4 (14-C), 32.1 (8-C), 36.4 (7-C), 41.9 (4-C), 46.3 (2-C), 48.3 (6-OMe), 74.3 (6-C), 78.8 (3-C), 78.9 (5-C), 79.0 (13-C), 88.9 (12-C), 112.1 (9-C), 131.2 (11-C), 138.8 (10-C), 174.0 (1-C); HRMS: [M + H]^+^ measured 397.2586, calculated 397.2590, error −1 ppm.

**Compound 4: **^1^H NMR (600 MHz, CDCl_3_) δ 0.78 (d, *J* = 6.6 Hz, 3H, 8-Me), 0.93 (t, *J* = 7.3 Hz, 3H, 15-H), 1.18 (d, *J* = 7.7 Hz, 3H, 4-Me), 1.24 (s, 3H, 12-Me), 1.27 (d, *J* = 6.8 Hz, 3H, 2-Me), 1.40 (s, 3H, 6-Me), 1.42 (ddq, *J* = 14.0, 11.0, 7.3 Hz, 1H, 14-H), 1.61 (dd, *J* = 12.4, 4.0 Hz, 1H, 7-H), 1.68 (dqd, *J* = 14.0, 7.3, 2.4 Hz, 1H, 14-H), 1.67–1.72 (m, 1H, 7-H), 1.72 (d, *J* = 1.6 Hz, 3H, 10-Me), 1.87 (dqd, *J* = 13.3, 6.7, 4.2 Hz, 1H, 8-H), 2.31 (q, *J* = 7.1 Hz, 1H, 4-H), 2.89 (dq, *J* = 7.5 Hz, 1H, 2-H), 3.21 (s, 3H, 6-OMe), 3.77 (dd, *J* = 7.4, 3.4 Hz, 1H, 3-H), 4.24 (br. s., 1H, 5-H), 4.77 (dd, *J* = 11.3, 2.5 Hz, 1H, 13-H), 5.53 (d, J = 1.4, 1H, 11-H); ^13^C NMR (75 MHz, CDCl_3_) δ 10.8 (15-C), 11.7 (10-Me), 14.7 (4-Me), 17.4 (2-Me), 17.5 (8-Me), 22.8 (12-Me), 24.1 (14-C), 24.8 (6-Me), 31.8 (8-C), 34.1 (7-C), 41.6 (4-C), 43.3 (2-C), 48.9 (6-OMe), 73.3 (6-C), 78.9 (13-C), 79.6 (3-C, 5-C), 89.6 (12-C), 111.1 (9-C), 125.7 (11-C), 139.6 (10-C), 176.1 (1-C); ^1^H NMR (600 MHz, DMSO-*d*_6_) δ 0.71 (d, *J* = 6.6 Hz, 3H, 8-Me), 0.80 (t, *J* = 7.3 Hz, 3H, 15-H), 1.01 (d, *J* = 7.9 Hz, 3H, 4-Me), 1.09 (d, *J* = 6.8 Hz, 3H, 2-Me), 1.18 (s, 3H, 12-Me), 1.33 (s, 3H, 6-Me), 1.35 (ddq, *J* = 14.5, 11.3, 7.2 Hz, 1H, 14-H), 1.50 (dd, *J* = 12.7 Hz, 1H, 7-H), 1.62 (dd, *J* = 12.0, 3.7 Hz, 1H, 7-H), 1.67 (dqd, *J* = 14.0, 7.5, 2.4 Hz, 1H, 14-H), 1.66 (d, *J* = 1.6 Hz, 3H, 10-Me), 1.86 (dqd, *J* = 13.4, 6.6, 3.8 Hz, 1H, 8-H), 2.18 (qdd, *J* = 7.8, 3.0, 2.0 Hz, 1H, 4-H), 2.63 (dq, *J* = 10.3, 6.8 Hz, 1H, 2-H), 3.12 (s, 3H, 6-OMe), 3.37–3.40 (m, 1H, 3-H), 4.17 (s, 1H, 5-H), 4.59 (dd, *J* = 11.3, 2.4 Hz, 1H, 13-H), 4.87 (br. s., 1H, 3-OH), 5.66 (q, *J* = 1.4 Hz, 1H, 11-H) ppm; ^13^C NMR (151 MHz, DMSO-*d*_6_) δ 10.6 (15-C), 11.4 (10-Me), 14.7 (4-Me), 17.3 (8-Me), 17.5 (2-Me), 22.5 (12-Me), 23.3 (14-C), 24.4 (6-Me), 31.2 (8-C), 33.7 (7-C), 40.6 (4-C), 42.7 (2-C), 48.5 (6-OMe), 72.7 (6-C), 77.6 (3-C), 78.2 (13-C), 79.0 (5-C), 89.3 (12-C), 110.5 (9-C), 126.0 (11-C), 138.3 (10-C), 175.6 (1-C) ppm; HRMS: [M + H]^+^ measured 397.2588, calculated 397.2590, error −0.5 ppm

## Supporting Information

File 1 Observed nOe contacts (Tables SI1–4), proton vicinal coupling constants used for molecular modelling calculations (Table SI5) and accurate mass measurements (Table SI7) for compounds **2**–**4,** as well as HRMS fragmentation for compound **2** (Figures SI1 and SI2, Table SI6). Details of the reaction kinetics calculation.

File 2 Results of molecular modelling for compounds **2**–**4** in mol2 format.

File 3 NMR spectra of compounds **2**–**4**.
