# Supplementary material for: Structure and conformational analysis of spiroketals from 6-O-methyl-9(E)-hydroxyiminoerythronolide A
Source: Beilstein J Org Chem. 2015 Aug 19;11:1447–57. doi: 10.3762/bjoc.11.157 (PMC4578343; doi:10.3762/bjoc.11.157)
Supplement: File 1 — Observed nOe contacts (Tables SI1–4), proton vicinal coupling constants used for molecular modelling calculations (Table SI5) and accurate mass measurements (Table SI7) for compounds 2–4, as well as HRMS fragmentation for compound 2 (Figures SI1 and SI2, Table SI6). Details of the reaction kinetics calculation. [file Beilstein_J_Org_Chem-11-1447-s001.pdf]

# Supporting Information

**for**

# Structure and conformational analysis of spiroketals from 6-*O*-methyl-9(*E*)-hydroxyiminoerythronolide A

Ana Čikoš*[1], Irena Ćaleta[1], Dinko Žiher[1], Mark B. Vine[2], Ivaylo J. Elenkov[1], Marko Dukši[1], Dubravka Gembarovski[1], Marina Ilijaš[1], Snježana Dragojević[1], Ivica Malnar[1] and Sulejman Alihodžić[1]

Address: [1]GlaxoSmithKline Research Centre Zagreb Ltd, Prilaz baruna Filipovića 29, 10000 Zagreb, Croatia and [2]GlaxoSmithKline, New Frontiers Science Park, Harlow, CM19 5AW, United Kingdom

Email: Ana Čikoš - ana.cikos@glpg.com

*Corresponding author

**Observed nOe contacts (Table SI1–4), proton vicinal coupling constants used for molecular modelling calculations (Table SI5) and accurate mass measurements (Table SI7) for compounds 2–4, as well as HRMS fragmentation for compound 2 (Figures SI1 and SI2, Table SI6). Details of the reaction kinetics calculation.**

**Table SI1.** nOe interactions of compound **2** in DMSO-$d_6$ at 25 °C.

| | 2 | 2Me | 3 | 3OH | 4 | 4Me | 5 | 6Me | 6OMe | 7a | 7b | 8 | 8Me | 10 | 10Me | 11 | 11OH | 12Me | 13 | 14a | 14b | 15 |
|---|---|---|---|---|---|---|---|---|---|---|---|---|---|---|---|---|---|---|---|---|---|---|
| 2 | • | ss | | s | | | ss | | | | | | | ss | | | | | | | | |
| 2Me | ss | • | ss | s | | | | | | | | | | | | | | | | | | m |
| 3 | | ss | • | | ss | ss | s | | | | | | | | | | | | | | | |
| 3OH | m | s | ss | • | ss | | | ww | | | | | | | | | | | | | | |
| 4 | | | ss | ss | • | | ss | ss | | | | | | | | | | | | | | |
| 4Me | | | ss | | ss | • | w | | | | | | | | | | | | | | | |
| 5 | ss | | | s | m | w | • | ss | s | | s | | | | s | | | | | | | |
| 6Me | | | | w | ss | | ss | • | ss | m | m | ww | | | | | | | | | | |
| 6OMe | | | | | | m | ss | ss | • | s | ww | m | ww | | | | | | | | | |
| 7a | | | | | | | | ss | ss | • | | | m | | | | | | | | | |
| 7b | | | | | | | ss | ss | ww | | • | | s | | ss | | | | | | | |
| 8 | | | | | | | | | m | | | • | | | | | | | | | | |
| 8Me | | | | | | | | | w | m | w | ss | • | | ss | s | | | | | | |
| 10 | ss | | | | | | | | | | | | | • | ss | | s | | | | | |
| 10Me | | | | | | | s | | | | s | | ss | ss | • | s | s | | | | | |
| 11 | | | | | | | | | | | | | s | | ss | • | | ss | | | | |
| 11OH | | | | | | | | | | | | | | s | s | ss | • | m | | s | | |
| 12Me | | | | | | | | | | | | | | | | ss | m | • | s | m | ss | |
| 13 | | | | | | | | | | | | | | | | | | ss | • | s | w | ss |
| 14a | | | | | | | | | | | | | | | | | s | m | | • | ss | ss |
| 14b | | | | | | | | | | | | | | | | ss | | | w | ss | • | |
| 15 | | m | | | | | | | | | | | | | | | s | s | | | | • |

ss - very strong, s - strong, m - medium, w - weak, ww - very weak, blue shading – most probably nOe signals of 6OMe, overlap with 10, green shading – key nOe's for conformational analysis

**Table SI2.** nOe interactions of compound **2** in $CDCl_3$ at 25 °C.

| | 2 | 2Me | 3 | 3OH | 4 | 4Me | 5 | 6Me | 6OMe | 7a | 7b | 8 | 8Me | 10 | 10Me | 11 | 11OH | 12Me | 13 | 14a | 14b | 15 |
|---|---|---|---|---|---|---|---|---|---|---|---|---|---|---|---|---|---|---|---|---|---|---|
| 2 | • | | | | | | ss | | | | | | | ss | | | | | | | | |
| 2Me | | • | ss | | w | | | | | | | | | | | | | | | | | w |
| 3 | | ss | • | | ss | ss | | | | | | | | | | | | | | | | |
| 3OH | | | | • | | | | | | | | | | | | | | | | | | |
| 4 | | | ss | | • | | ss | ss | | | | | | | | | | | | | | |
| 4Me | | | ss | | | • | | | s | w | | | | | | | | | | | | |
| 5 | ss | | | | ss | | • | ss | | | s | | | ss | | | | | | | | |
| 6Me | | | | | ss | | ss | • | ss | s | s | | | | | | | | | | | |
| 6OMe | | | | | | | | ss | • | ss | | m | | | | | | | | | | |
| 7a | | | | | | | | ss | ss | • | | | s | | | | | | | | | |
| 7b | | | | | m | | s | s | | | • | | m | | ss | | | | | | | |
| 8 | | | | | | | | | | | | • | ss | | | | | | | | | |
| 8Me | | | | | | | | | | m | m | ss | • | | ss | m | | m | | | | |
| 10 | ss | | | | | | ss | | | ww | | | | • | | s | | | | | | |
| 10Me | | | | | | | | | | m | ss | ww | ss | | • | ss | | | | | | |
| 11 | | | | | | | | | | | | | s | | ss | • | | ss | | | | |
| 11OH | | | | | | | | | | | | | | | | | • | | | | | |
| 12Me | | | | | | | | | | | | | | | | ss | | • | s | m | ss | |
| 13 | | | | | | | | | | | | | | | | | | ss | • | | | m |
| 14a | | | | | | | | | | | | | | | | | | s | | • | | |
| 14b | | | | | | | | | | | | | | | | | | ss | | | • | |
| 15 | | w | | | | | | | | | | | | | | | | | ss | | s | • |

ss - very strong, s - strong, m - medium, w - weak, ww - very weak

**Table SI3.** nOe interactions of compound **3** in $CDCl_3$ at 25 °C.

| | 2 | 2M | 3 | 3OH | 4 | 4M | 5 | 6Me | 6OMe | 7a | 7b | 8 | 8M | 10 | 10Me | 11 | 11OH | 12Me | 13 | 14a | 14b | 15 |
|---|---|---|---|---|---|---|---|---|---|---|---|---|---|---|---|---|---|---|---|---|---|---|
| 2 | ● | | m | | | | ss | | | | | | | | | | | | | | | |
| 2M | | ● | | | | | | | | | | | | | | | | | | | | |
| 3 | m | s | ● | | s | m | | | | | | | | | | | | | | | | |
| 3OH | | | | ● | | | | | | | | | | | | | | | | | | |
| 4 | | | ss | | ● | | | s | w | | | | | | | | | | | | | |
| 4M | | | m | | | ● | ww | | w | | | | | | | | | | | | | |
| 5 | m | | | | s | | ● | ss | | | w | | | | ss | | | | | | | |
| 6Me | | | | | s | | ss | ● | ss | m | | s | | | | | | | | | | |
| 6OMe | | | | | w | ww | | ss | ● | m | | ww | | | | | | | | | | |
| 7a | | | | | | | | s | s | ● | | | m | | | | | | | | | |
| 7b | | | | | m | | s | s | | | ● | | m | | s | | | | | | | |
| 8 | | | | | | | | s | w | | | ● | m | | | | | | | | | |
| 8Me | | | | | | | | | | m | w | s | ● | | ss | | | | | | | |
| 10 | | | | | | | | | | | | | | ● | | | | | | | | |
| 10Me | ww | | | | | | ss | | | | m | | s | | ● | s | | | | | | |
| 11 | | | | | | | | | | | | | | | m | ● | s | | | | ww | |
| 11OH | | | | | | | | | | | | | | | | | ● | | | | | |
| 12Me | | | | | | | | | | | | | | | s | | | ● | m | | | |
| 13 | | | | | | | | | | | | | | | | | | m | ● | ww | w | w |
| 14a | | | | | | | | | | | | | | | | | | | m | ● | | |
| 14b | | | | | | | | | | | | | | | | m | | | | | ● | |
| 15 | | | | | | | | | | | | | | | | w | | | m | s | s | ● |

ss - very strong, s - strong, m - medium, w - weak, ww - very weak, blue shading – overlap 4 and 8

**Table SI4.** nOe interactions of compound **4** in DMSO-$d_6$ at 25 °C.

| | 2 | 2Me | 3 | 3OH | 4 | 4Me | 5 | 6Me | 6OMe | 7a | 7b | 8 | 8Me | 10 | 10Me | 11 | 12Me | 13 | 14a | 14b | 15 |
|---|---|---|---|---|---|---|---|---|---|---|---|---|---|---|---|---|---|---|---|---|---|
| 2 | • | s | w | | | | s | | | | | | | | | | | | | | |
| 2Me | s | • | m | | | | | | | | | | | | | | | | | | w |
| 3 | w | s | • | | m | s | m | | | | | | | | | | | | | | |
| 3OH | | | | • | | | | | | | | | | | | | | | | | |
| 4 | | | m | | • | s | s | | m | | | | | | | | | | | | |
| 4Me | | | m | | s | • | w | | w | | s | | | | | | | m | | | |
| 5 | m | | | | s | | • | s | w | | | | | | | | | | | | |
| 6Me | | | | | | | s | • | s | m | | s | | | | | | | | | |
| 6OMe | | | | | w | w | w | s | • | m | w | | | | | | | | | | |
| 7a | | | | | | | | m | m | • | s | m | s | | | | | | | | |
| 7b | | | | | | s | | | w | s | • | | m | | | | | | | | |
| 8 | | | | | | | | s | | m | | • | s | | s | | | | | | |
| 8Me | | | | | | | | | | m | s | m | • | | m | w | s | | | | |
| 10 | | | | | | | | | | | | | | • | | | | | | | |
| 10Me | | | | | | | | | | | | m | | | • | s | s | | | | |
| 11 | | | | | | | | | | | | | | | s | • | s | | | s | |
| 12Me | | | | | | | | | | | | | s | | | s | • | s | m | | |
| 13 | | | | | | m | | | | | | | | | | | s | • | | | m |
| 14a | | | | | | | | | | | | | | | | | | | • | s | s |
| 14b | | | | | | | | | | | | | | | | s | | | s | • | |
| 15 | | w | | | | | | | | | | | | | | | w | m | s | | • |

s - strong, m - medium, w - weak, blue shading – signals used in molecular modelling calculations

**Table SI5.** Proton vicinal coupling constants ($^3J$) for compound **4** in DMSO-$d_6$ at 25 °C with corresponding angle constraints.

| Protons | $^3$J/Hz | Angle constrains | |
|---|---|---|---|
| | | **0° ≤ α ≤ 90°** | **90° ≤ α ≤ 180°** |
| 2, 3 | 10.3 | | 180 |
| 3, 4 | 3.0 | 51 | 125 |
| 4, 5 | 2.0 | 60 | 120 |
| 7a, 8 | 3.7 | 46 | 130 |
| 7b, 8 | 12.7 | | 180 |
| 13, 14a | 2.4 | 56 | 122 |
| 13, 14b | 11.3 | | 180 |

## LC–HRMS ANALYSIS

LC–HRMS analysis of compound **2** revealed a chromatographic peak at $t_R$ = 12.6 min to be the spiroketal. HRMS results confirm the proposed structure based on three signals: protonated molecule $(M + H)^+$, ammonium adduct $(M + NH_4)^+$ and ammonium adduct of dimmer molecule $(2M + NH_4)^+$ (Figure SI1 and Table SI6). According to these results, fragmentation scheme was proposed (Figure SI2). Furthermore, H/D exchange experiment confirmed two exchangeable protons.

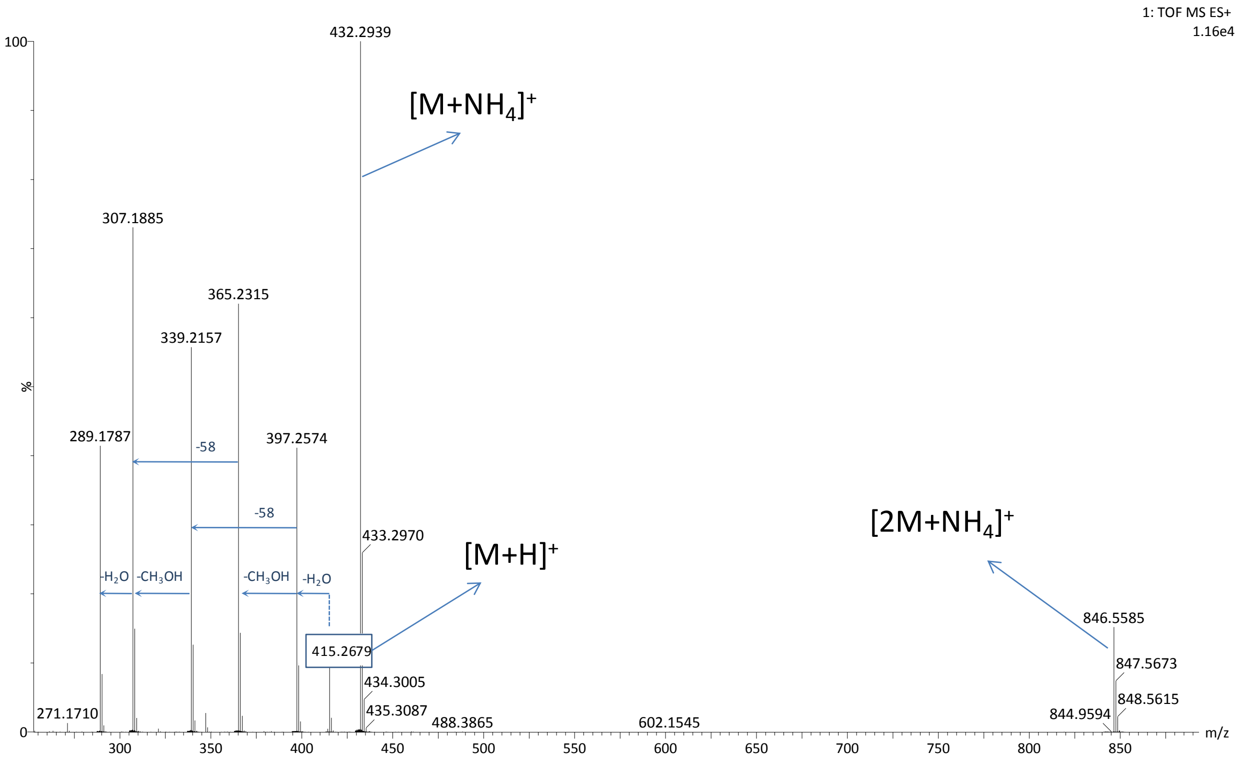


**Figure SI1.** Full scan HRMS spectrum and signal assignment of compound **2**.

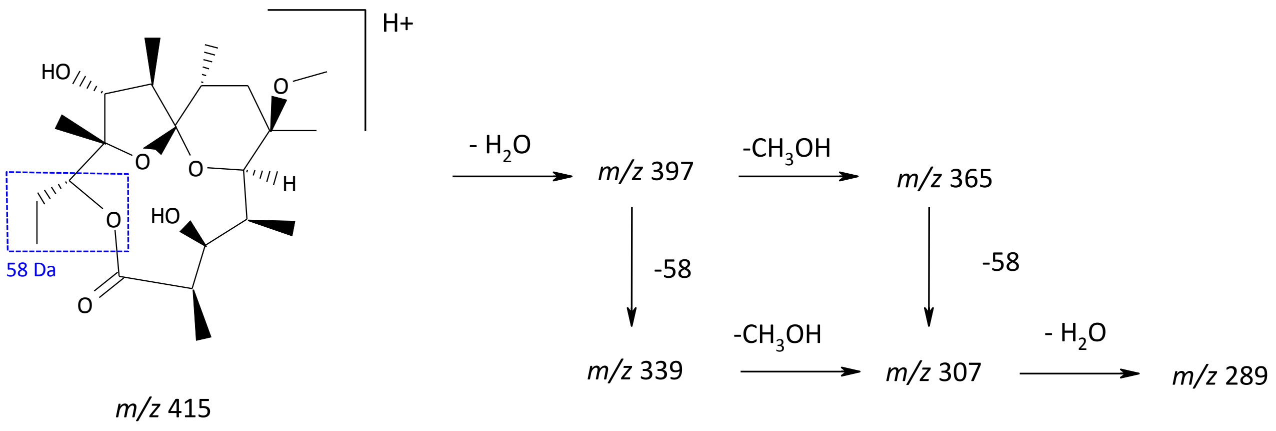


**Figure SI2.** Proposed fragmentation scheme for a compound **2**; elimination of neutral fragment 58 Da corresponds to part of molecule marked with box on scheme.

**Table SI6.** Elemental composition results for the most significant signals in MS spectra of compound **2**.

| Ion | Molecular formula | Calculated mass | Measured mass | Error (ppm) |
|---|---|---|---|---|
| $[M+H]^+$ | C22H39O7 | 415.2696 | 415.2679 | -4.1 |
| $[M+NH_4]^+$ | C22H42NO7 | 432.2961 | 432.2939 | -5.1 |
| $[2M+NH_4]^+$ | C44H80NO14 | 846.5579 | 846.5585 | 0.7 |

**Table SI7.** Accurate mass measurements for determination of molecular formula of **2–4.**

| Compound | Molecular formula | Calculated mass | Measured mass | Error (ppm) |
|---|---|---|---|---|
| **2** | C22 H38 O7 Na | 437.2515 | 437.2504 | -2.5 |
| **3** | C22 H37 O6 | 397.2590 | 397.2586 | -1.0 |
| **4** | C22 H37 O6 | 397.2590 | 397.2588 | -0.5 |

## REACTION KINETICS

The rate constants were calculated using the Microsoft Excel 2013 (32 bit) for Microsoft Windows 8.1 (64 bit) with Solver add-in. The time dependence of [A], [B] and [C] concentrations for reaction scheme presented in Eq. 1 is given by the following equations (see the reference 32):

$$[\mathrm{A}] = [\mathrm{A}]_0 \exp(-k_1 t)$$

$$[\mathrm{B}] = [\mathrm{B}]_0 + \alpha_2 + \frac{1}{k_2 + k_3 - k_1}\left\{[\mathrm{A}]_0 (k_1 - k_3)\exp(-k_1 t) - [\alpha_2 k_2 - \alpha_3 (k_3 - k_1)]\exp(-(k_2 + k_3)t)\right\}$$

$$[\mathrm{C}] = [\mathrm{C}]_0 + \alpha_3 + \frac{1}{k_2 + k_3 - k_1}\left\{[\mathrm{A}]_0 k_2 \exp(-k_1 t) - [\alpha_2 k_2 - \alpha_3 (k_3 - k_1)]\exp(-(k_2 + k_3)t)\right\}$$

where $\alpha_1 = [\mathrm{A}]_\infty - [\mathrm{A}]_0$, $\alpha_2 = [\mathrm{B}]_\infty - [\mathrm{B}]_0$ and $\alpha_3 = [\mathrm{C}]_\infty - [\mathrm{C}]_0$, while subscripts 0 and $\infty$ present the values of corresponding concentrations at times $t = 0$ and $t = \infty$. The experimental data was fitted by non-linear least squares fit method i.e. simulated data vs. experimental data, by varying the constants $k_1$, $k_2$ and $k_3$, with $R^2$ as the criterion for the goodness of fit. The algorithm used by Solver to find the optimal solutions was the GRG Nonlinear Solving Method for nonlinear optimization (uses the Generalized Reduced Gradient (GRG2) code), followed by the Evolutionary Solving Method for non-smooth optimization which uses a variety of genetic algorithm and local search methods. The lines on Figure 3 present the best fit obtained for the concentration values calculated as stated above and the rate constants are determined from these functions.
