# Supplementary material for: Structure and conformational analysis of spiroketals from 6-O-methyl-9(E)-hydroxyiminoerythronolide A
Source: Beilstein J Org Chem. 2015 Aug 19;11:1447–57. doi: 10.3762/bjoc.11.157 (PMC4578343; doi:10.3762/bjoc.11.157)
Supplement: File 3 — NMR spectra of compounds 2–4. [file Beilstein_J_Org_Chem-11-1447-s003.zip › NMRspectra/cpd234_HMBC_cdcl3.pdf]

Compounds 2, 3 and 4 in the mixture CDCl3

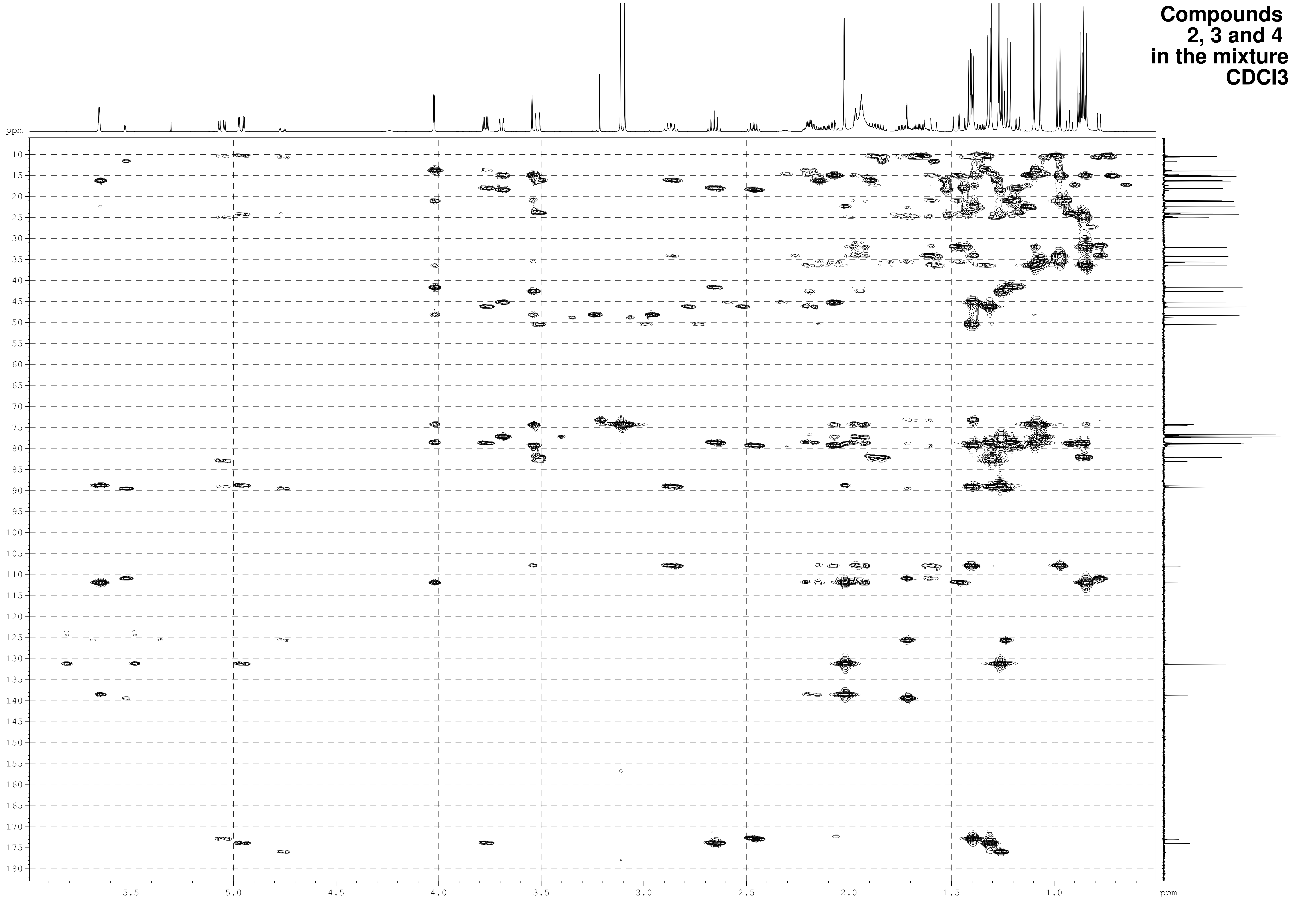

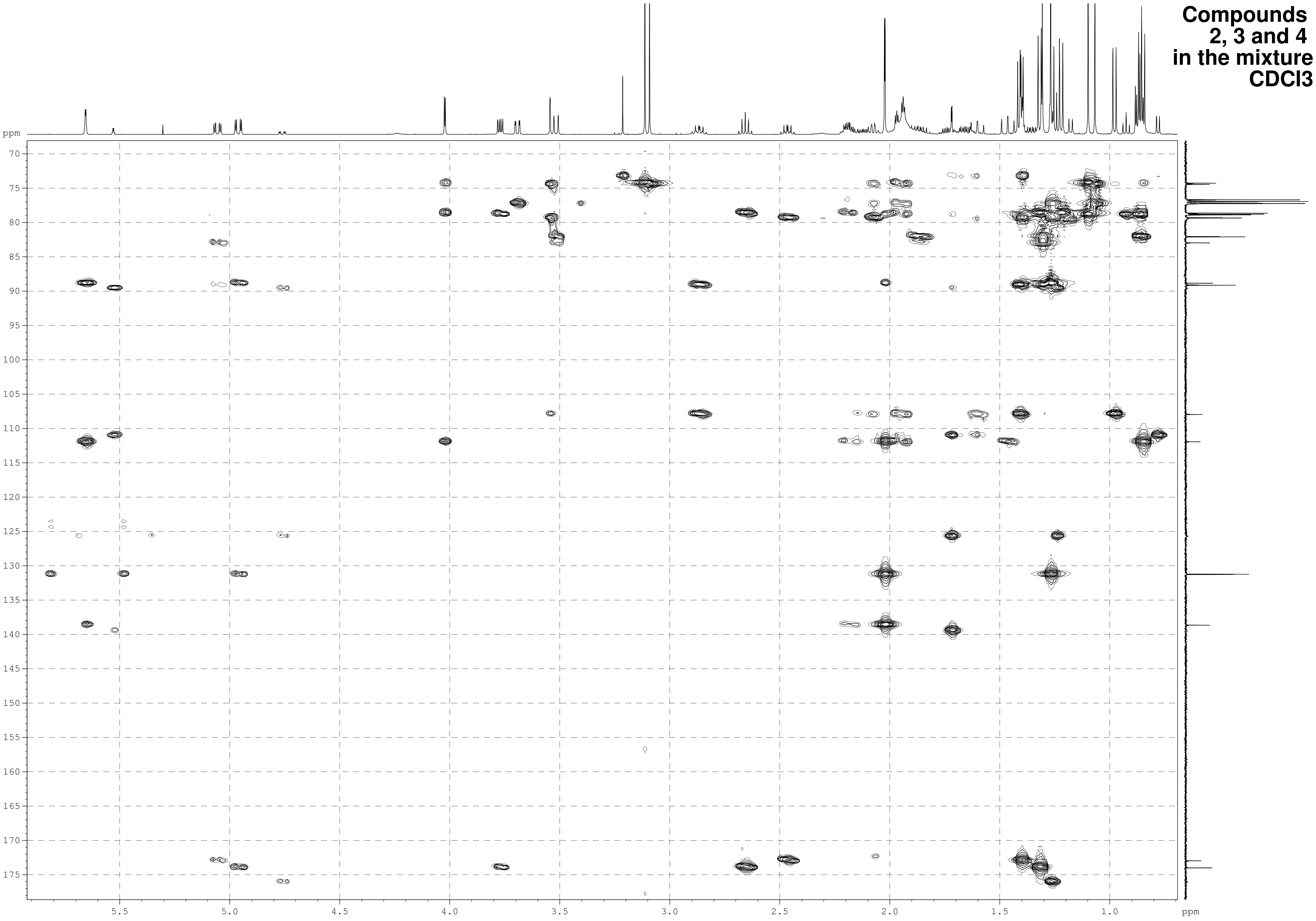
Compounds
2, 3 and 4
in the mixture
CDCl3
ppm
ppm

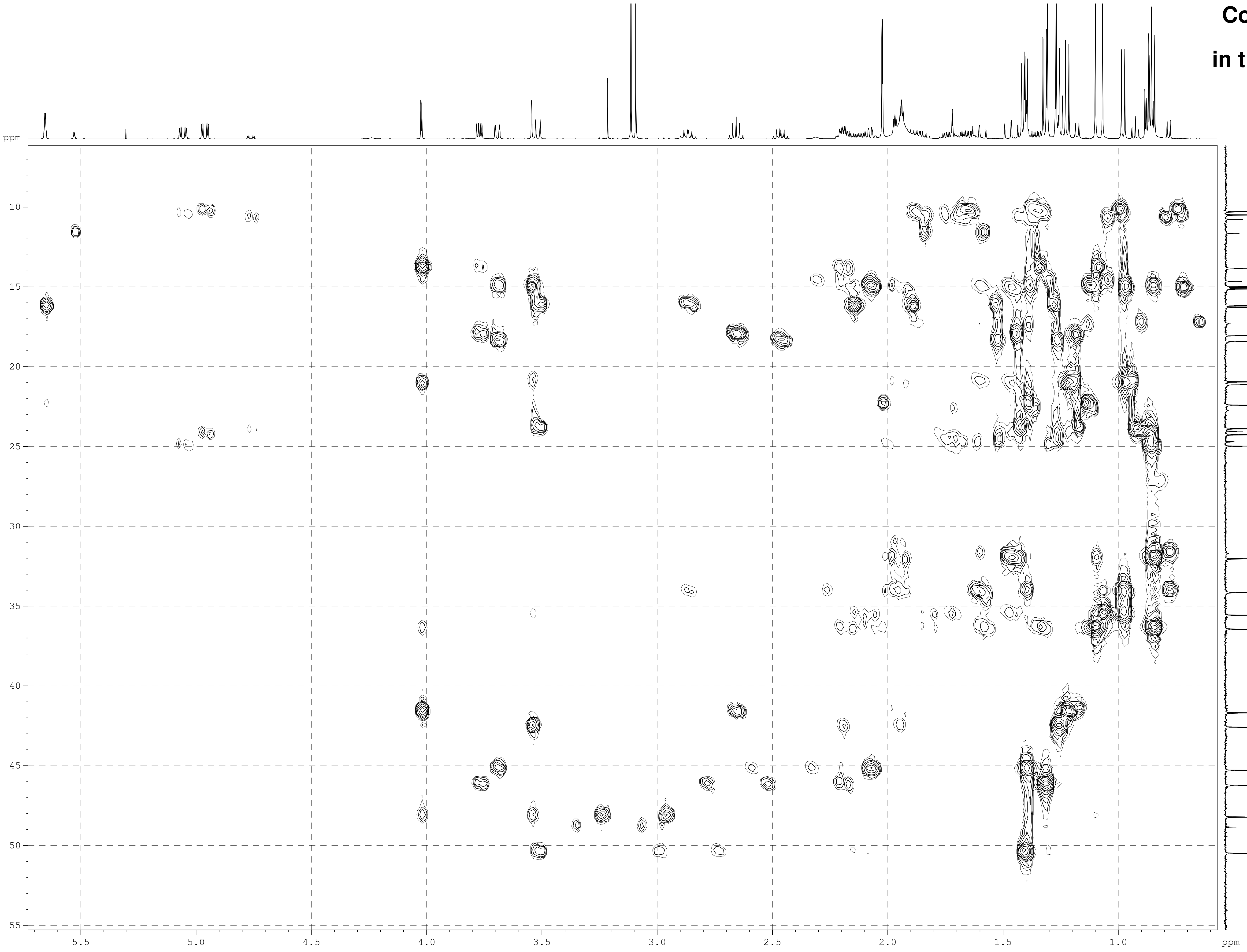

Compounds
2, 3 and 4
in the mixture
CDCl3
