# Supplementary figures and images for: Structure and conformational analysis of spiroketals from 6-O-methyl-9(E)-hydroxyiminoerythronolide A
Source: Beilstein J Org Chem. 2015 Aug 19;11:1447–57. doi: 10.3762/bjoc.11.157 (PMC4578343; doi:10.3762/bjoc.11.157)

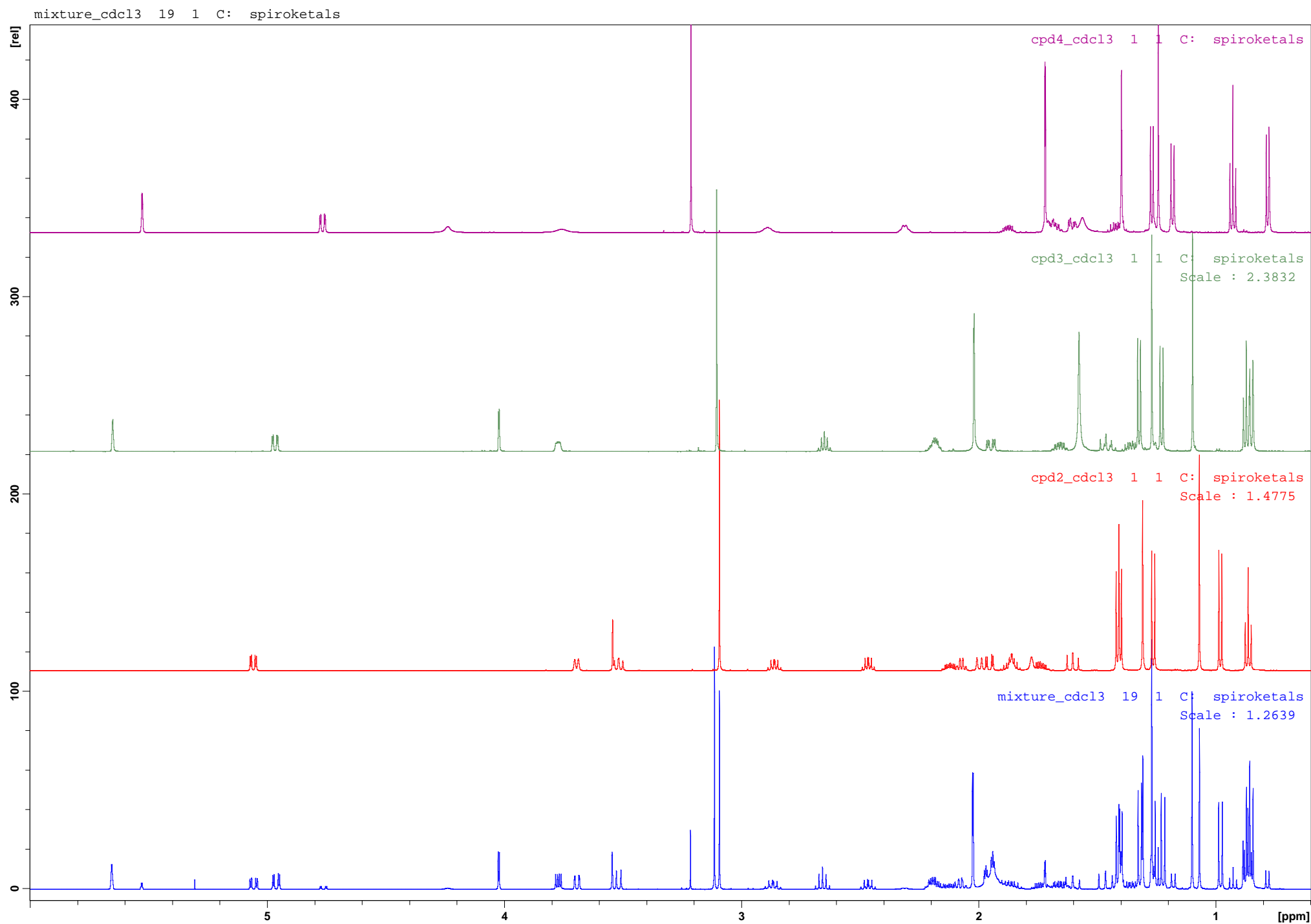

Supplement: File 3 — NMR spectra of compounds 2–4. [file Beilstein_J_Org_Chem-11-1447-s003.zip › NMRspectra/comparison.pdf]

Compounds 2, 3 and 4 in the mixture  
CDCl<sub>3</sub>

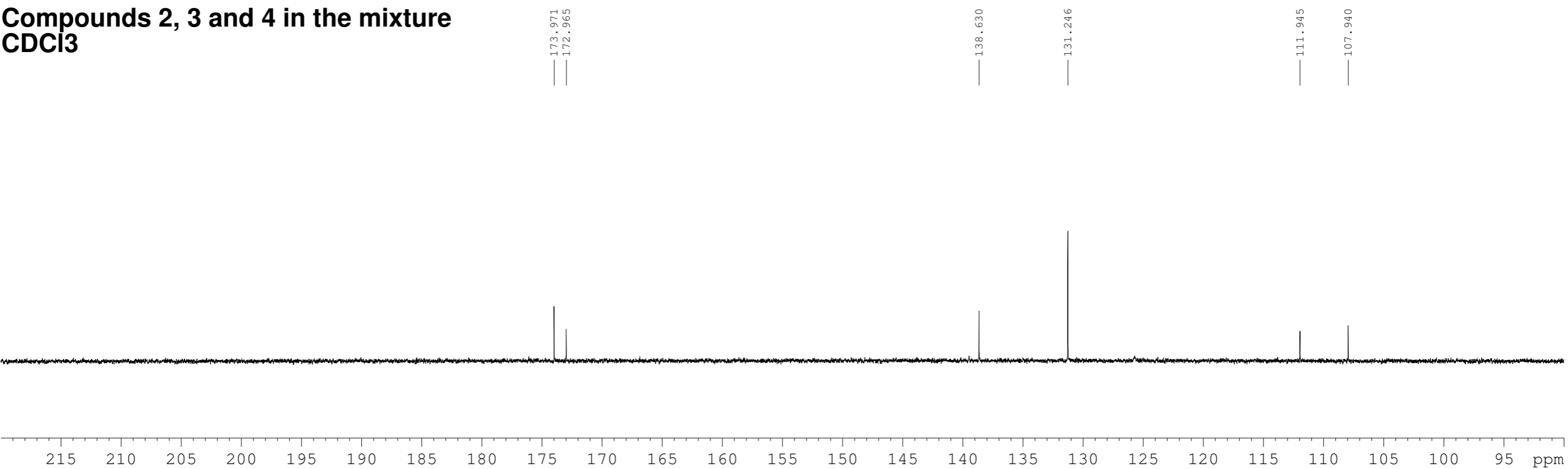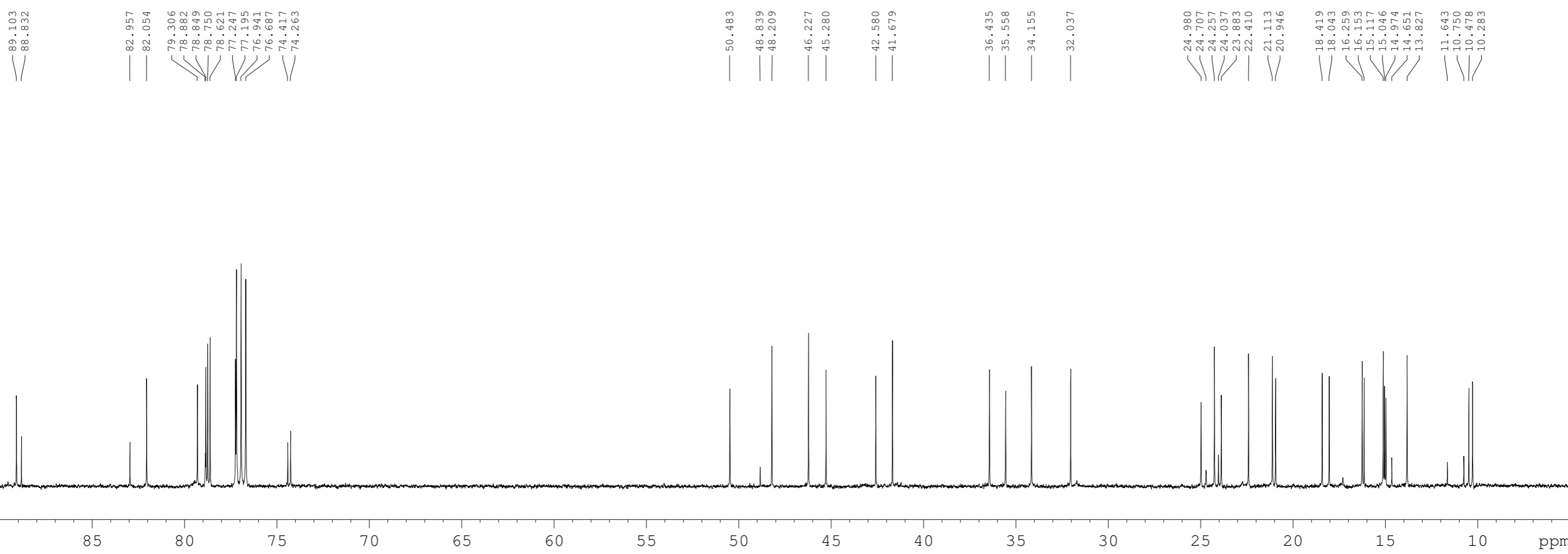

Supplement: File 3 — NMR spectra of compounds 2–4. [file Beilstein_J_Org_Chem-11-1447-s003.zip › NMRspectra/cpd234_13C_cdcl3.pdf]

### Compounds 2, 3 and 4 in the mixture CDCl<sub>3</sub>

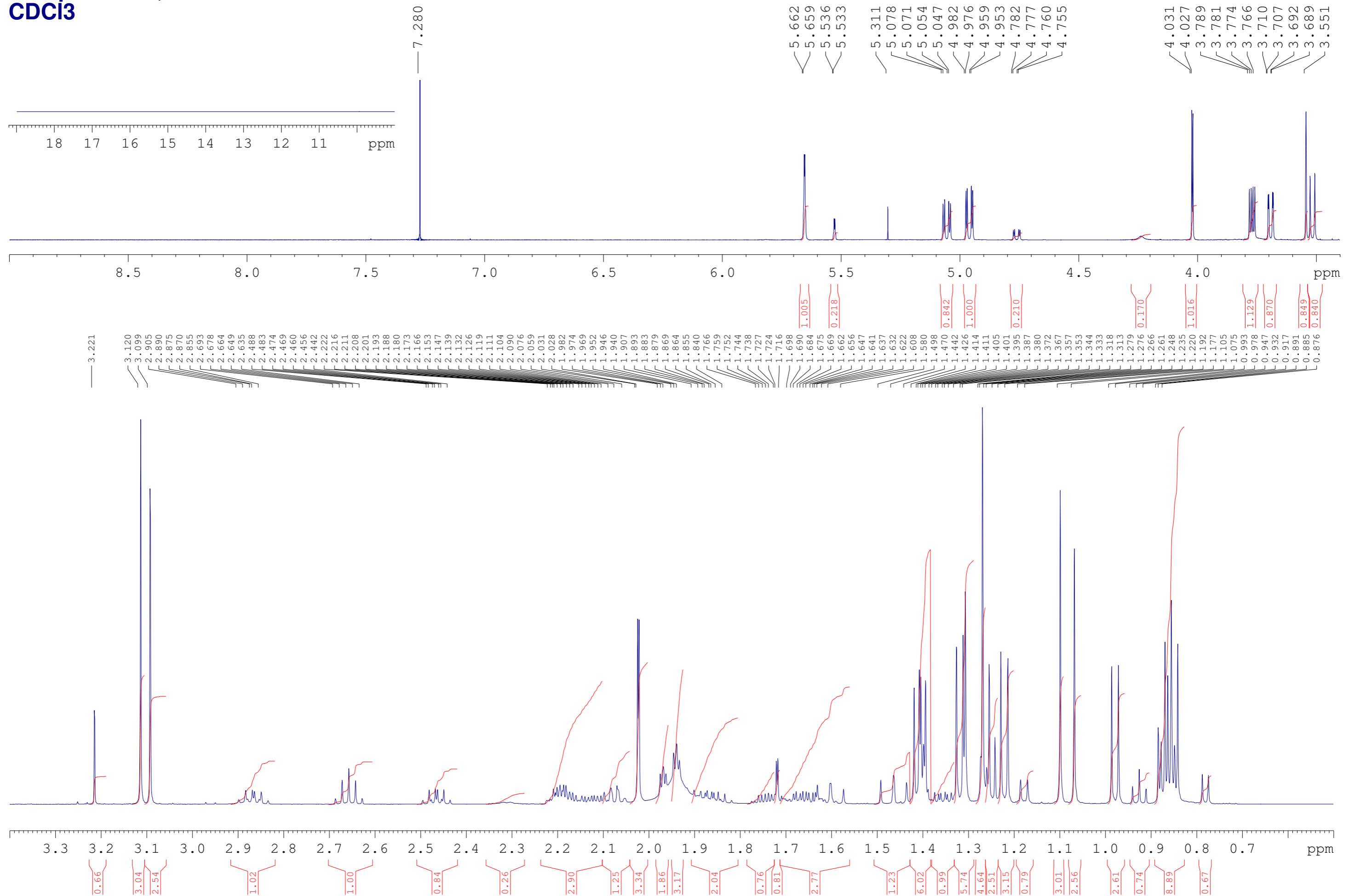

Supplement: File 3 — NMR spectra of compounds 2–4. [file Beilstein_J_Org_Chem-11-1447-s003.zip › NMRspectra/cpd234_1H_cdcl3.pdf]

**Compounds  
2, 3 and 4  
in the mixture  
CDCl<sub>3</sub>**

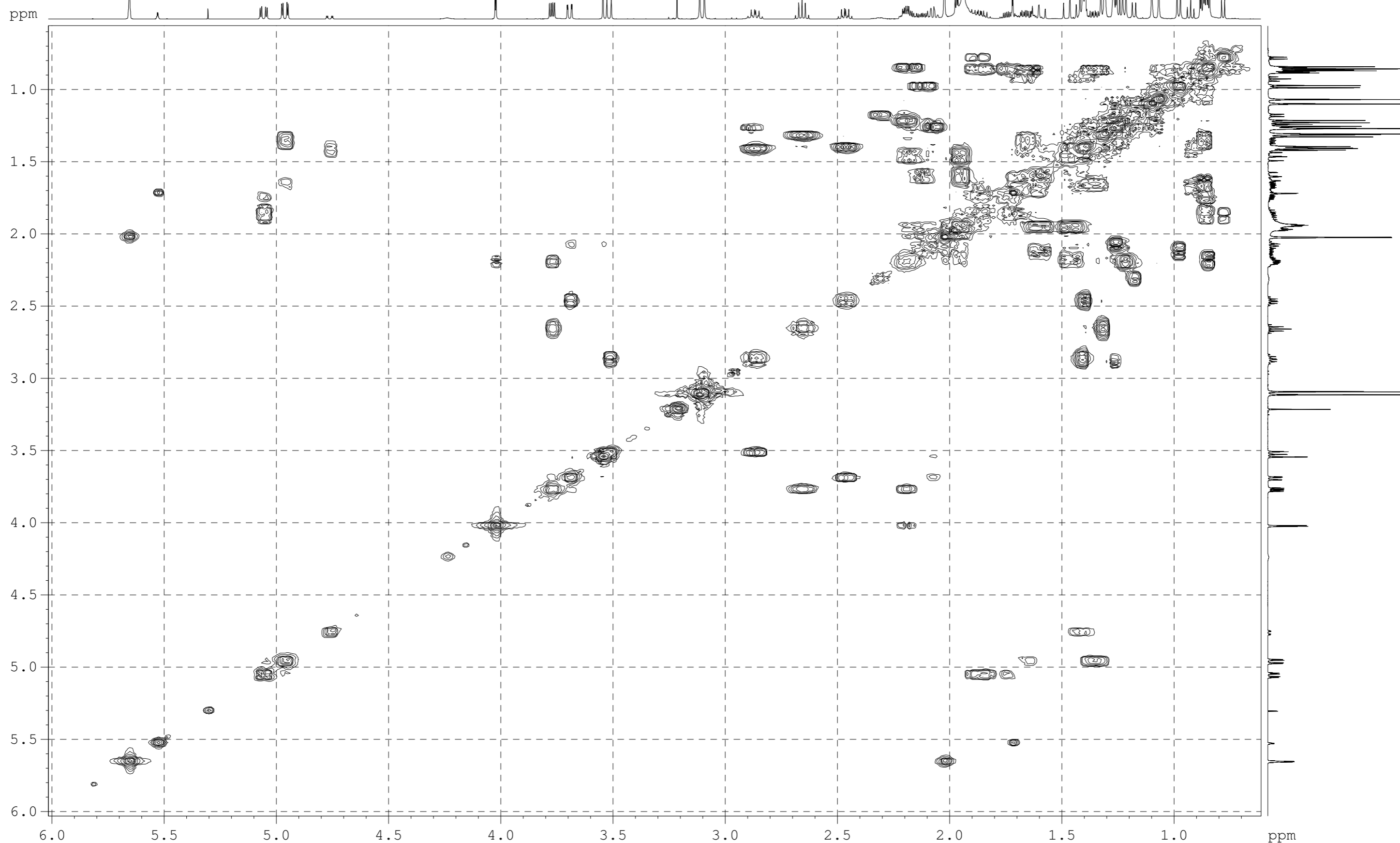

Supplement: File 3 — NMR spectra of compounds 2–4. [file Beilstein_J_Org_Chem-11-1447-s003.zip › NMRspectra/cpd234_COSY_cdcl3.pdf]

**Compounds  
2, 3 and 4  
in the mixture  
CDCl<sub>3</sub>**

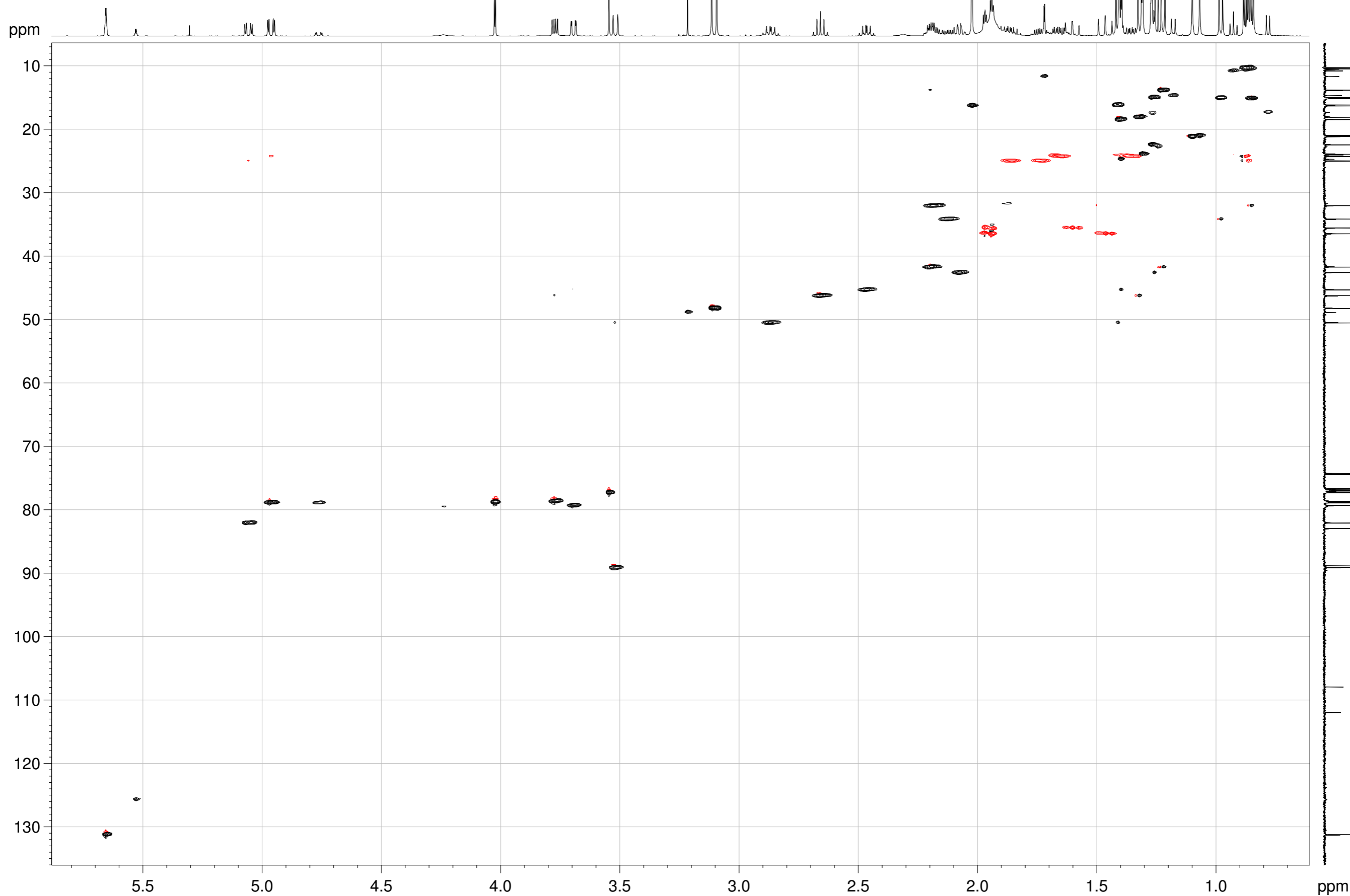

**Compounds  
2, 3 and 4  
in the mixture  
CDCl<sub>3</sub>**

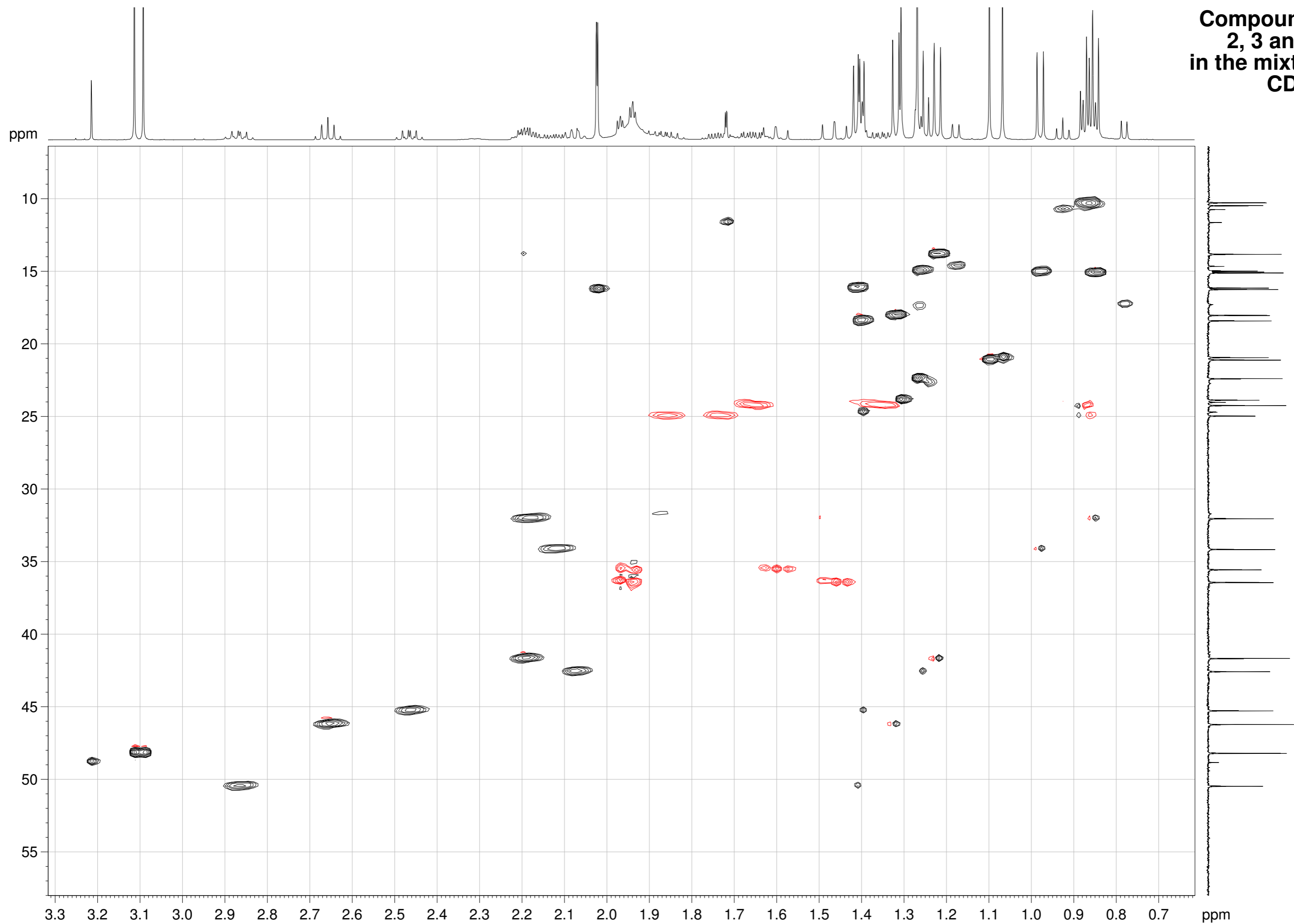

Supplement: File 3 — NMR spectra of compounds 2–4. [file Beilstein_J_Org_Chem-11-1447-s003.zip › NMRspectra/cpd234_HSQC_cdcl3.pdf]

Compound 2  
DMSO

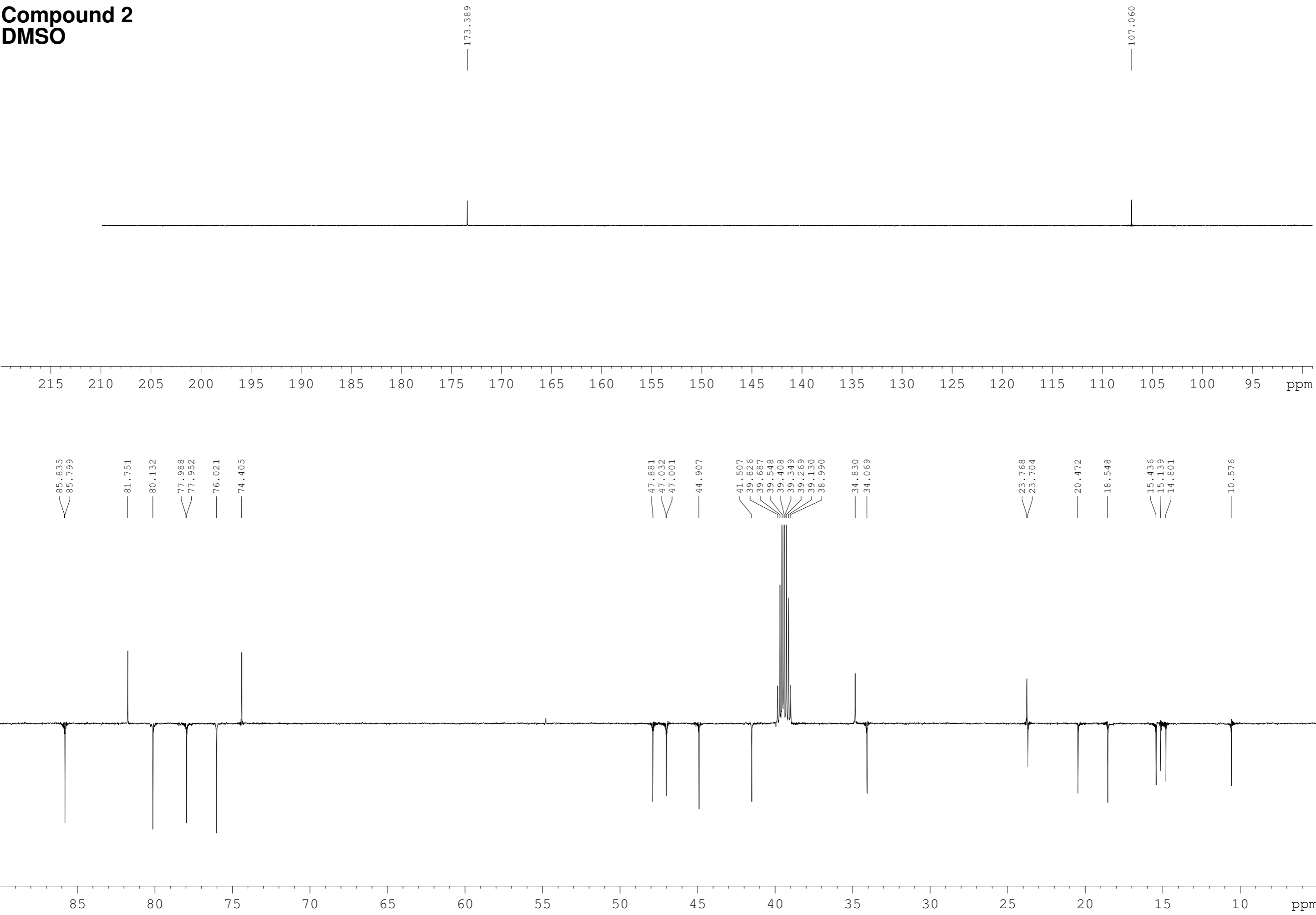

Supplement: File 3 — NMR spectra of compounds 2–4. [file Beilstein_J_Org_Chem-11-1447-s003.zip › NMRspectra/cpd2_13C_dmso.pdf]

Compound 2  
CDCl3

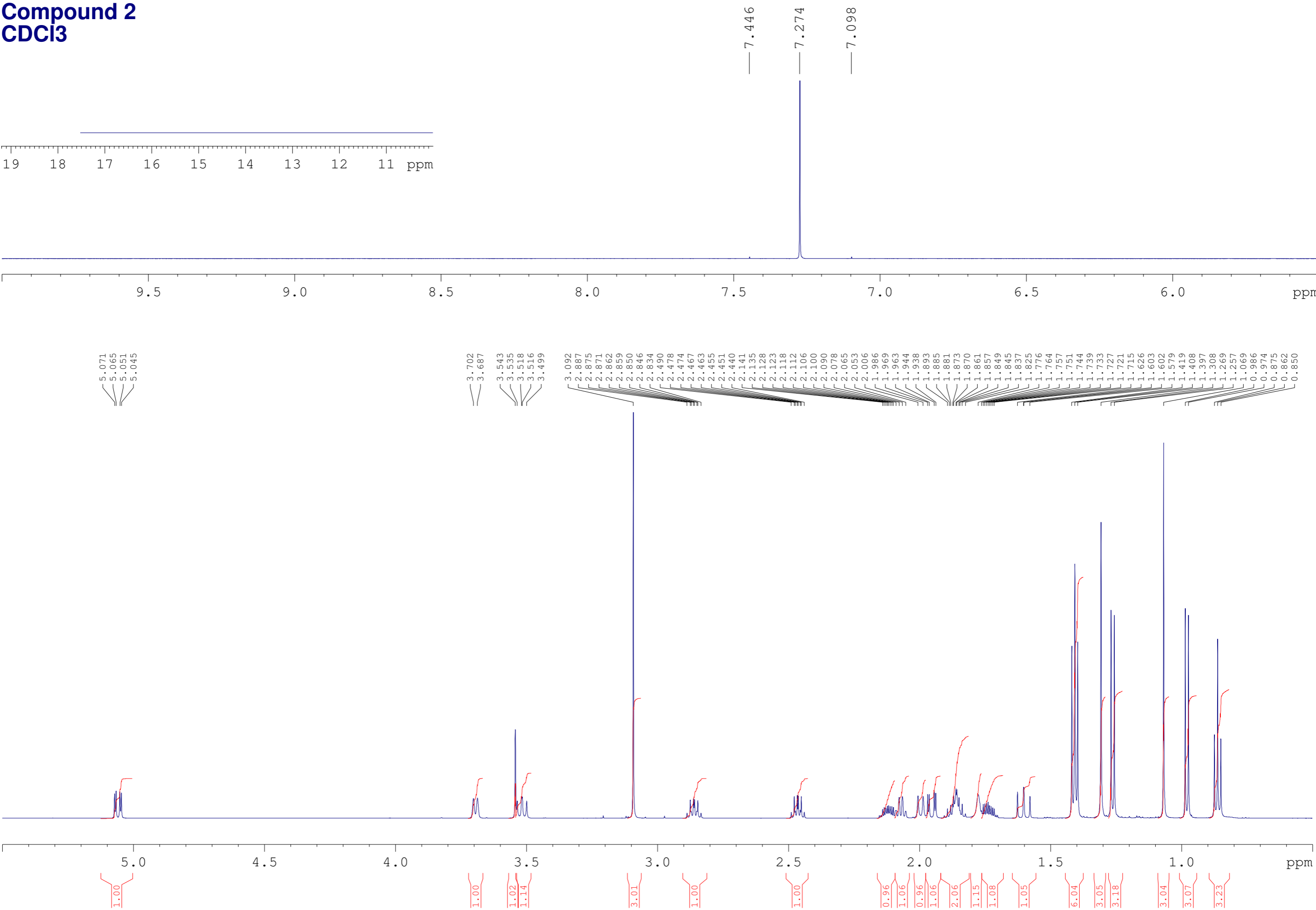

Supplement: File 3 — NMR spectra of compounds 2–4. [file Beilstein_J_Org_Chem-11-1447-s003.zip › NMRspectra/cpd2_1H_cdcl3.pdf]

Compound 2  
DMSO

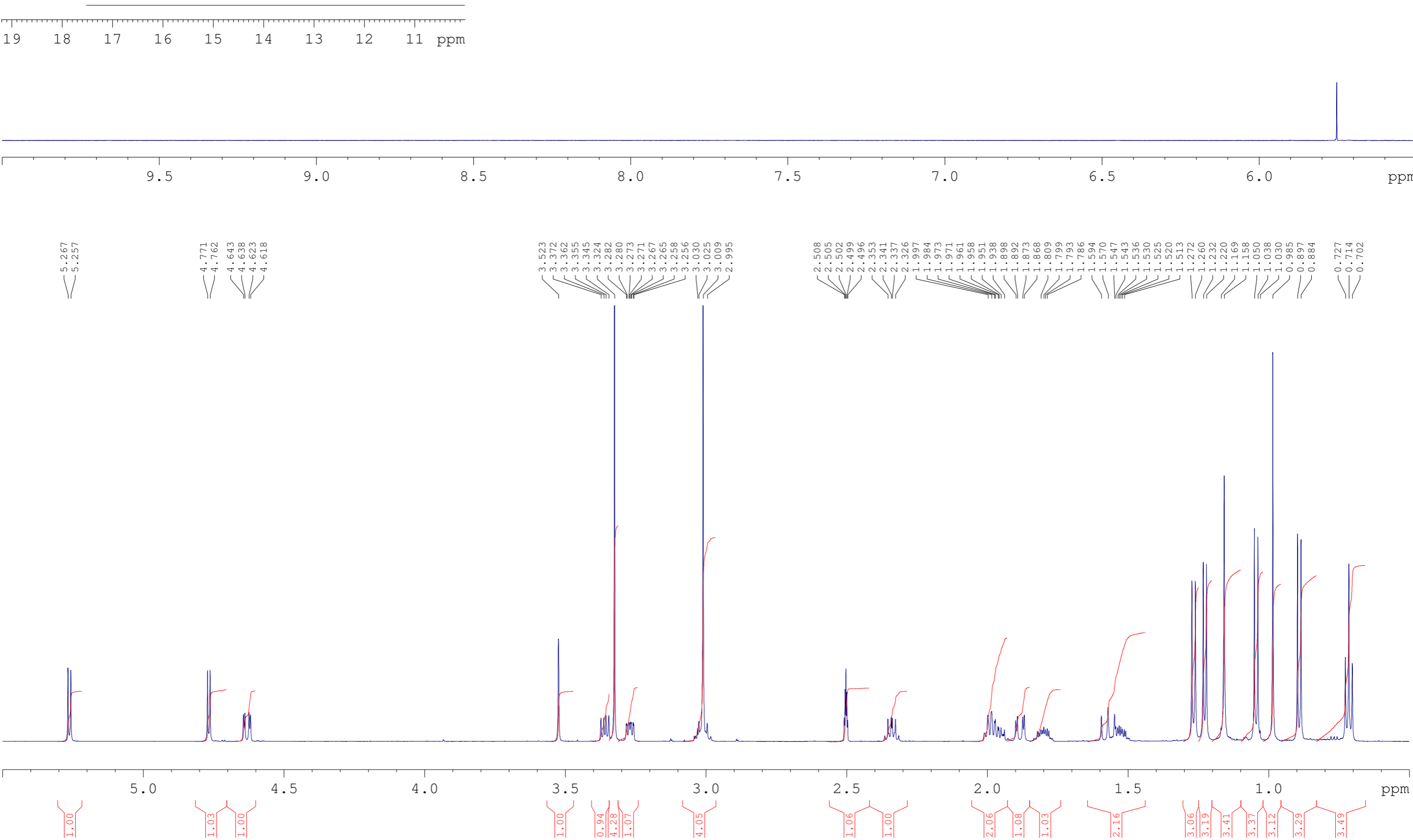

Supplement: File 3 — NMR spectra of compounds 2–4. [file Beilstein_J_Org_Chem-11-1447-s003.zip › NMRspectra/cpd2_1H_dmso.pdf]

Compound 2  
DMSO

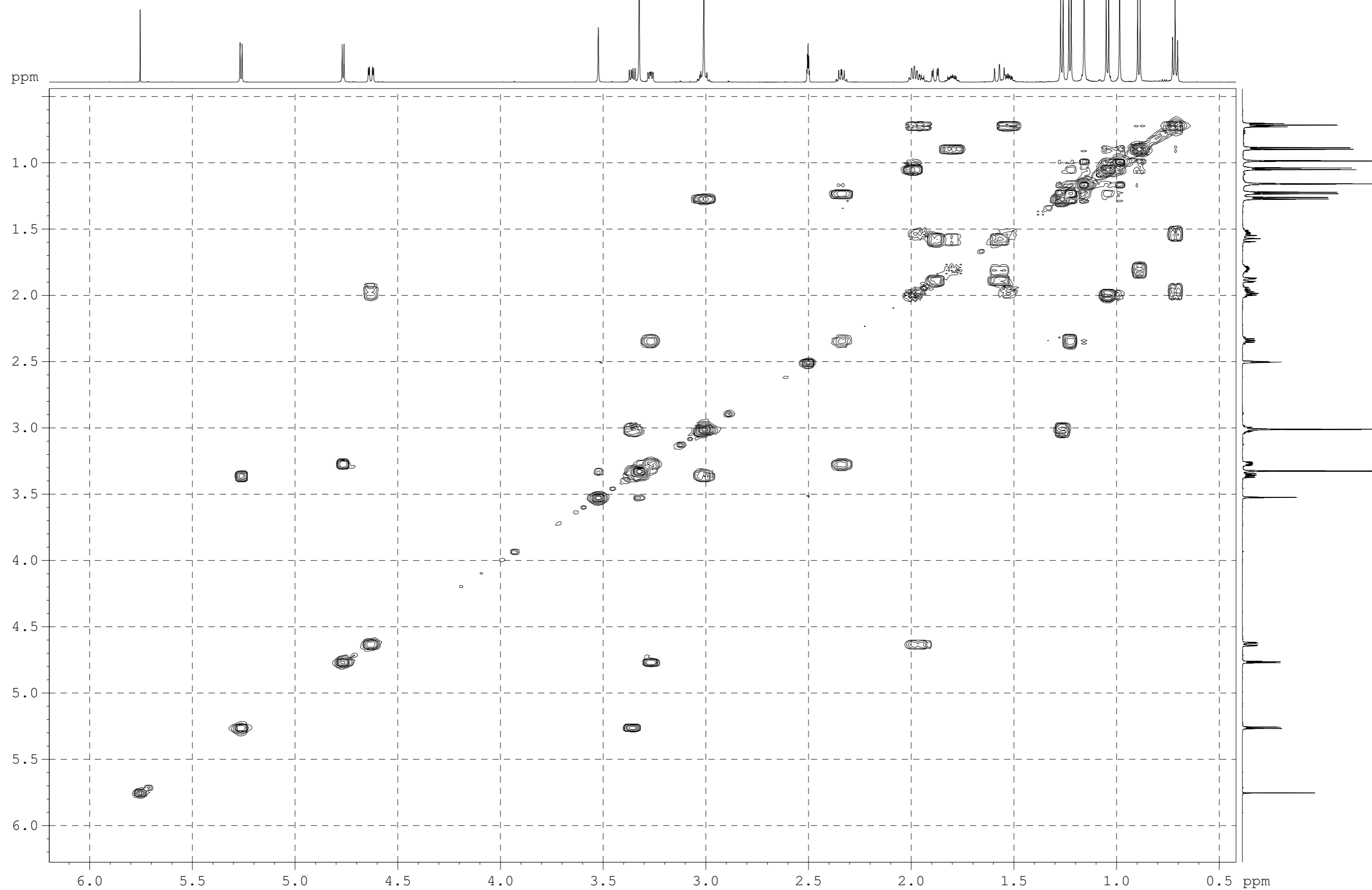

Supplement: File 3 — NMR spectra of compounds 2–4. [file Beilstein_J_Org_Chem-11-1447-s003.zip › NMRspectra/cpd2_COSY_dmso.pdf]

Compound 2  
DMSO

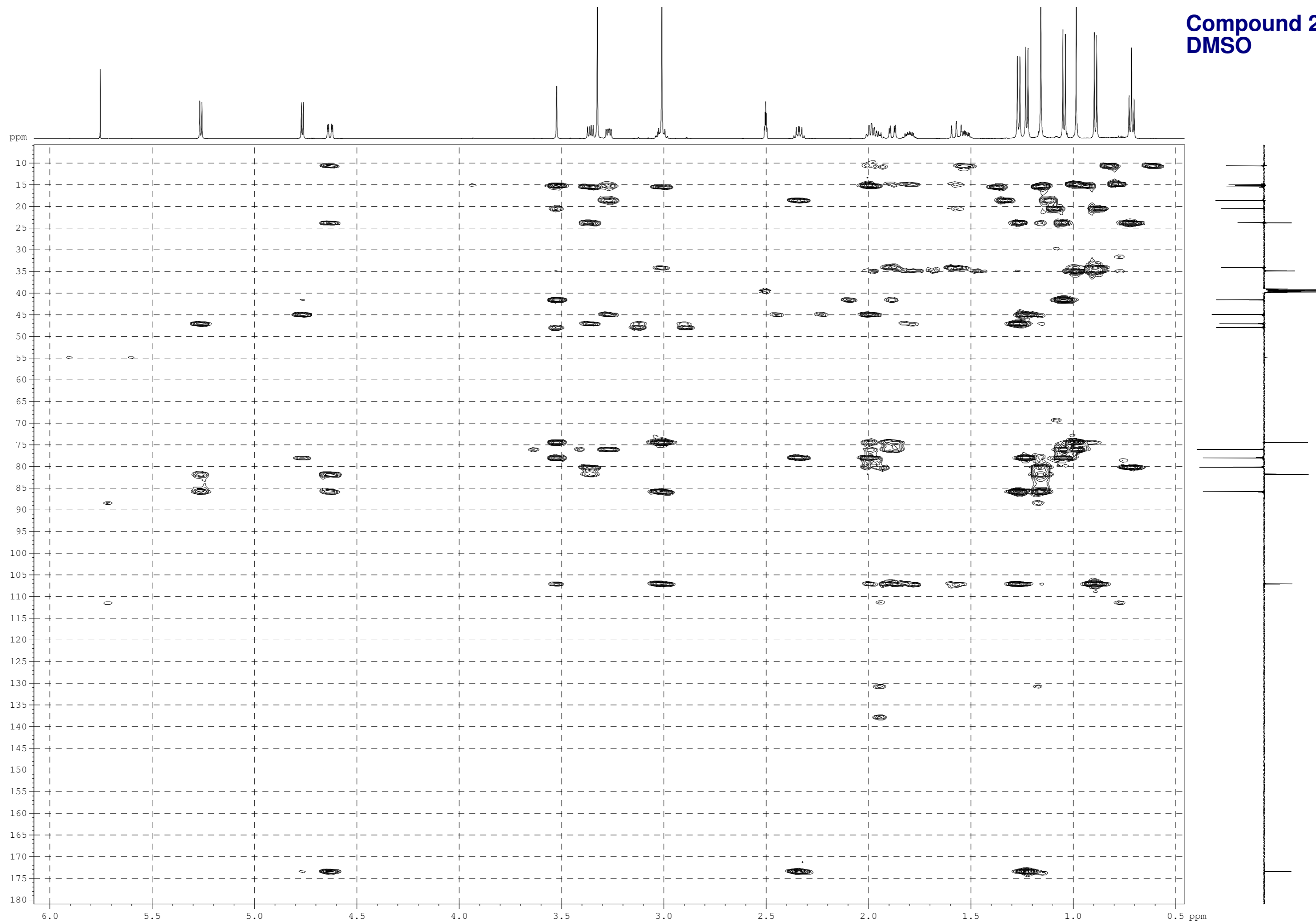

Supplement: File 3 — NMR spectra of compounds 2–4. [file Beilstein_J_Org_Chem-11-1447-s003.zip › NMRspectra/cpd2_HMBC_dmso.pdf]

Compound 2  
DMSO

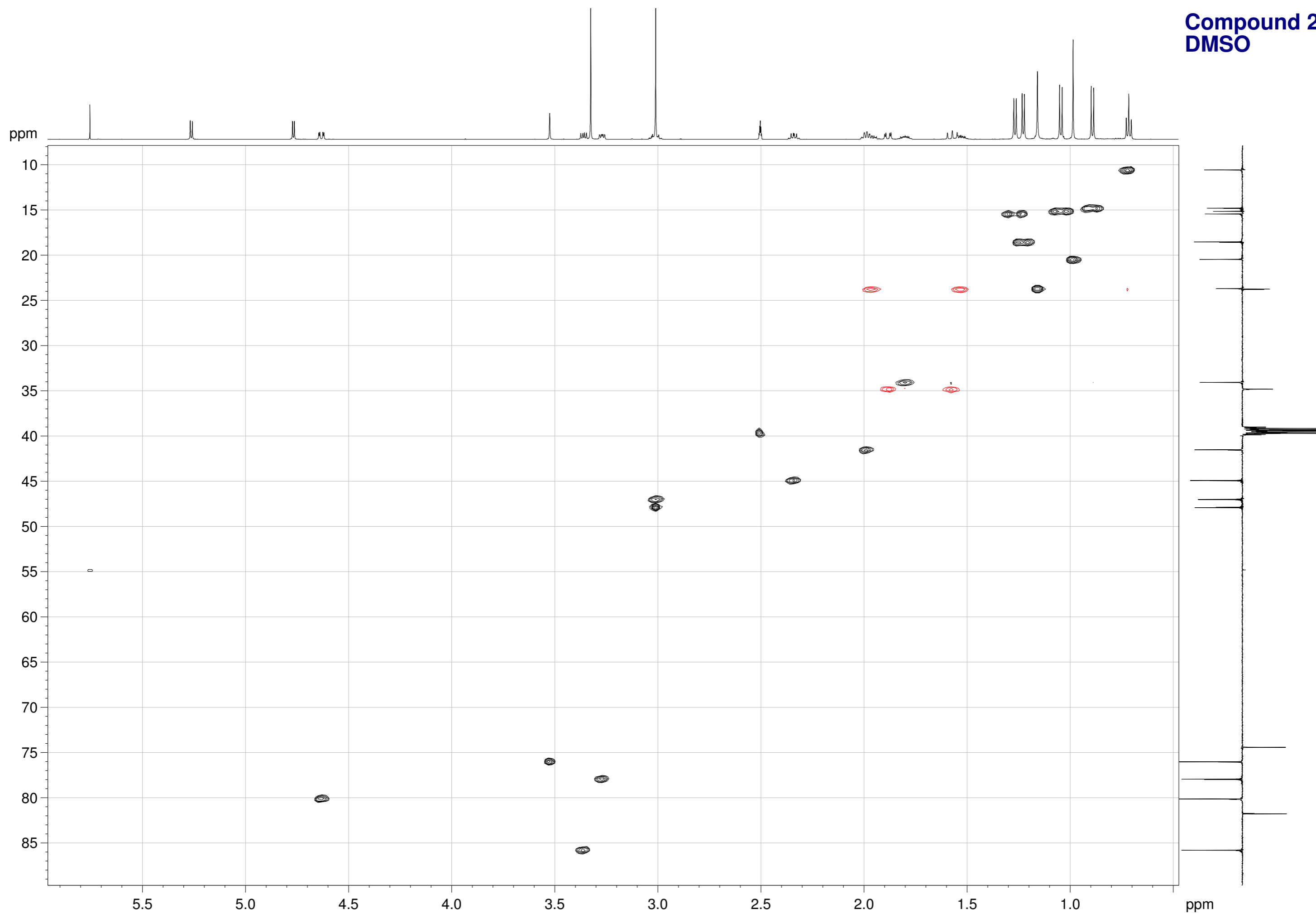

Supplement: File 3 — NMR spectra of compounds 2–4. [file Beilstein_J_Org_Chem-11-1447-s003.zip › NMRspectra/cpd2_HSQC_dmso.pdf]

Compound 2  
CDCl<sub>3</sub>

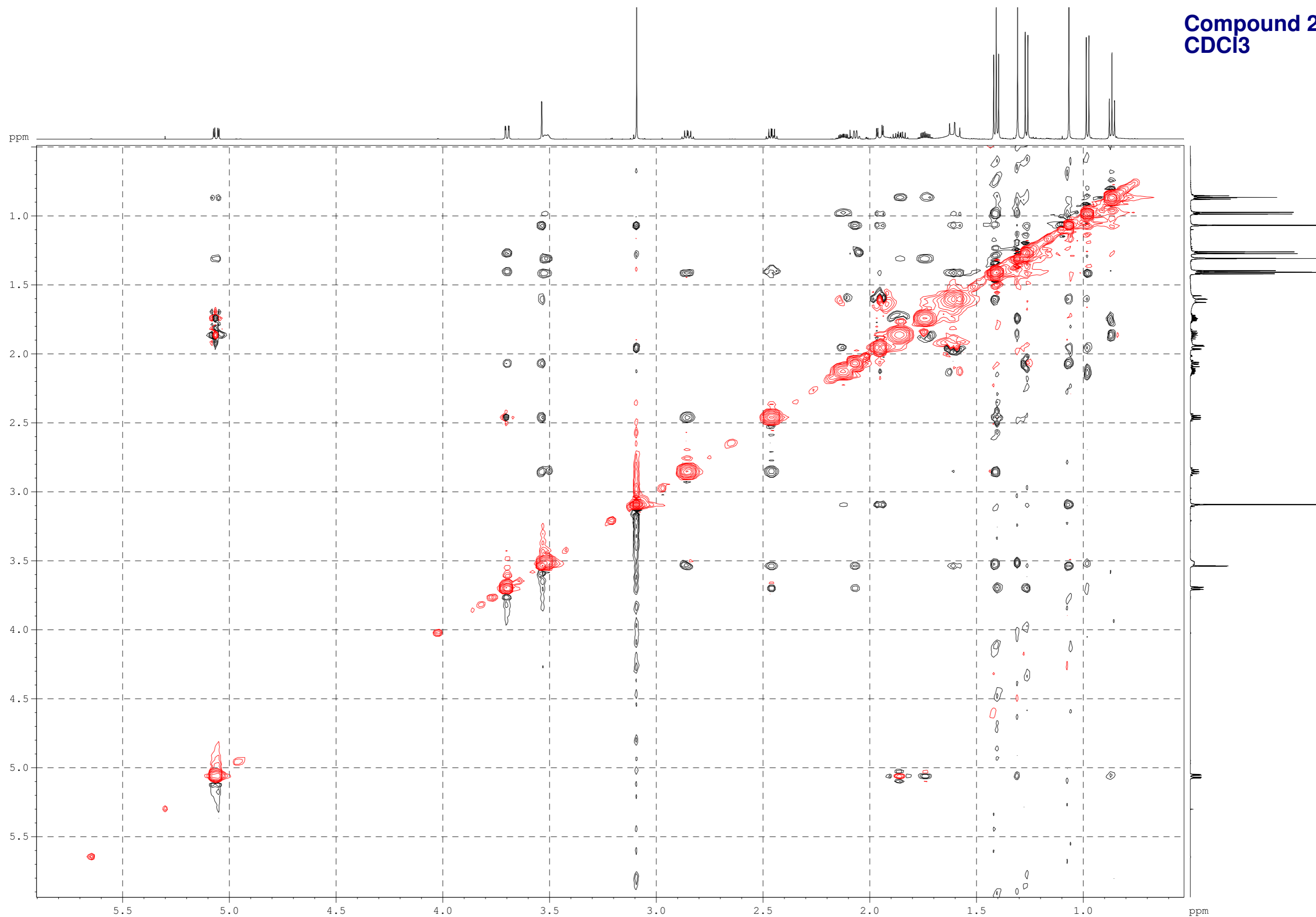

Supplement: File 3 — NMR spectra of compounds 2–4. [file Beilstein_J_Org_Chem-11-1447-s003.zip › NMRspectra/cpd2_NOESY_cdcl3.pdf]

**Compound 2**  
**DMSO**

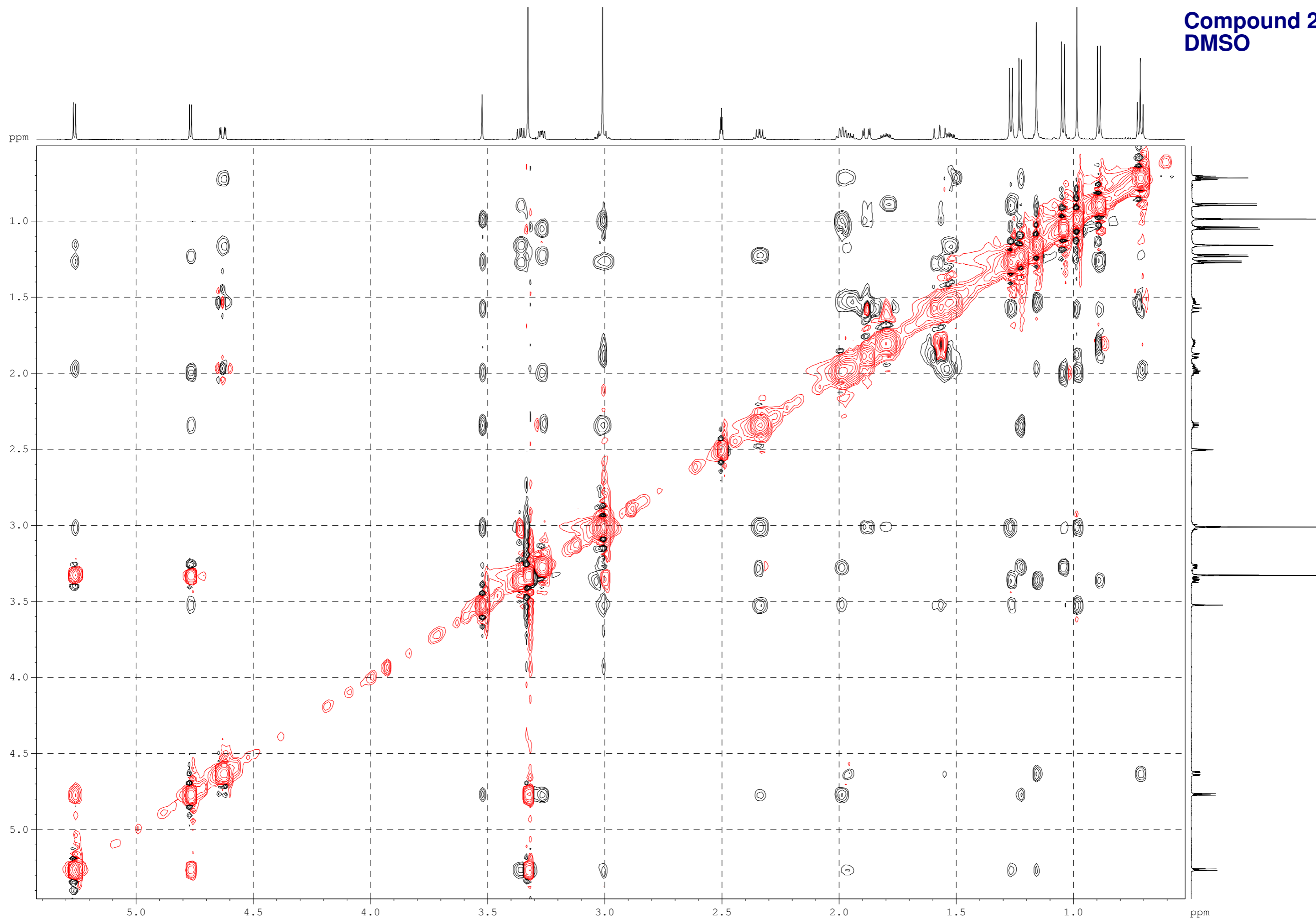

Supplement: File 3 — NMR spectra of compounds 2–4. [file Beilstein_J_Org_Chem-11-1447-s003.zip › NMRspectra/cpd2_NOESY_dmso.pdf]

Compound 3  
CDCl<sub>3</sub>

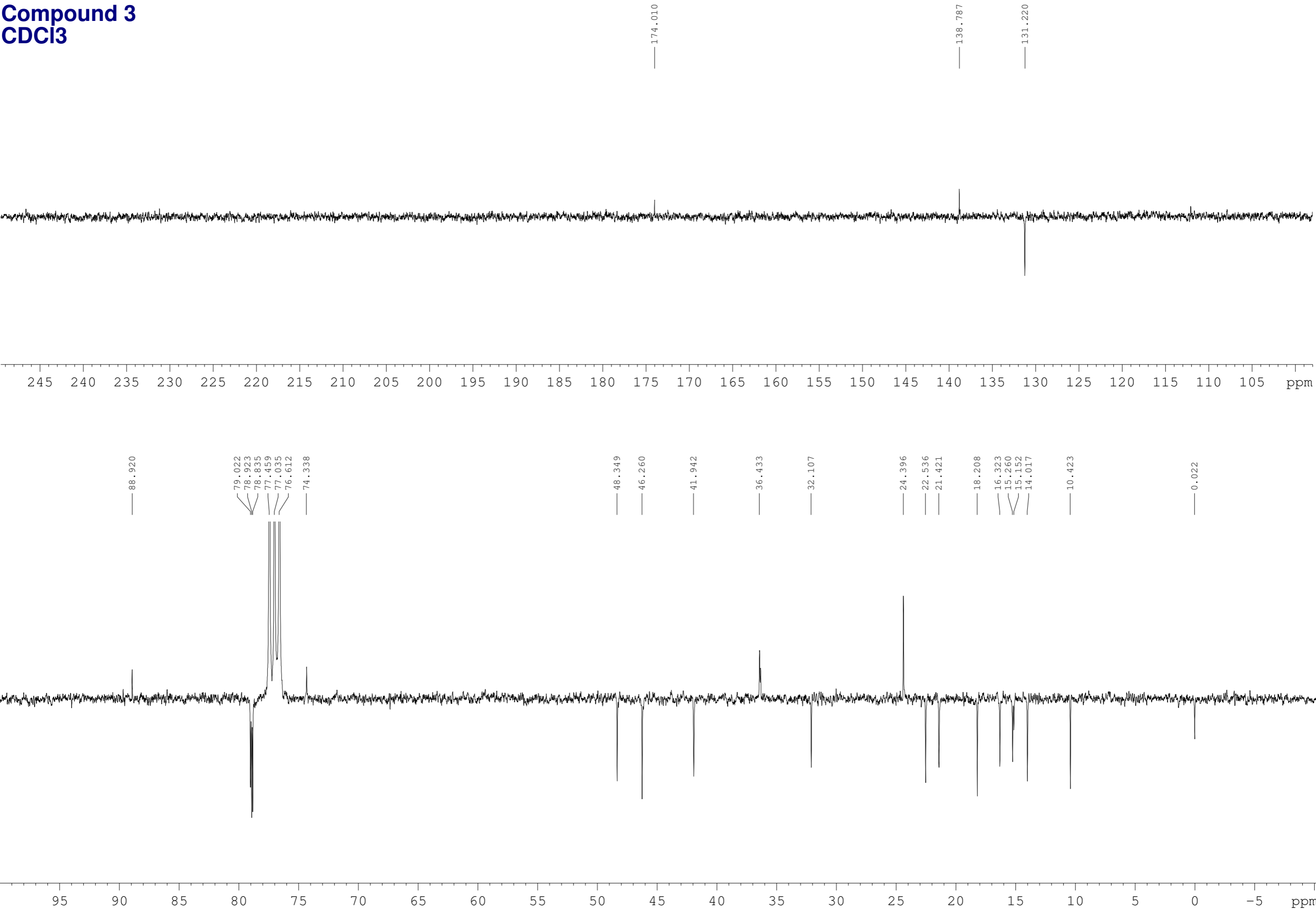

Supplement: File 3 — NMR spectra of compounds 2–4. [file Beilstein_J_Org_Chem-11-1447-s003.zip › NMRspectra/cpd3_13C_cdcl3.pdf]

Compound 3  
CDCl3

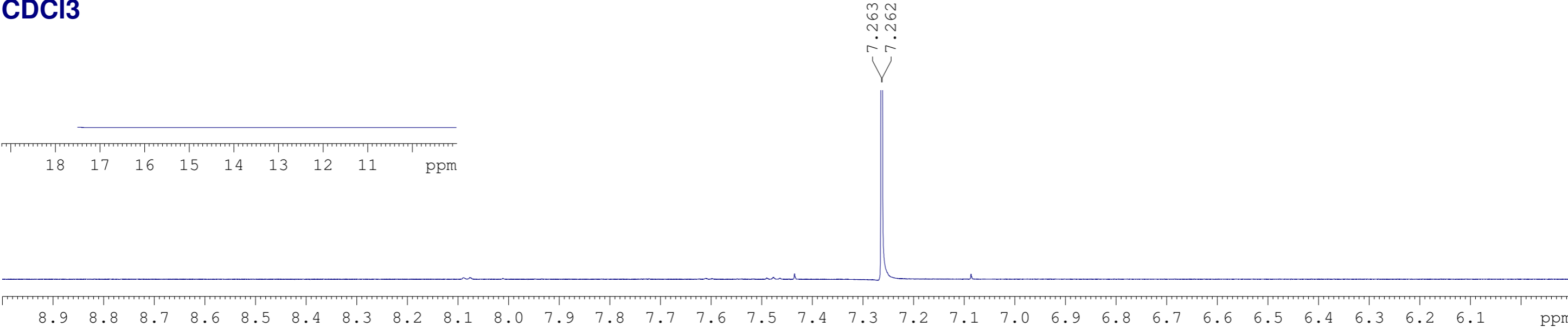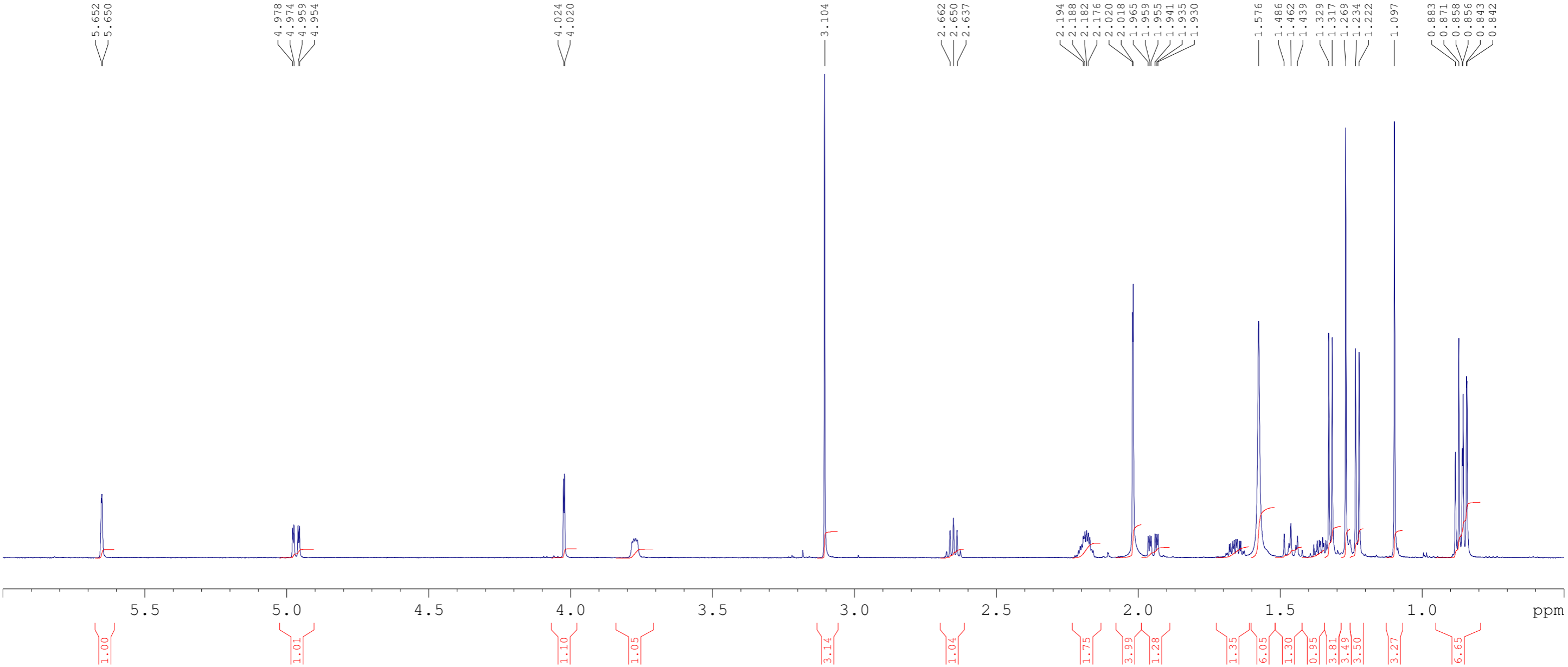

Supplement: File 3 — NMR spectra of compounds 2–4. [file Beilstein_J_Org_Chem-11-1447-s003.zip › NMRspectra/cpd3_1H_cdcl3.pdf]

Compound 3  
CDCl<sub>3</sub>

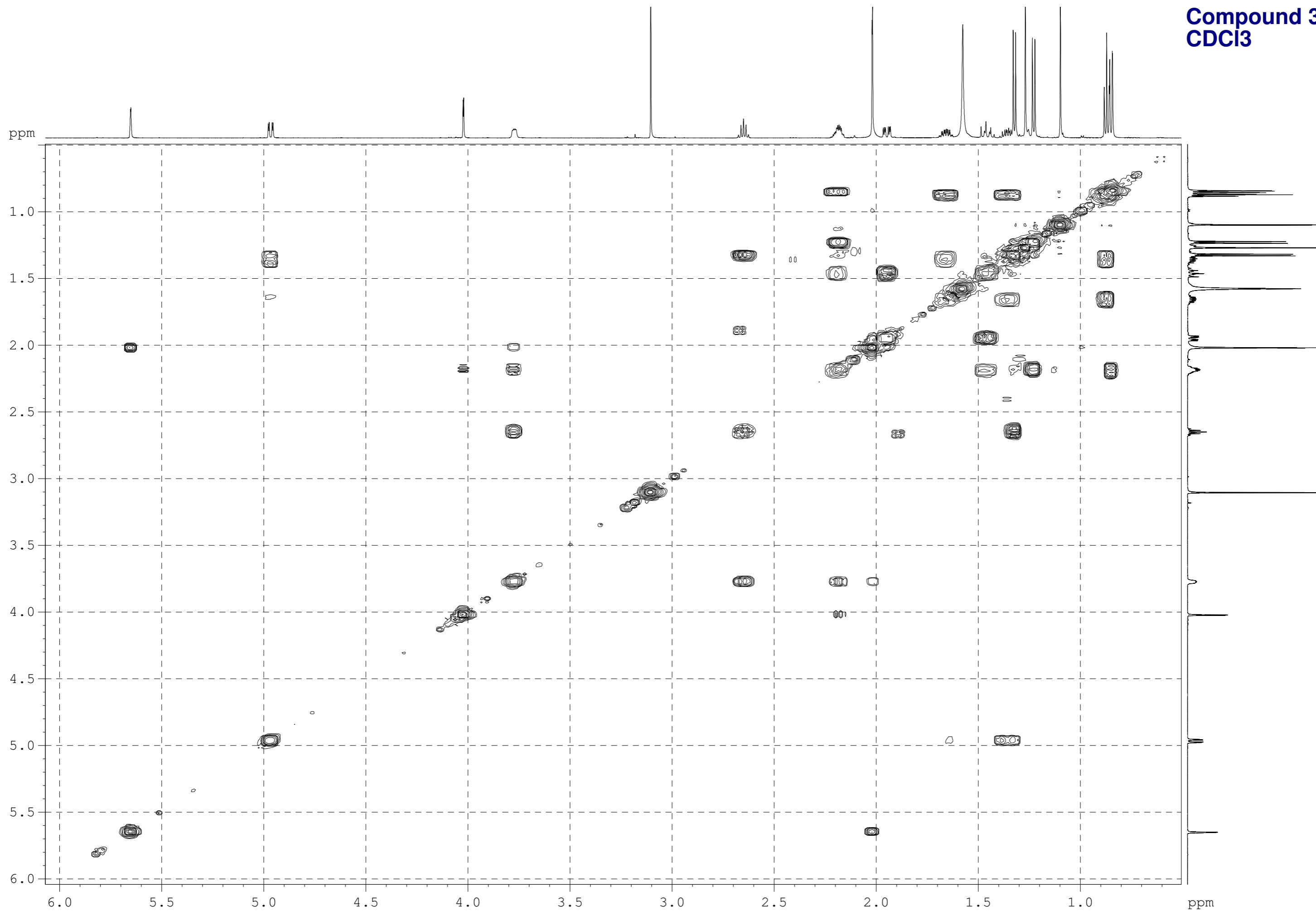

Supplement: File 3 — NMR spectra of compounds 2–4. [file Beilstein_J_Org_Chem-11-1447-s003.zip › NMRspectra/cpd3_COSY_cdcl3.pdf]

Compound 3  
CDCl<sub>3</sub>

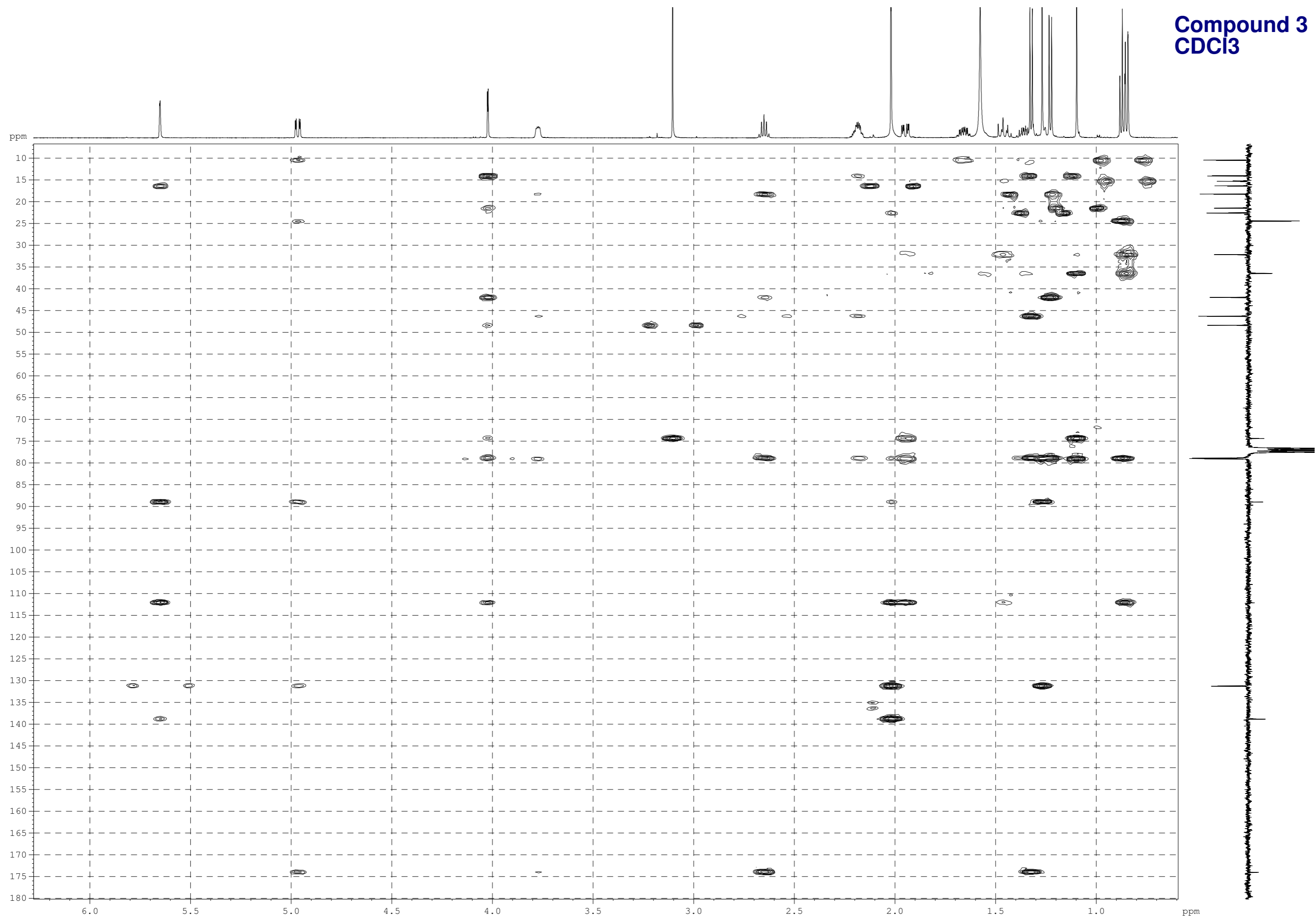

Supplement: File 3 — NMR spectra of compounds 2–4. [file Beilstein_J_Org_Chem-11-1447-s003.zip › NMRspectra/cpd3_HMBC_cdcl3.pdf]

Compound 3  
CDCl<sub>3</sub>

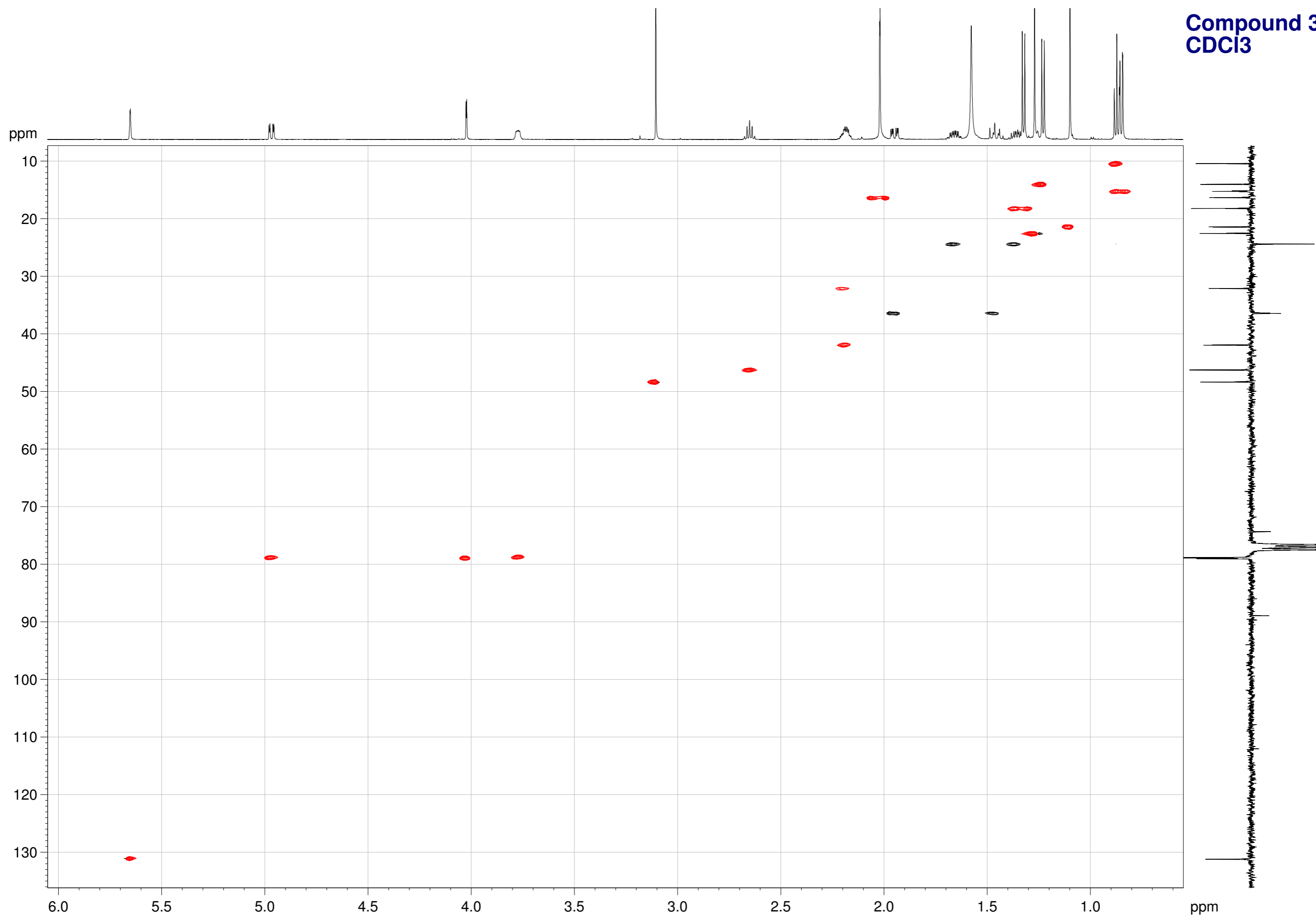

Supplement: File 3 — NMR spectra of compounds 2–4. [file Beilstein_J_Org_Chem-11-1447-s003.zip › NMRspectra/cpd3_HSQC_cdcl3.pdf]

Compound 3  
CDCl<sub>3</sub>

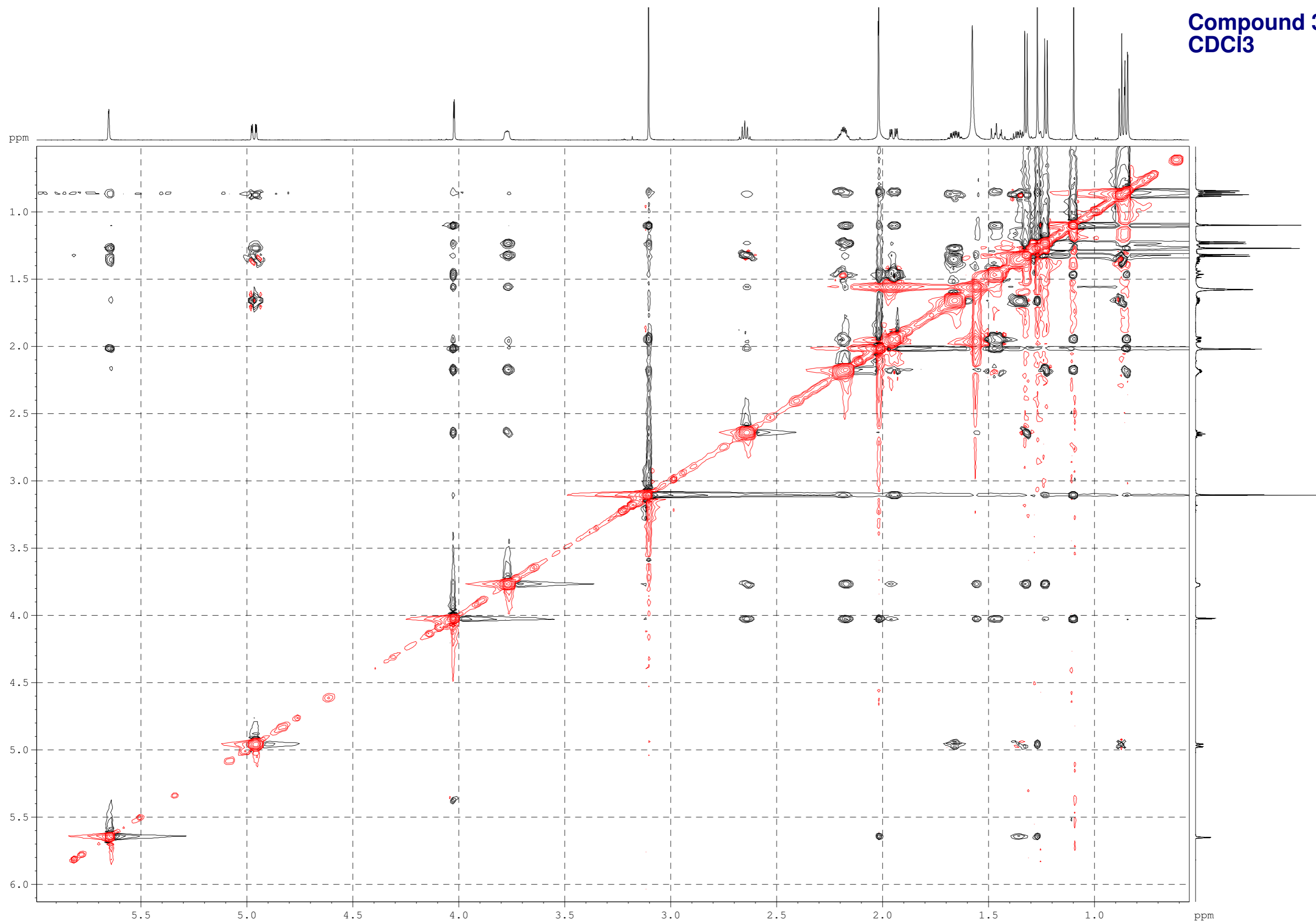

Supplement: File 3 — NMR spectra of compounds 2–4. [file Beilstein_J_Org_Chem-11-1447-s003.zip › NMRspectra/cpd3_NOESY_cdcl3.pdf]

Compound 4  
CDCl<sub>3</sub>

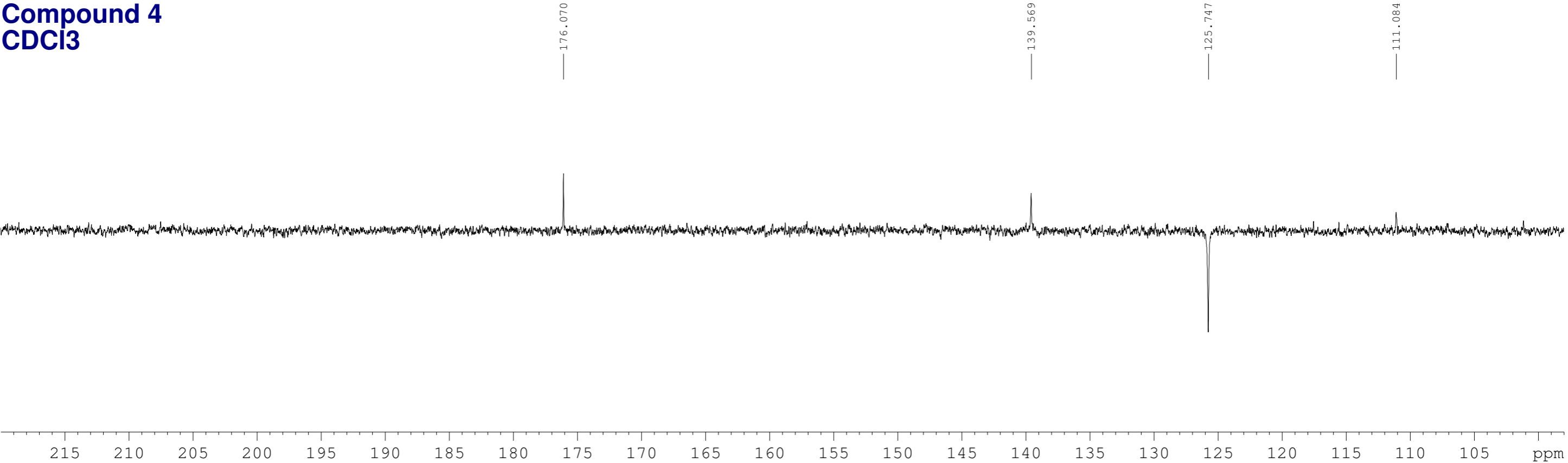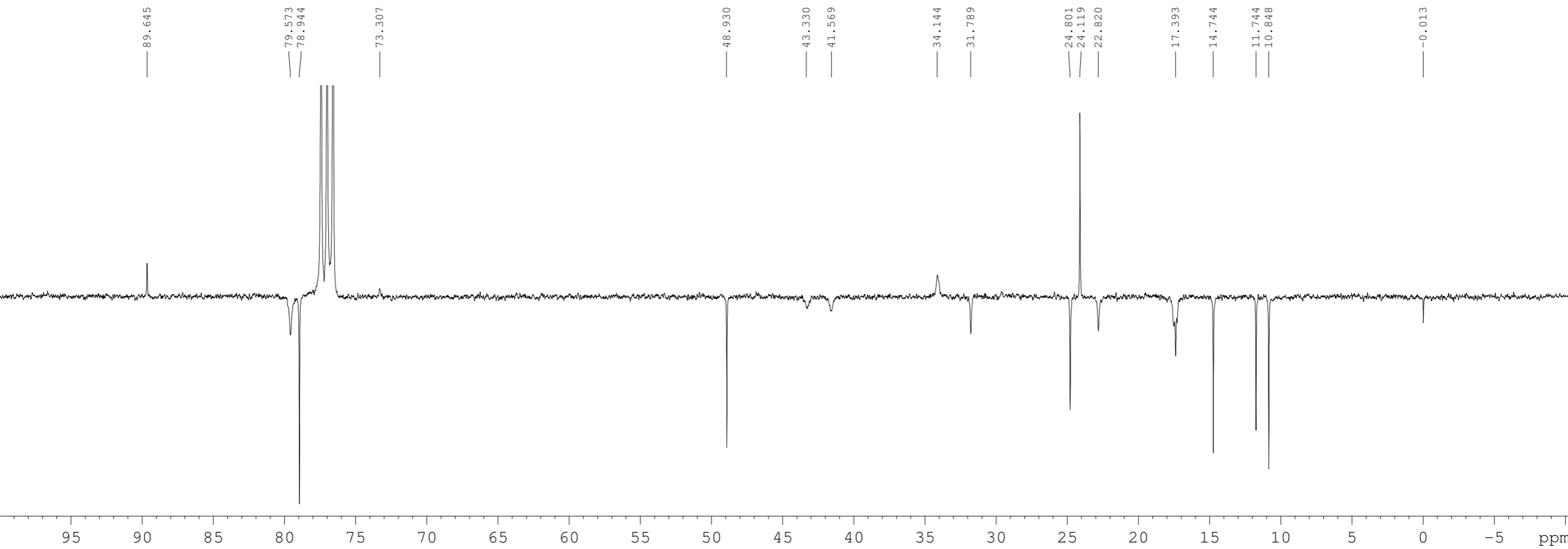

Supplement: File 3 — NMR spectra of compounds 2–4. [file Beilstein_J_Org_Chem-11-1447-s003.zip › NMRspectra/cpd4_13C_cdcl3.pdf]

**Compound 4**  
**DMSO**

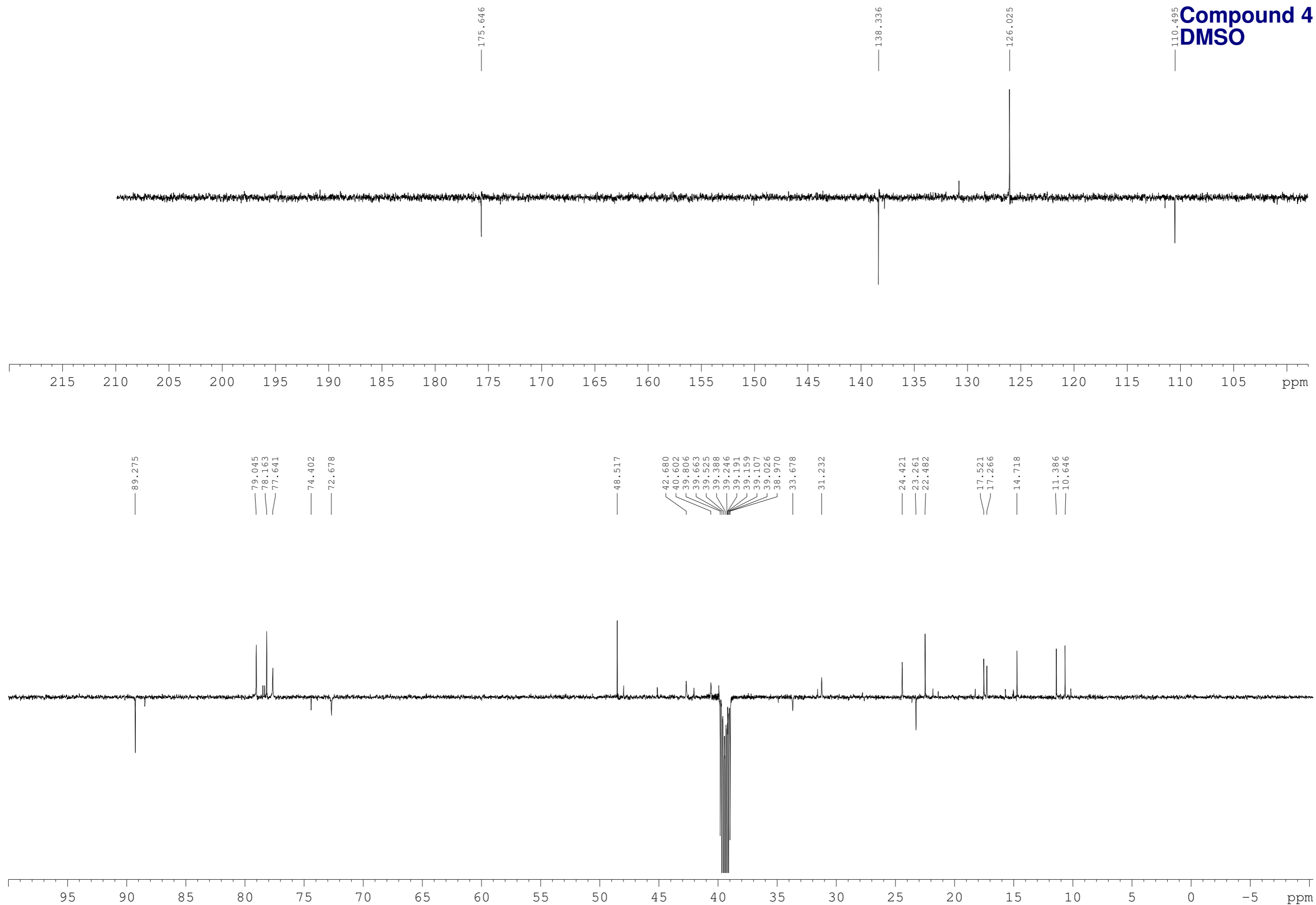

Supplement: File 3 — NMR spectra of compounds 2–4. [file Beilstein_J_Org_Chem-11-1447-s003.zip › NMRspectra/cpd4_13C_dmso.pdf]

Compound 4  
CDCl<sub>3</sub>

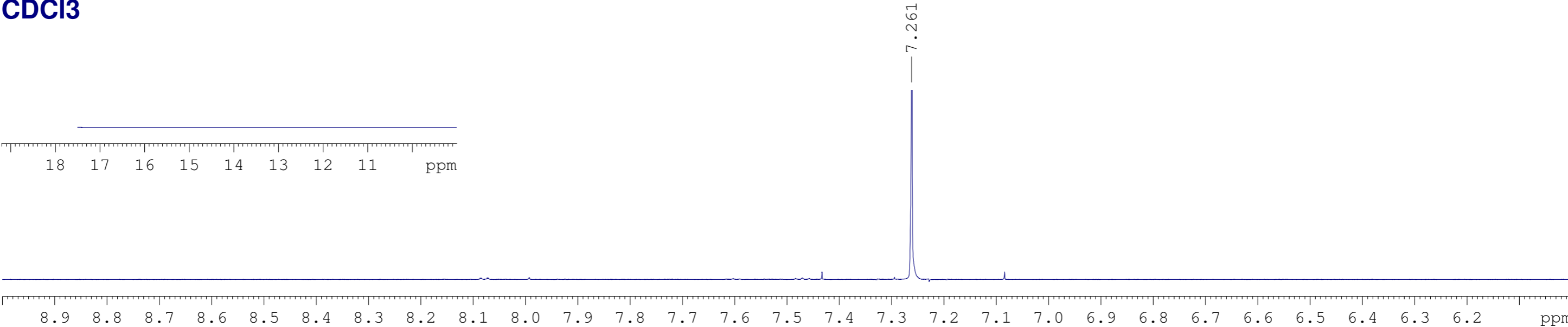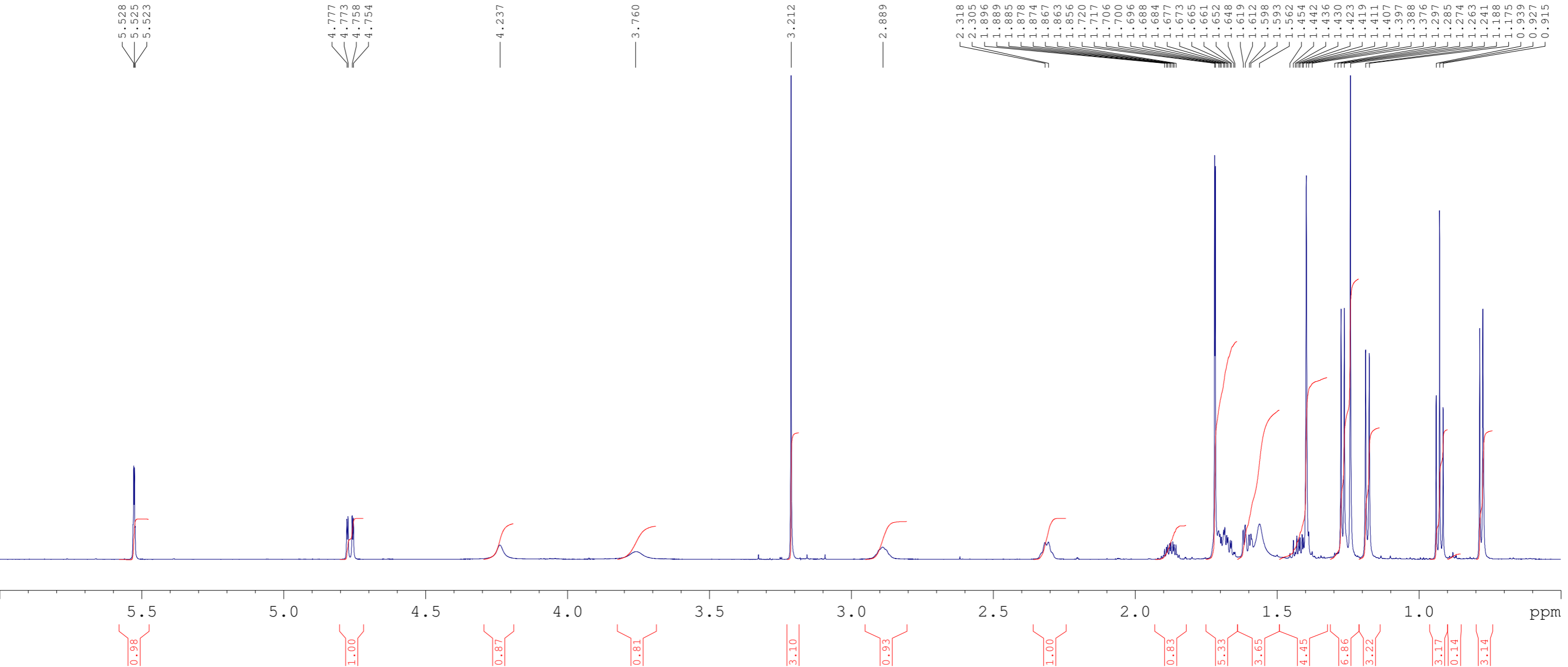

Supplement: File 3 — NMR spectra of compounds 2–4. [file Beilstein_J_Org_Chem-11-1447-s003.zip › NMRspectra/cpd4_1H_cdcl3.pdf]

Compound 4  
DMSO

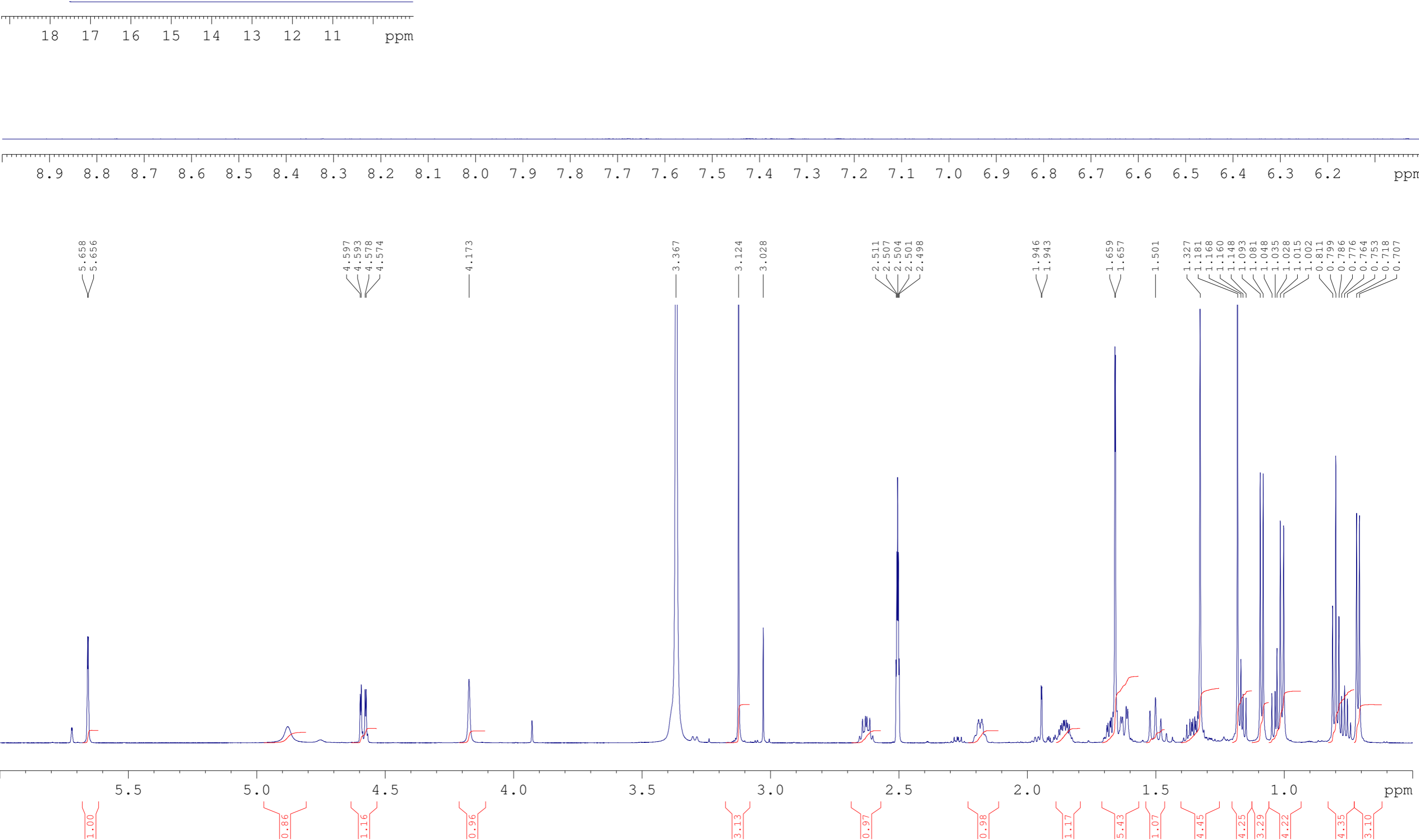

Supplement: File 3 — NMR spectra of compounds 2–4. [file Beilstein_J_Org_Chem-11-1447-s003.zip › NMRspectra/cpd4_1H_dmso.pdf]

Compound 4  
CDCl<sub>3</sub>

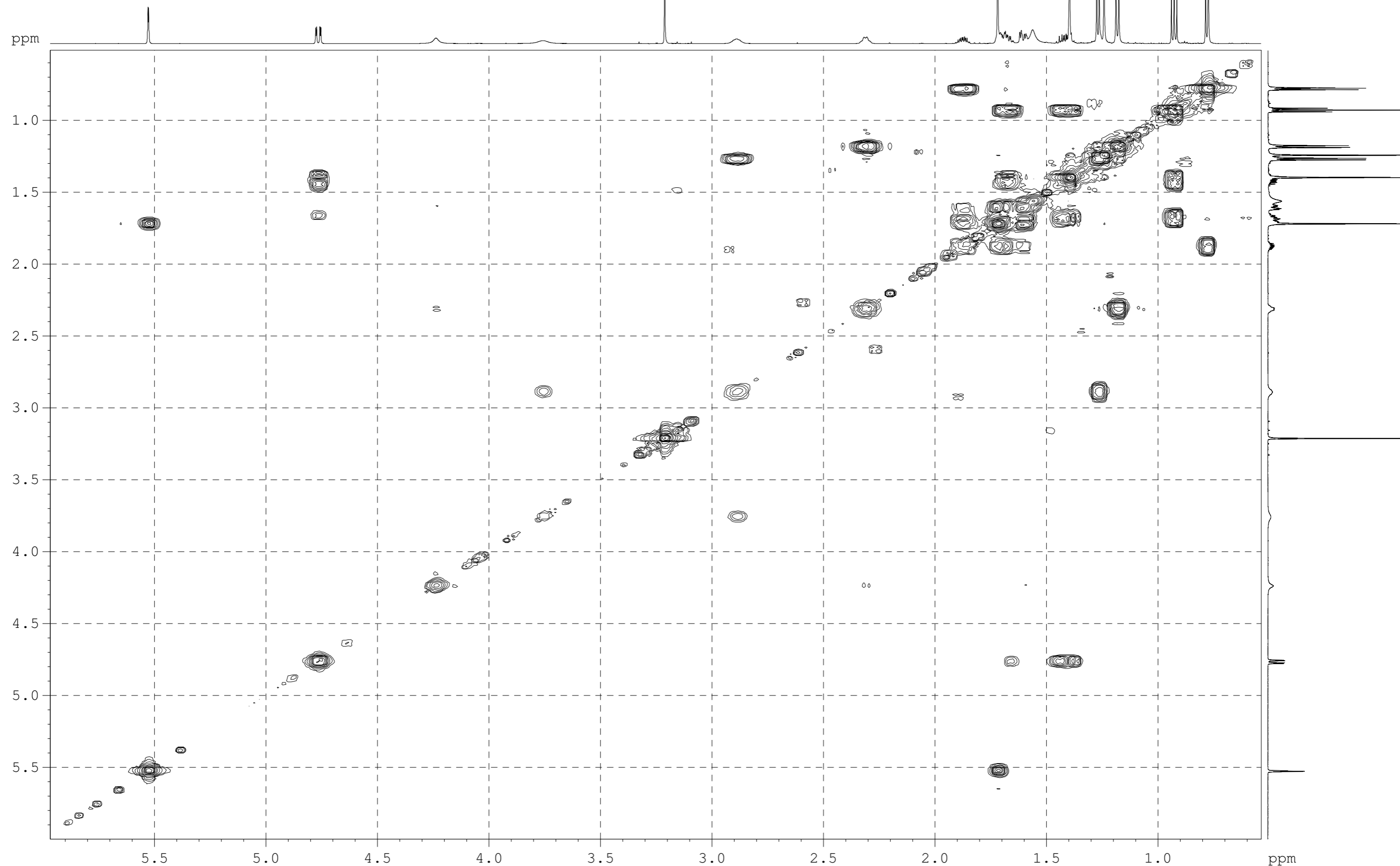

Supplement: File 3 — NMR spectra of compounds 2–4. [file Beilstein_J_Org_Chem-11-1447-s003.zip › NMRspectra/cpd4_COSY_cdcl3.pdf]

Compound 4  
DMSO

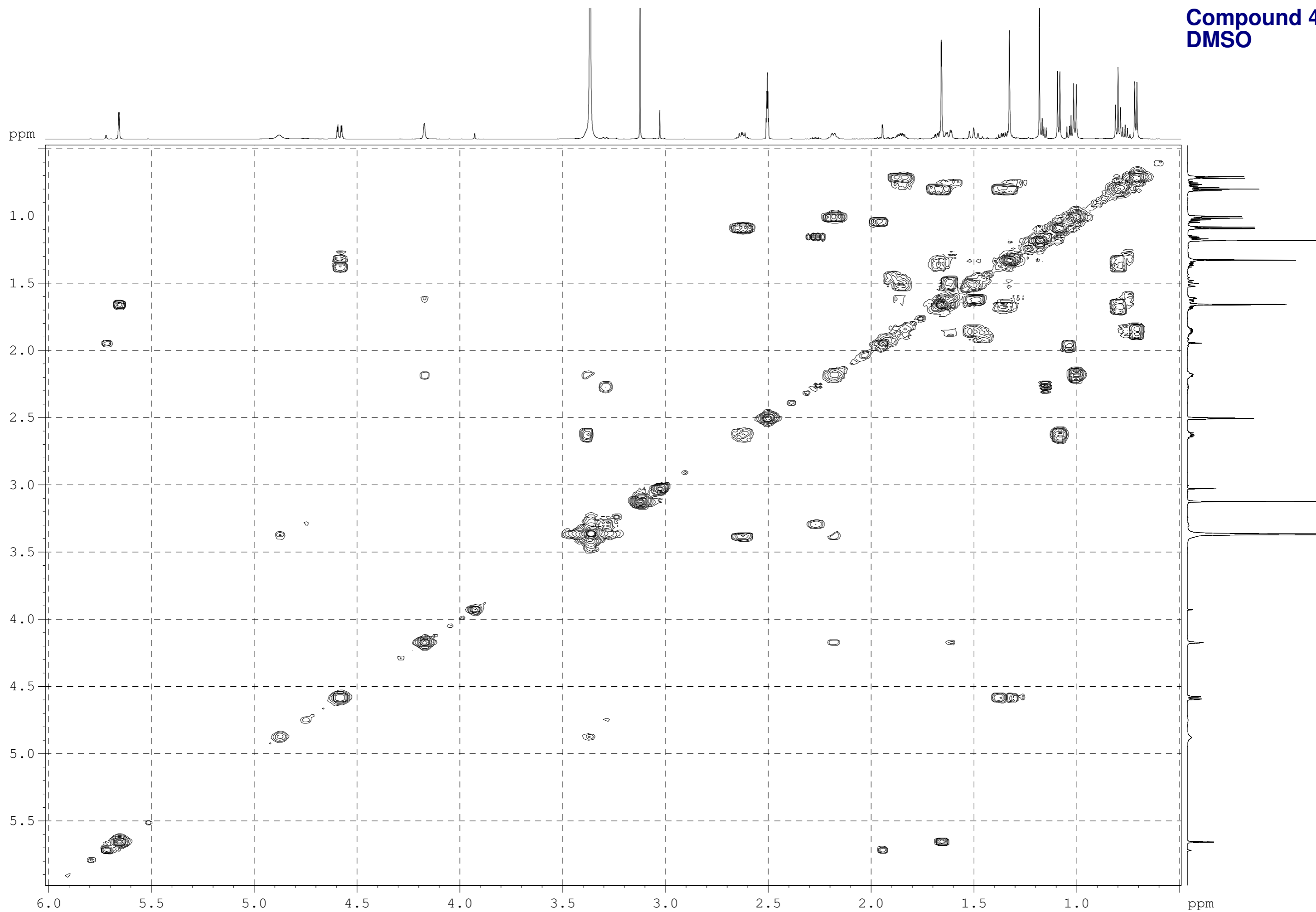

Supplement: File 3 — NMR spectra of compounds 2–4. [file Beilstein_J_Org_Chem-11-1447-s003.zip › NMRspectra/cpd4_COSY_dmso.pdf]

Compound 4  
CDCl<sub>3</sub>

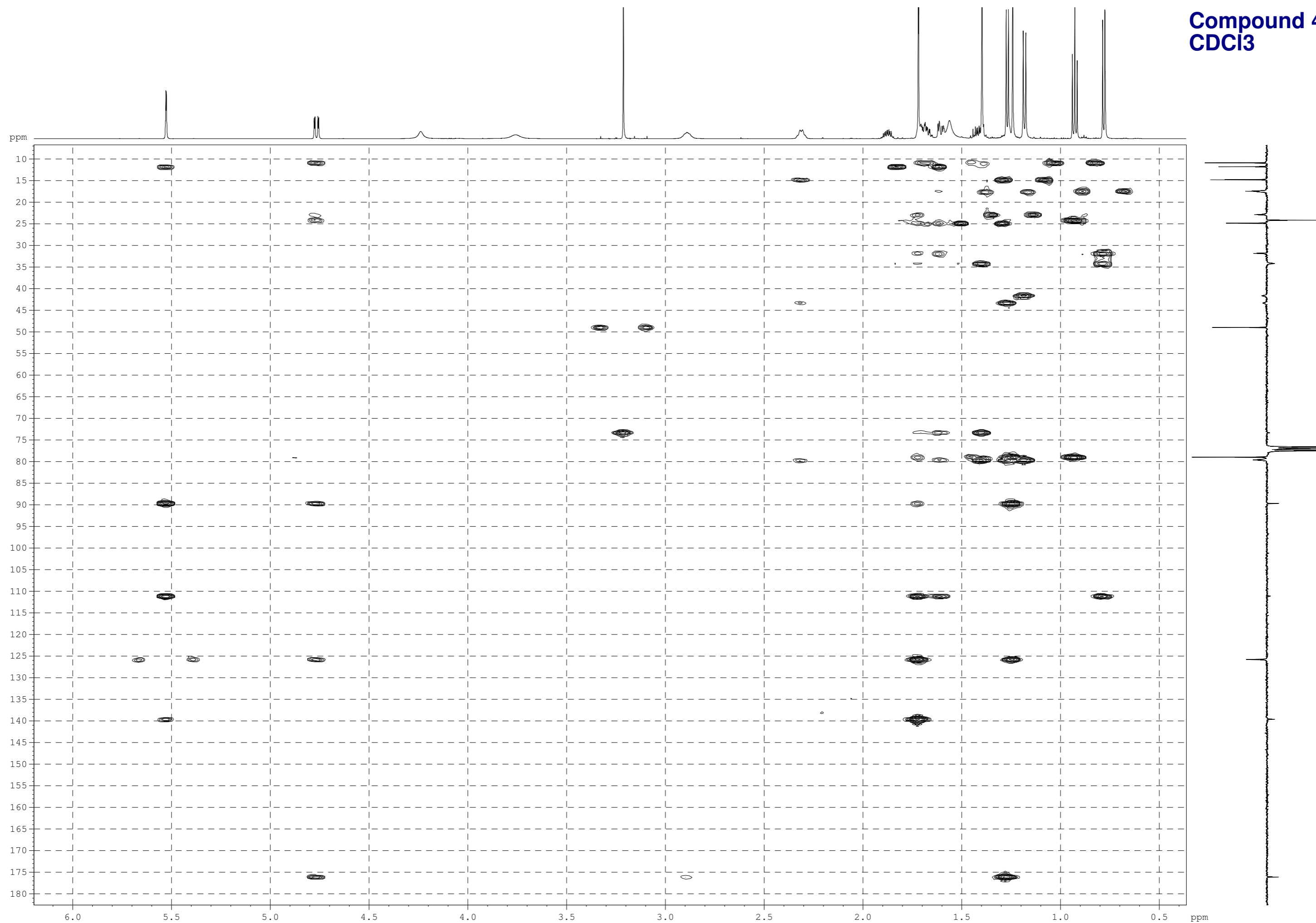

Supplement: File 3 — NMR spectra of compounds 2–4. [file Beilstein_J_Org_Chem-11-1447-s003.zip › NMRspectra/cpd4_HMBC_cdcl3.pdf]

Compound 4  
DMSO

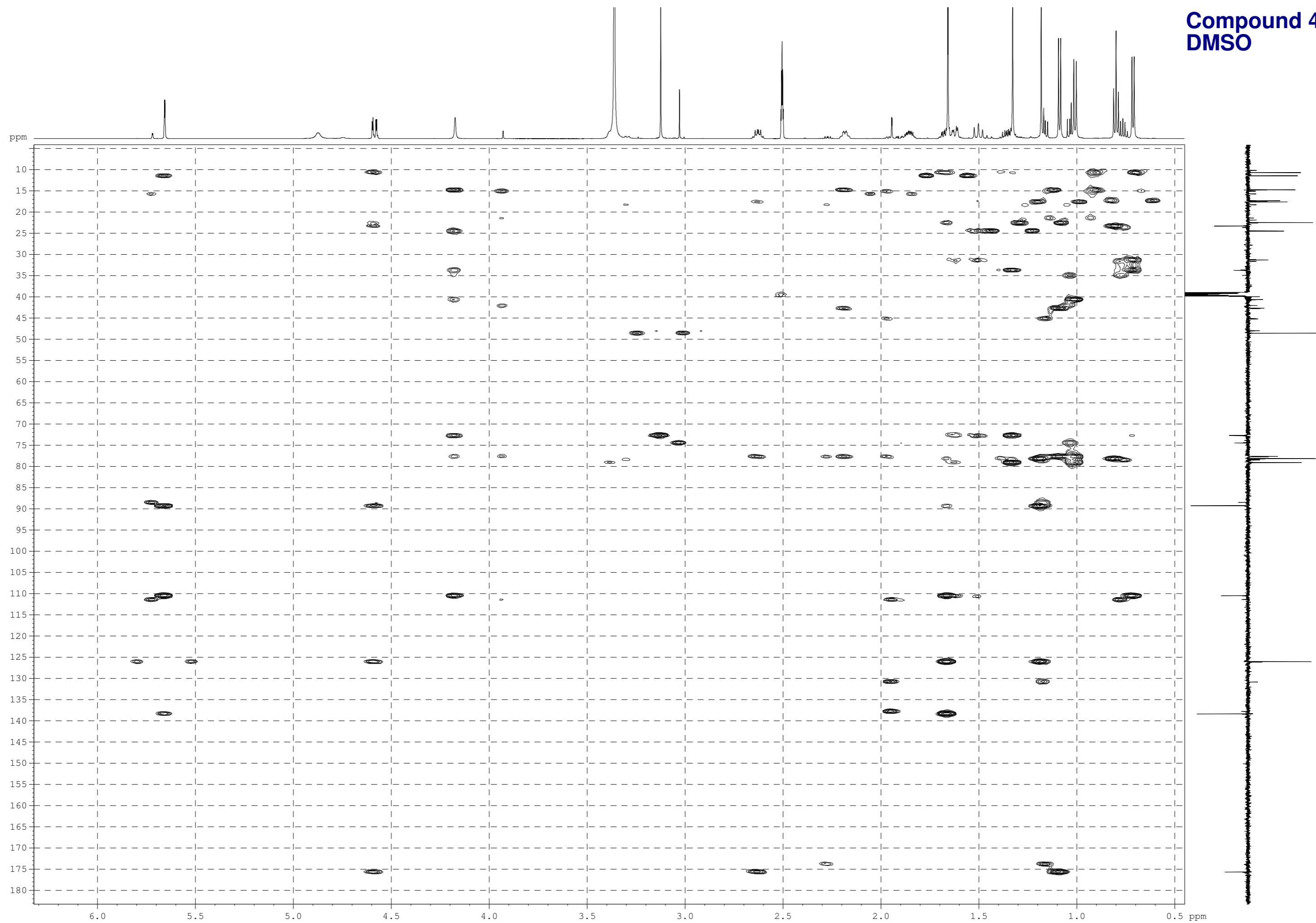

Supplement: File 3 — NMR spectra of compounds 2–4. [file Beilstein_J_Org_Chem-11-1447-s003.zip › NMRspectra/cpd4_HMBC_dmso.pdf]

Compound 4  
CDCl<sub>3</sub>

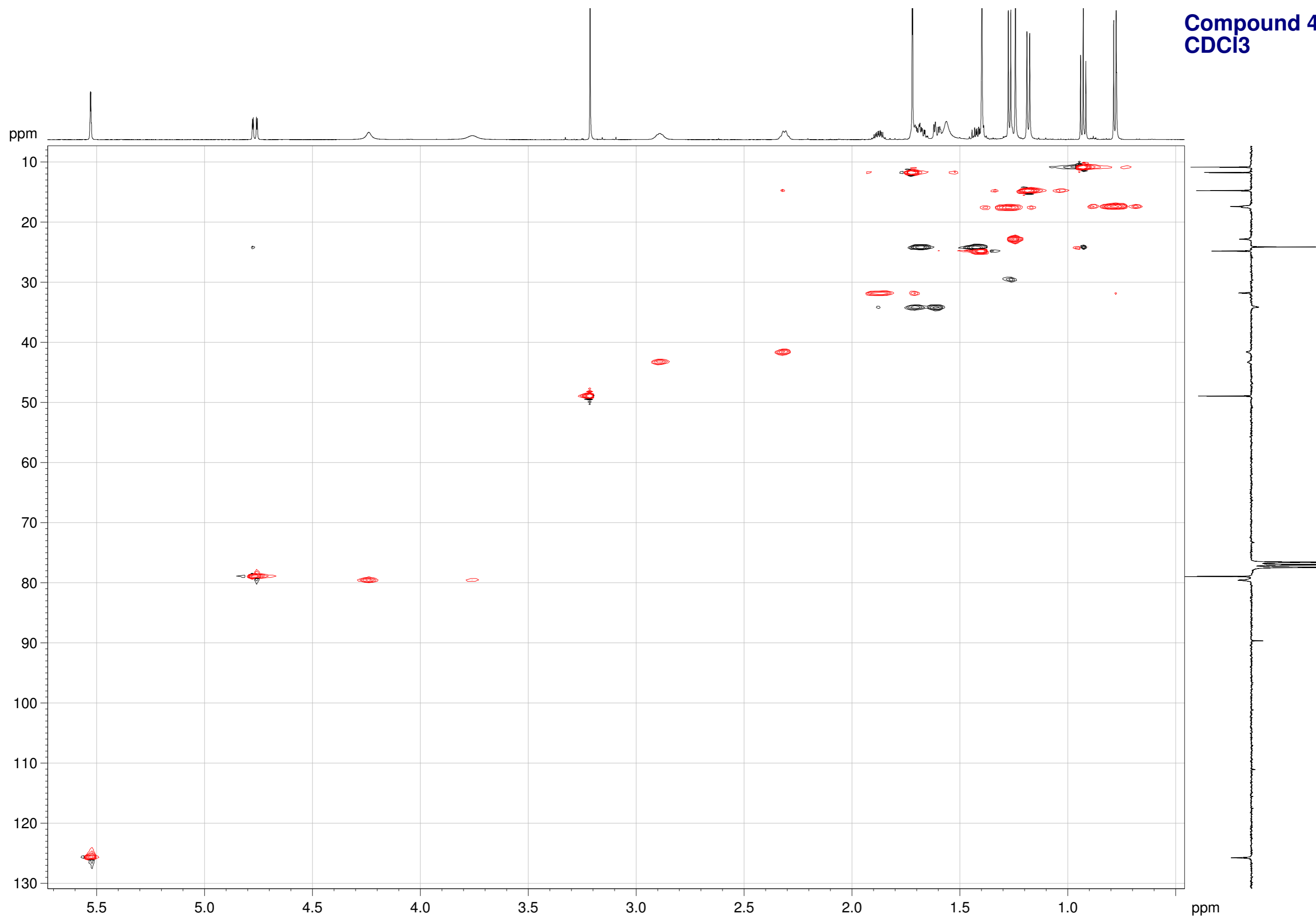

Supplement: File 3 — NMR spectra of compounds 2–4. [file Beilstein_J_Org_Chem-11-1447-s003.zip › NMRspectra/cpd4_HSQC_cdcl3.pdf]

**Compound 4**  
**DMSO**

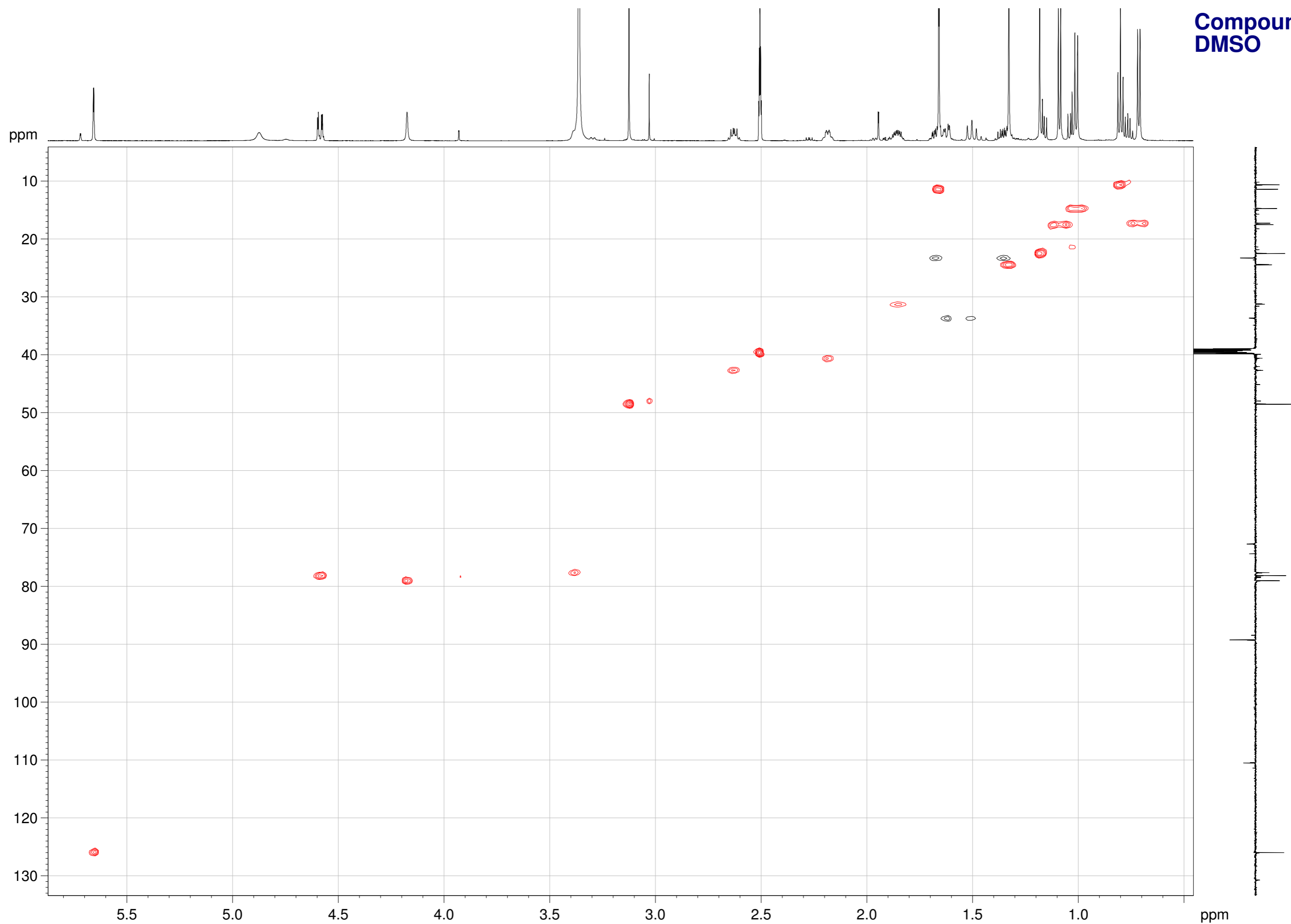

Supplement: File 3 — NMR spectra of compounds 2–4. [file Beilstein_J_Org_Chem-11-1447-s003.zip › NMRspectra/cpd4_HSQC_dmso.pdf]

**Compound 4**  
**DMSO**

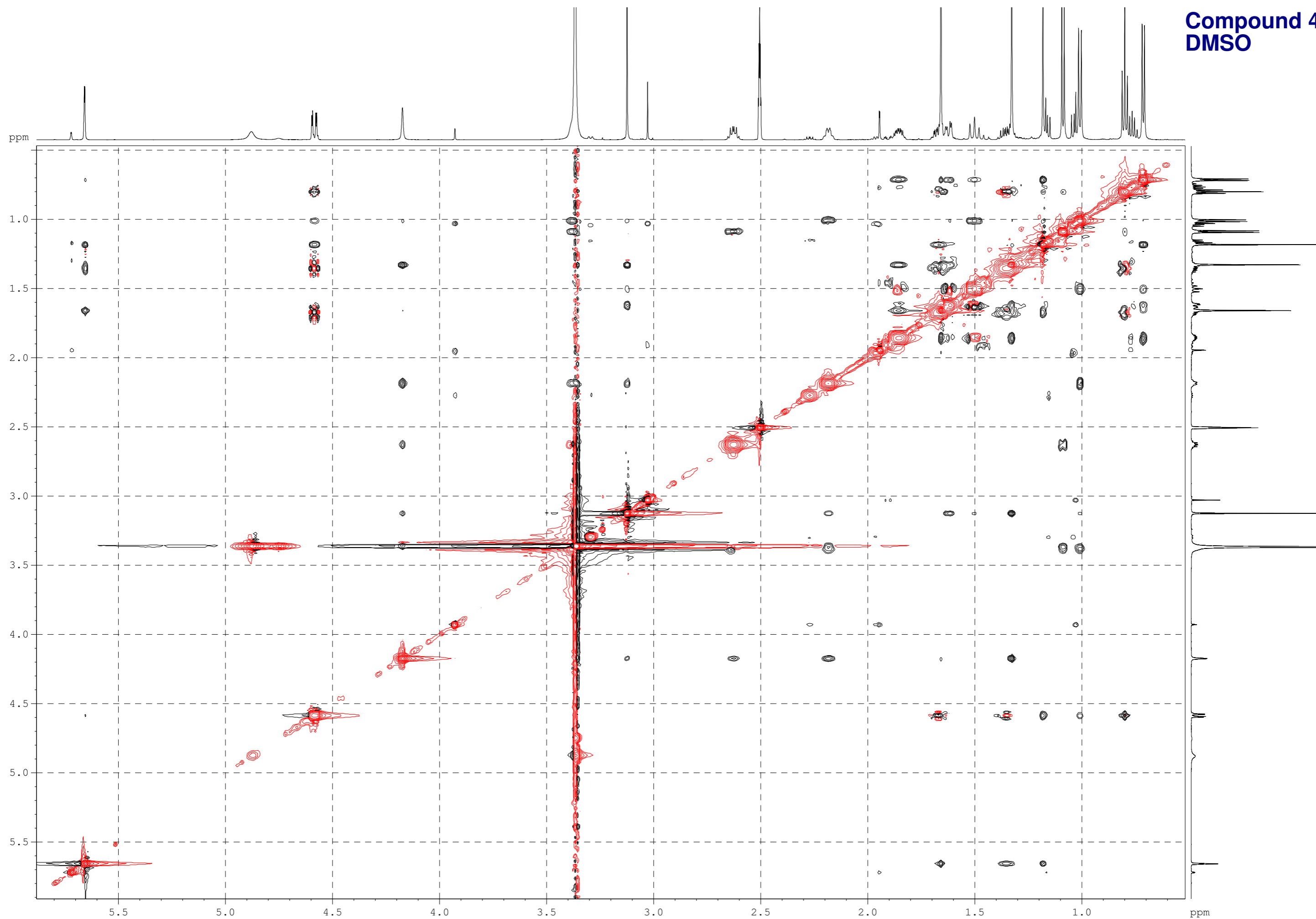

Supplement: File 3 — NMR spectra of compounds 2–4. [file Beilstein_J_Org_Chem-11-1447-s003.zip › NMRspectra/cpd4_NOESY_dmso.pdf]
